# LKB1‐SIK2 loss drives uveal melanoma proliferation and hypersensitivity to SLC8A1 and ROS inhibition

**DOI:** 10.15252/emmm.202317719

**Published:** 2023-11-15

**Authors:** Sarah Proteau, Imène Krossa, Chrystel Husser, Maxime Guéguinou, Federica Sella, Karine Bille, Marie Irondelle, Mélanie Dalmasso, Thibault Barouillet, Yann Cheli, Céline Pisibon, Nicole Arrighi, Sacha Nahon‐Estève, Arnaud Martel, Lauris Gastaud, Sandra Lassalle, Olivier Mignen, Patrick Brest, Nathalie M Mazure, Frédéric Bost, Stéphanie Baillif, Solange Landreville, Simon Turcotte, Dan Hasson, Saul Carcamo, Christophe Vandier, Emily Bernstein, Laurent Yvan‐Charvet, Mitchell P Levesque, Robert Ballotti, Corine Bertolotto, Thomas Strub

**Affiliations:** ^1^ University Côte d'Azur Nice France; ^2^ Inserm, Biology and Pathologies of melanocytes, team1, Equipe labellisée Ligue 2020, and Equipe labellisée ARC 2022, Mediterranean Centre for Molecular Medicine Nice France; ^3^ University of Tours Tours France; ^4^ Inserm, N2C UMR 1069 Tours France; ^5^ Department of Dermatology, University Hospital Zurich University of Zurich Zurich Switzerland; ^6^ Inserm, Hematometabolism and metainflammation, team 13, Mediterranean Centre for Molecular Medicine Nice France; ^7^ Department of Ophthalmology Centre Hospitalier Universitaire of Nice Nice France; ^8^ Centre Antoine Lacassagne Nice France; ^9^ Laboratory of Clinical and Experimental Pathology, University Hospital of Nice, FHU OncoAge, Cote d'Azur University, Biobank BB‐0033‐00025, IRCAN team 4, OncoAge FHU Nice France; ^10^ LBAI, UMR1227, Univ Brest, Inserm Brest France; ^11^ IRCAN team 4, Inserm, CNRS, FHU‐oncoAge, IHU‐RESPIRera Nice Nice France; ^12^ Inserm, Cancer, Metabolism and environment, team, Equipe labellisée Ligue 2022, Mediterranean Centre for Molecular Medicine Nice France; ^13^ Département d'ophtalmologie et d'ORL‐CCF, Faculté de médecine Université Laval Quebec City QC Canada; ^14^ CUO‐Recherche and Axe médecine régénératrice Centre de recherche du CHU de Québec‐Université Laval Quebec City QC Canada; ^15^ Centre de recherche sur le cancer de l'Université Laval Quebec City QC Canada; ^16^ Centre de recherche en organogénèse expérimentale de l'Université Laval/LOEX Quebec City QC Canada; ^17^ Cancer Axis Centre de recherche du Centre Hospitalier de l'Université de Montréal/Institut du cancer de Montréal Montréal QC Canada; ^18^ Hepato‐Pancreato‐Biliary Surgery and Liver Transplantation Service Centre hospitalier de l'Université de Montréal Montréal QC Canada; ^19^ Department of Oncological Sciences, Tisch Cancer Institute Icahn School of Medicine at Mount Sinai New York NY USA; ^20^ Tisch Cancer Institute Bioinformatics for Next Generation Sequencing (BiNGS) Facility Icahn School of Medicine at Mount Sinai New York NY USA

**Keywords:** calcium, LKB1, SIK2, SLC8A1, uveal melanoma, Cancer, Skin

## Abstract

Metastatic uveal melanomas are highly resistant to all existing treatments. To address this critical issue, we performed a kinome‐wide CRISPR‐Cas9 knockout screen, which revealed the LKB1‐SIK2 module in restraining uveal melanoma tumorigenesis. Functionally, LKB1 loss enhances proliferation and survival through SIK2 inhibition and upregulation of the sodium/calcium (Na^+^/Ca^2+^) exchanger SLC8A1. This signaling cascade promotes increased levels of intracellular calcium and mitochondrial reactive oxygen species, two hallmarks of cancer. We further demonstrate that combination of an SLC8A1 inhibitor and a mitochondria‐targeted antioxidant promotes enhanced cell death efficacy in LKB1‐ and SIK2‐negative uveal melanoma cells compared to control cells. Our study also identified an LKB1‐loss gene signature for the survival prognostic of patients with uveal melanoma that may be also predictive of response to the therapy combination. Our data thus identify not only metabolic vulnerabilities but also new prognostic markers, thereby providing a therapeutic strategy for particular subtypes of metastatic uveal melanoma.

## Graphical abstract

Most patients with metastatic uveal melanoma die within a year from diagnosis, highlighting an unmet clinical need. A genetic screen was launched to identify actionable vulnerabilities and uncovered the critical role of the kinases LKB1 and SIK2 in constraining metastatic uveal melanoma cell growth.

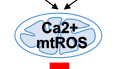

**The paper explained**

**Problem**

Uveal melanoma is the most frequent primary malignant tumor of the eye in adults. Up to 50% of the patients develop distant metastases, with the liver as the most frequent site. Liver metastases are highly resistant to existing treatments, and patients die within a year of diagnosis. These clinical data highlight the importance of finding relevant therapeutic targets to improve patient outcome.

**Results**

A genetic screen in metastatic uveal melanoma cells led to the identification of the kinases LKB1 and SIK2, whose deficiency promoted a massive increase in their proliferation. Downstream LKB1 or SIK2 loss, increased expression of the sodium/calcium exchanger SLC8A1 was a key mediator of their pro‐proliferative activity. Moreover, LKB1‐ or SIK2‐deficient cells exhibited enhanced intracellular calcium and mitochondrial ROS and were highly sensitive to a combination of SLC8A1 inhibitor and mitochondrial target antioxidant. Finally, we identified an LBK1 loss signature predictive of patient survival and response to this combination therapy.

**Impact**

Our study shows for the first time that the module involving loss of LKB1 and SIK2 and increase in SLC8A1 strongly stimulates the proliferative activity of metastatic uveal melanoma cells. LKB1 or SIK2 loss triggered enhanced intracellular calcium and mitochondrial ROS, which could be targeted to induce metastatic uveal melanoma cell apoptosis. This combination therefore represents a promising therapeutic strategy to treat metastatic uveal melanoma.

## Introduction

Uveal melanoma, the main primary intraocular malignancy in adults, is an aggressive and deadly neoplasm, which develops from melanocytes mainly in the choroid. At diagnosis, only 1–3% of the patients have detectable metastases (Carvajal *et al*, 2017). However, despite successful treatment of the primary tumor, up to 50% of patients develop metastases, predominantly to the liver (Garg *et al*, 2022). Uveal melanoma metastases are highly refractory to all therapies, even those that improve the clinical outcomes of patients with cutaneous melanoma because they are biologically and genetically distinct tumors (Pandiani *et al*, 2017). Recently, tebentafusp treatment, a novel immunotherapy, has been shown for the first time to improve the overall survival of patients with metastatic uveal melanoma (Nathan *et al*, 2021). However, tebentafusp treatment is limited to *HLA*‐A*02:01‐positive patients and demonstrated benefit in a small subset of patients (Nathan *et al*, 2021). To date, 90% of patients with metastatic uveal melanoma die within 6 months after diagnosis of metastases, highlighting an unmet clinical need.

Uveal melanoma is driven by oncogenic mutations in the heterotrimeric G protein subunit α (GNAQ) and in its paralog GNA11, which share > 90% peptide sequence identity and strikingly similar effects (Onken *et al*, 2008; Van Raamsdonk *et al*, 2009, 2010). The most frequent GNAQ and GNA11 mutation is the substitution of glutamine at position 209 by proline or leucine (GNAQ/11^Q209P/L^) that results in loss of GTPase activity producing constitutive activation of GNAQ/GNA11. Rare mutations in *CYSLTR2* and *PLCB4*, which function upstream and downstream of GNAQ/GNA11, respectively, have also been identified, demonstrating the importance of this pathway in uveal melanoma oncogenesis (Robertson *et al*, 2017). GNAQ/11 mutations are almost always mutually exclusive and coupled to mutations that are prognostically significant of metastatic risk, the most frequent being a loss of function in BRCA1‐associated protein 1 (BAP1), which is associated with high metastatic risk and poor prognosis. Uveal melanomas are also associated with chromosomal imbalances, including monosomy of chromosome 3 and amplification of 8q (Field *et al*, 2018; Shain *et al*, 2018).

Over the past decades, advances in the understanding of the molecular mechanisms underlying uveal melanoma have been made. Oncogenic GNAQ, through ADP ribosylation factor 6 (ARF6), promotes activation of multiple downstream signaling pathways such as phospholipase C‐beta (PLC‐β)/protein kinase C (PKC)/extracellular signal‐regulated kinase (ERK) and trio‐Rho guanine nucleotide exchange factor (Trio)/RHO/RAC/yes‐associated protein (YAP) (Yoo *et al*, 2016; Chen *et al*, 2017; Pandiani *et al*, 2017). Oncogenic GNAQ triggers activation of other cascades such as the phosphatidylinositol 3‐kinase (PI3K)/protein kinase B (Akt)/mammalian target of rapamycin (mTOR) module and β‐catenin (Saraiva *et al*, 2005; Yoo *et al*, 2016). While clinical studies have evaluated drugs targeting these key signaling pathways, either alone or in combination, limited, if any, efficacy has been observed (Carvajal *et al*, 2023). Thus, these mechanistic advances have not yet translated into effective therapeutic strategies to prevent or eliminate metastasis. Hence, pivotal players in uveal melanoma metastasis that would be amenable to therapeutic intervention remain to be identified.

Here, we performed a CRISPR‐Cas9 kinome screen in metastatic uveal melanoma cells to identify exploitable vulnerabilities. Our data identify a novel kinase cascade that plays a key role in the control of metastatic uveal melanoma cell proliferation and survival, exerting its effects via the regulation of calcium and reactive oxygen species metabolism. Our work also identifies a prognostic molecular signature for patient survival that is also predictive of cellular response to a combination of drugs that affect calcium and ROS metabolism. Taken together, we have unveiled new potential therapeutic targets for uveal melanoma and a method to stratify those patients most likely to respond to these treatments.

## Results

### A genetic CRISPR‐Cas9 screen against the kinome identifies LKB1 as a key driver of uveal melanoma cell proliferation

**Figure EV1:**
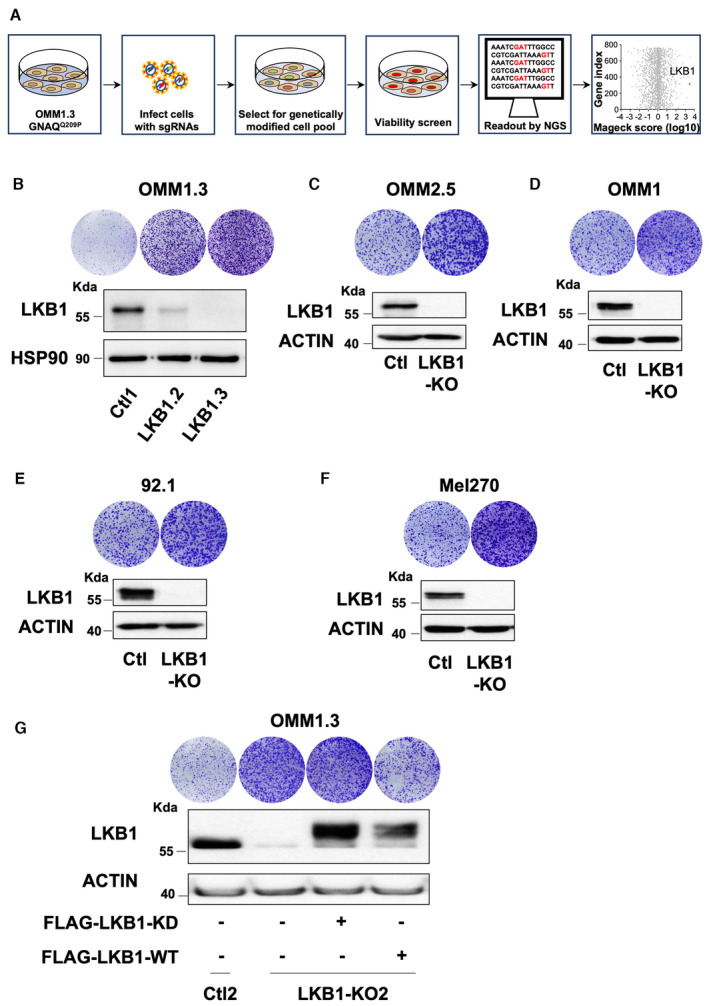
LKB1 deletion enhances metastatic uveal melanoma proliferation ASchematic of the CRISPR‐Cas9 kinome screen with Log_10_‐transformed MAGeCK robust ranking aggregation (RRA) scores for either depletion (left) or enrichment (right) of sgRNAs in OMM1.3 cells at D35 compared to D0.B(Bottom) Immunoblot of LKB1 in the indicated whole‐cell lysates of OMM1.3 pooled LKB1‐CRISPR cell lines. HSP90 was used as a loading control. (Top) Colony formation assay of OMM1.3 Ctl1 and pooled LKB1‐KD or KO cells grown for 10 days. Representative images of three independent experiments are shown.C–F(Bottom) Immunoblot of LKB1 in the indicated whole‐cell lysates of pooled LKB1‐CRISPR generated in human OMM2.5 and OMM1 metastatic uveal melanoma cells and human 92.1 and Mel270 primary uveal melanoma cells. B‐actin was used as a loading control. (Top) Colony formation assay of Ctl and pooled LKB1‐KO cells grown for 10 days. Representative images of three independent experiments are shown.GOMM1.3 Ctl2 and LKB1‐KO2 cells were noninfected (left) or infected with a vector‐encoding FLAG‐tagged kinase‐dead (FLAG‐LKB1‐KD) or wild‐type (FLAG‐LKB1‐WT) LKB1. Colony formation assay (top) and immunoblot (bottom) of LKB1 for the indicated cell lines are shown. B‐actin was used as a loading control. Representative images of three independent experiments are shown.

To identify new genes/pathways involved in metastatic uveal melanoma proliferation and survival, we conducted a CRISPR‐Cas9 knockout screen using the Human Kinome Brunello pooled sgRNAs library targeting ~ 760 kinases (Doench *et al*, 2016). Representative OMM1.3 uveal melanoma cells, originally derived from liver metastasis and harboring a GNAQ^Q209P^ mutation, were engineered to stably express Cas9, transduced with the single‐guide RNA (sgRNA) library (4 sgRNAs per gene encoded in pLKO.1) and subjected to puromycin selection. Next, genomic DNA was isolated from cells at day 0, which represents the library distribution prior to the screening selection process, and at day 35, the abundance of each sgRNA was determined using next‐generation sequencing. Analysis of the CRISPR‐Cas9 screen dataset with MaGeck software, which calculates a score based on a fold change, revealed depleted (left part of the volcano plot) or enriched (right part of the volcano plot) sgRNA compared to the control condition (Fig EV1A).

**Figure 1:**
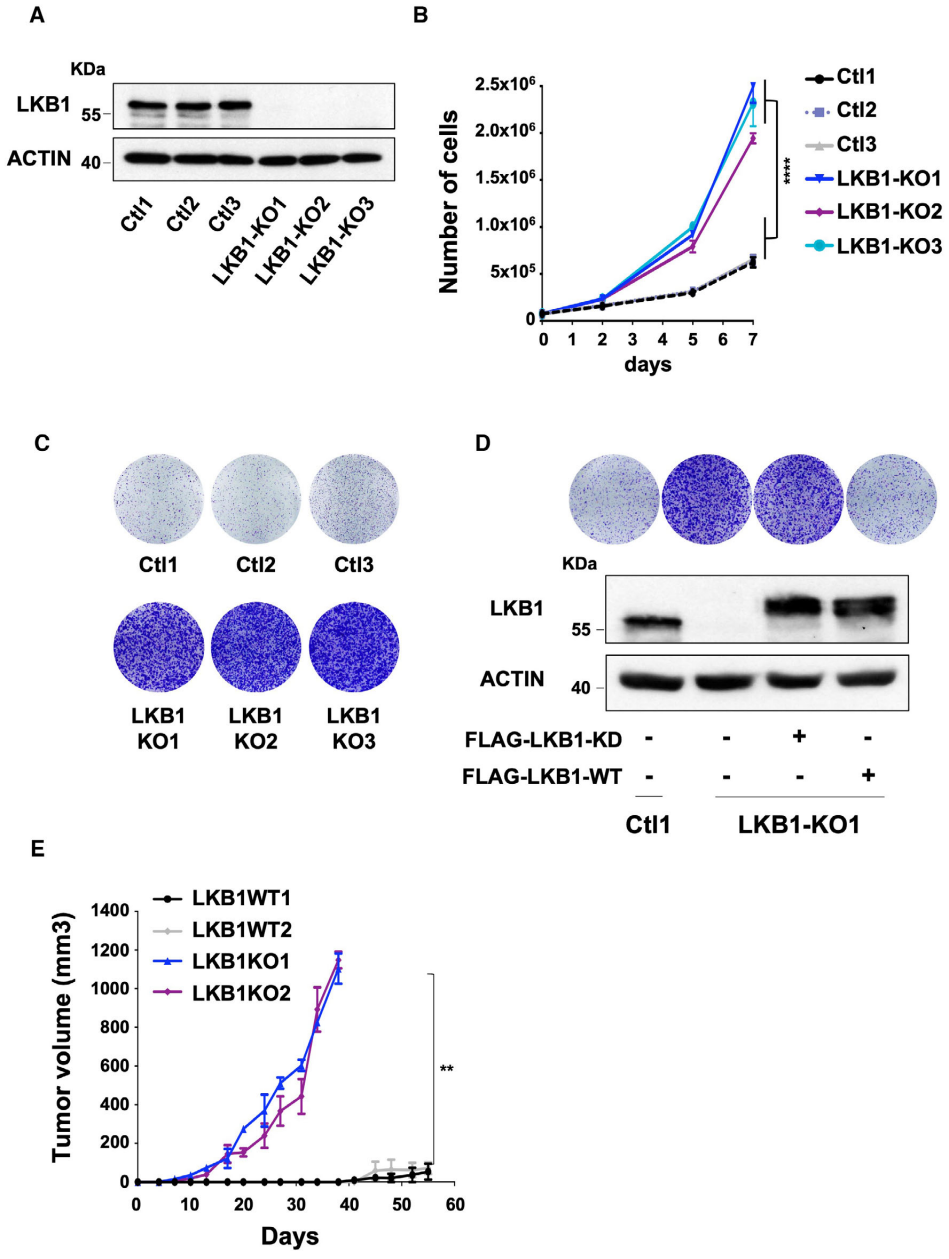
CRISPR‐Cas9 screen reveals LKB1 loss as a critical candidate in metastatic uveal melanoma cell proliferation Immunoblot of LKB1 in the indicated whole‐cell lysates of OMM1.3 Ctl or LKB1‐KO cells. Three different OMM1.3 Ctl and LKB1‐KO cell lines are shown. B‐actin was used as a loading control.Proliferation curves of OMM1.3 Ctl and LKB1‐KO cells. Data represent mean ± SD of three biological replicates (unpaired *t*‐test with Welsch's correction); ****P = 0.000041.Colony formation assay of OMM1.3 Ctl and LKB1‐KO cells grown for 10 days. Cells were seeded at low density. Representative images of three independent experiments are shown.OMM1.3 Ctl1 and LKB1‐KO1 cells were noninfected (left) or infected with a vector‐encoding FLAG‐tagged kinase dead (FLAG‐LKB1‐KD) or wild‐type (FLAG‐LKB1‐WT) LKB1. Colony formation assay (top) and immunoblot (bottom) of LKB1 for the indicated cell lines are shown. B‐actin was used as a loading control. Representative images of three independent experiments are shown.Quantification of tumor volume in nude mice bearing xenograft tumors of LKB1‐WT or LKB1‐KO OMM1.3 cells. Mann–Whitney test was performed for comparison between groups, *n* = 6. Data are mean ± SEM. **P = 0.0022. Source data are available online for this figure.

The screen yielded several valuable candidates (Dataset EV1), among the top hits was LKB1 (Fig EV1A), a well‐known tumor suppressor gene. The functional impact of LKB1 loss on proliferation was next validated by introducing the same individual sgRNAs used for the screen and deriving clonal cell lines. Partial or total loss of LKB1 in pooled OMM1.3 cells was confirmed by immunoblot and resulted in substantially increased colony‐forming capacity (Fig EV1B). We next generated clonal LKB1 knockout (KO) OMM1.3 cell lines (LKB1‐KO1, LKB1‐KO2, and LKB1‐KO3) as illustrated by complete LKB1 deficiency in immunoblot (Fig 1A). This resulted in a pronounced increase in growth rate (Fig 1B) and higher colony‐forming capacity compared to OMM1.3 control cell lines (Ctl1, Ctl2, and Ctl3) (Fig 1C). Validation of this finding was performed in additional cellular models including human metastatic uveal melanoma cells (OMM2.5 and OMM1) and primary uveal melanoma cells (92.1 and Mel270), demonstrating enhanced colony‐forming ability of LKB1‐KO (Fig EV1C–F). Next, we showed in two different LKB1‐KO clones that adding back the LKB1 wild‐type form but not a kinase‐dead mutant, both expressed at the same level, alleviated the hyperproliferative phenotype mediated by LKB1 loss (Figs 1D and EV1G). Thus, LKB1 kinase activity is indeed required for suppressing the growth effects. Next, to determine the role of LKB1 in tumorigenesis, we established xenograft tumors via subcutaneous inoculation of LKB1 wild‐type (Ctl) and LKB1‐KO OMM1.3 cells into the left flanks of nude mice. LKB1‐KO cells displayed a significant increase in tumor growth compared to LKB1‐wild‐type cells (Fig 1E). Collectively, these results indicate that LKB1 is critically required in constraining proliferation and tumorigenesis of uveal melanoma cells *in vitro* and *in vivo*.

**Figure 2:**
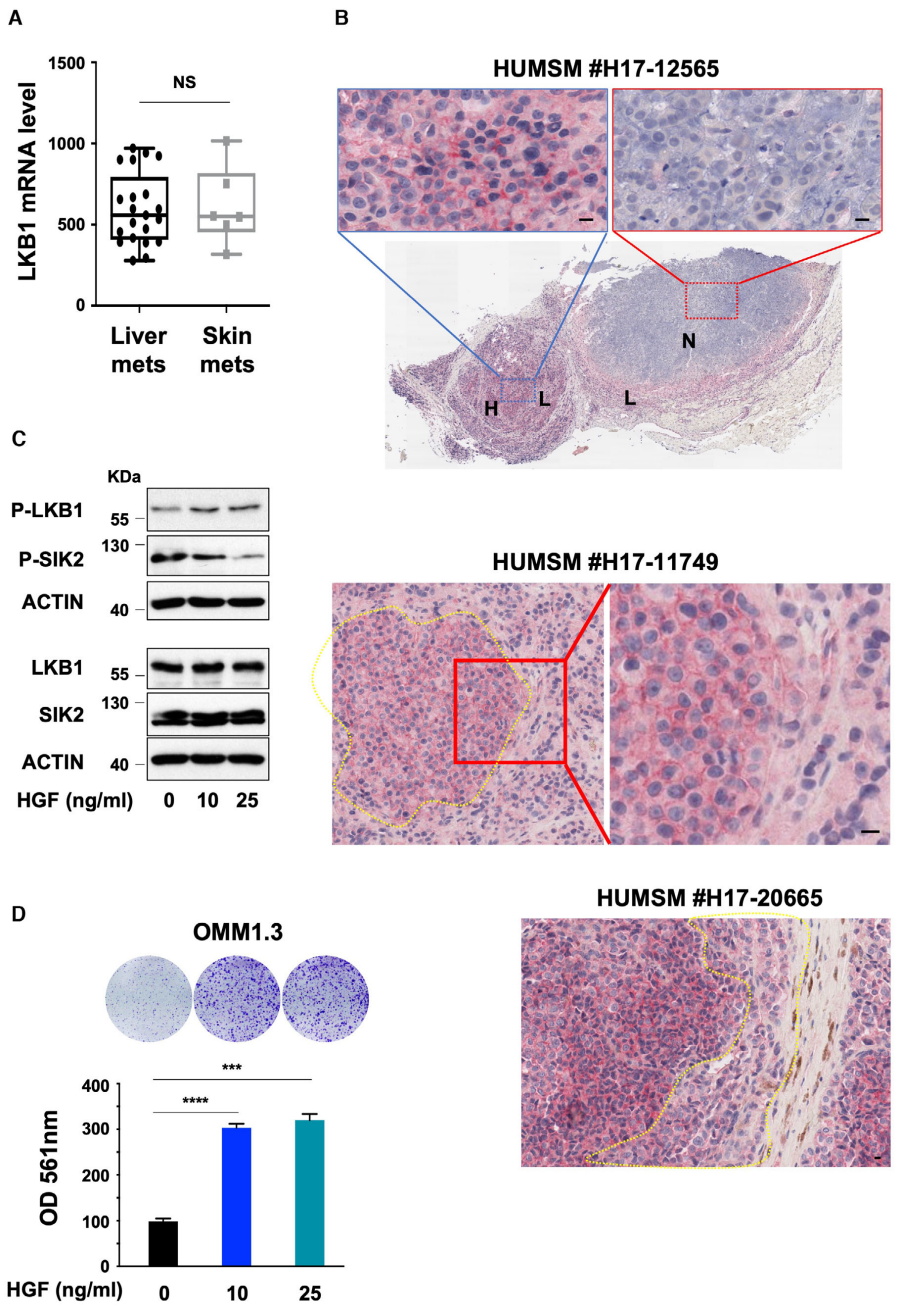
LKB1 expression is heterogeneous in human metastatic uveal melanoma Representative box and whisker plots of LKB1 mRNA level in liver and skin metastasis of human uveal melanomas. The central band denotes the median value, box contains interquartile ranges, while whiskers mark minimum and maximum values. Mann–Whitney test was performed for comparison between groups. All points are represented. NS, not significant, *P* = 0.8425. Data were obtained from Karlsson *et al* (2020)).Immunohistochemical stainings for LKB1 were performed on skin metastasis of human uveal melanomas. Staining intensity is represented as (H) High, (L) Low, and (N) Negative). Scale bars represent 10 μm.Immunoblot of LKB1 total or phosphorylated on serine 428 and of SIK2 total or phosphorylated on threonine 175 in OMM1.3 cells untreated or treated with HGF at 10 or 25 ng/ml for 10 days. B‐actin was used as a loading control. Representative images of three independent experiments are shown.Colony formation assay of OMM1.3 treated as in (C) grown for 10 days. Cells were seeded at low density. Representative images of three independent experiments are shown. Data represent mean ± SD of three biological replicates (unpaired *t*‐test with Welsch's correction); ***P = 0.0004, ****P = 0.000001. Source data are available online for this figure.

LKB1 tumor suppressor activity is inactivated by mutation in several cancers such as nonsmall cell lung cancer and cervical carcinomas (Wingo *et al*, 2009; Cancer Genome Atlas Research Network, 2014). LKB1 loss‐of‐function mutations have been reported in cutaneous melanomas (Guldberg *et al*, 1999; Rowan *et al*, 1999), but not in uveal melanomas. Thus, we next interrogated LKB1 expression in primary uveal melanomas from the TCGA cohort and did not find an association with patient survival or metastasis development, likely because the homogeneous expression of LKB1 in this cohort does not reflect its kinase activity. Consistently, LKB1 expression was also homogeneously expressed in liver or skin metastasis (Fig 2A) obtained from a publicly available human metastatic uveal melanoma dataset (Karlsson *et al*, 2020). Immunohistochemistry analyses of human uveal melanoma skin metastases showed intratumoral heterogeneity with either high, or low to no, areas of LKB1 expression (Fig 2B and Appendix Fig S1). When expressed, LKB1 is mainly located in the cytoplasm with, in some regions, a reinforcement of the labeling at the membrane that was previously associated with LKB1 activation (Dogliotti *et al*, 2017). As LKB1 loss favors metastatic uveal melanoma cell proliferation, the low or negative LKB1 areas might mark the active regions of proliferation within the tumors. We also sought to determine whether microenvironmental signals could impact LKB1 activity. We focused our attention on hepatocyte growth factor (HGF) since HGF production has been reported in the eye, skin, and liver, three sites where uveal melanomas can arise and/or metastasize. Our data show that treatment of human OMM1.3 metastatic uveal melanoma cells with HGF enhanced LKB1 phosphorylation on serine 428 which has been associated with its inhibition (Zheng *et al*, 2009; Widjaja *et al*, 2022). HGF also triggered reduced SIK2 phosphorylation on threonine 175, which is phosphorylated and activated by LKB1. Altogether, these findings indicate that HGF can inhibit LKB1 activity (Fig 2C). In line with that, HGF translated into enhanced proliferation of OMM1.3 cells (Fig 2D). Thus, we show that in uveal melanoma, LKB1 can be inactivated by reduced expression or activity.

### LKB1 regulates calcium homeostasis and SLC8A1 expression in metastatic uveal melanoma cells

**Figure 3:**
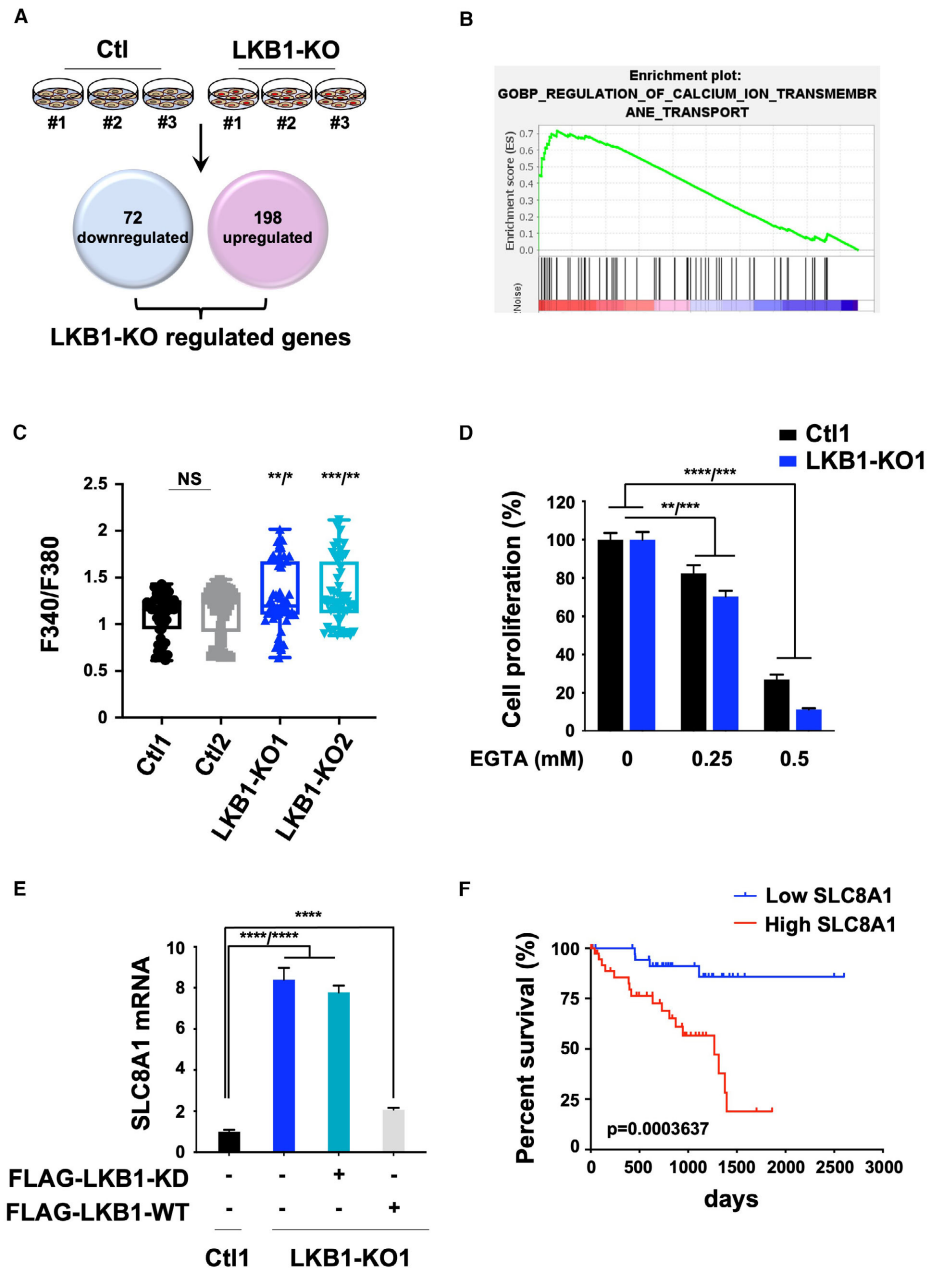
LKB1 loss impact on calcium homeostasis and SLC8A1 expression Schematic of the integrated transcriptional profiling of LKB1 loss in OMM1.3 metastatic uveal melanoma cells. Venn diagrams indicating the number of significant differentially expressed genes selected as follow log2(foldchange) ≥ 1 or ≤ −1 and adjusted *P*‐value ≤ 0.05.GSEA analysis from LKB1‐KO cells versus OMM1.3 Ctl cells, data queried against the “GOBP” group of gene sets.Representative box and whisker plots of transient cytosolic Ca^2+^ changes in two OMM1.3 Ctl and two LKB1‐KO clones. Intracellular calcium concentrations are presented as ratio of the fluorescence signals obtained at 340 and 380 nm (F340/F380). One‐way ANOVA test was performed for comparison between groups. The central band denotes the median value, box contains interquartile ranges, while whiskers mark minimum and maximum values. All technical replicates are shown (*n* = 53). NS, not significant, *P* = 0.8571, **P = 0.0035/*P = 0.0399, ***P = 0.0003/**P = 0.0060. All points are represented.Effect of reducing extracellular calcium with EGTA on Ctl1 and LKB1‐KO1. Indicated cells were seeded at the same density and cultured for 7 days in the presence of DMSO or EGTA (0.25 or 0.5 mM). Relative cell proliferation (percentage). Data represent mean ± SD of three biological replicates (unpaired *t*‐test with Welsch's correction); **P = 0.0057/***P = 0.0008, ****P = 0.000015/***P = 0.0004.RT‐qPCR analysis of SLC8A1 mRNA level in OMM1.3 Ctl1 and LKB1‐KO1 noninfected or infected with a vector encoding FLAG‐tagged kinase‐dead (FLAG‐LKB1‐KD) or wild type (FLAG‐LKB1‐WT) LKB1. Data represent mean ± SD of four biological replicates (unpaired *t*‐test with Welsch's correction); ****P = 0.000047/****P = 0.000024 and ****P = 0.000012.Disease‐specific survival stratified by SLC8A1 mRNA expression (median) from UM‐TCGA dataset (tumors n = 80). Low SLC8A1 mRNA level in blue and high SLC8A1 mRNA level in red. *P*‐value, log‐rank test. Source data are available online for this figure.

To our knowledge, the role of LKB1 in uveal melanoma has not been studied. To gain insights into the mechanisms by which LKB1 restricts metastatic uveal melanoma cell growth, we profiled the transcriptomes of Ctl and LKB1‐KO cells in biological replicates. We identified genes that were specifically upregulated (*n* = 198) and downregulated (*n* = 72) in LKB1‐KO compared to Ctl cells (Fig 3A). Gene set enrichment analysis (GSEA) uncovered four gene sets (of 28 statistically significant) related to calcium (Ca^2+^) in LKB1‐KO cells (Dataset EV2; Fig 3B). These observations indicate that the Ca^2+^ metabolism might play a critical role in LKB1's tumor suppressor function in uveal melanoma cells.

To test this directly, we used FURA‐2‐AM staining and F340/F380 ratio for qualitative description of changes in the cytosolic Ca^2+^ concentration. LKB1‐KO cells exhibited enhanced intracellular Ca^2+^ level (Fig 3C). Moreover, when reducing extracellular free calcium concentration with EGTA, OMM1.3 Ctl1 cell number was strongly reduced, demonstrating that metastatic uveal melanoma cells are addicted to calcium (Fig 3D). This anti‐proliferative response of EGTA treatment was even more pronounced in LKB1‐KO cells, suggesting that the enhanced proliferation of LKB1‐KO OMM1.3 cells relies on exacerbated calcium addiction. We confirmed these findings with BAPTA‐AM, a well‐known membrane permeable chelator of intracellular calcium, that also strongly impaired the proliferative ability of LKB1‐KO cells (Appendix Fig S2). Collectively, these data indicate that the proliferative effect triggered by LKB1 loss might be reliant on Ca^2+^ metabolism. We then interrogated the upregulated genes in LKB1‐KO cells that involved Ca^2+^ metabolism, highlighting the sodium (Na^+^)/calcium (Ca^2+^) exchanger SLC8A1 (solute carrier family 8 member A), which belongs to the NCX family.

**Figure EV2:**
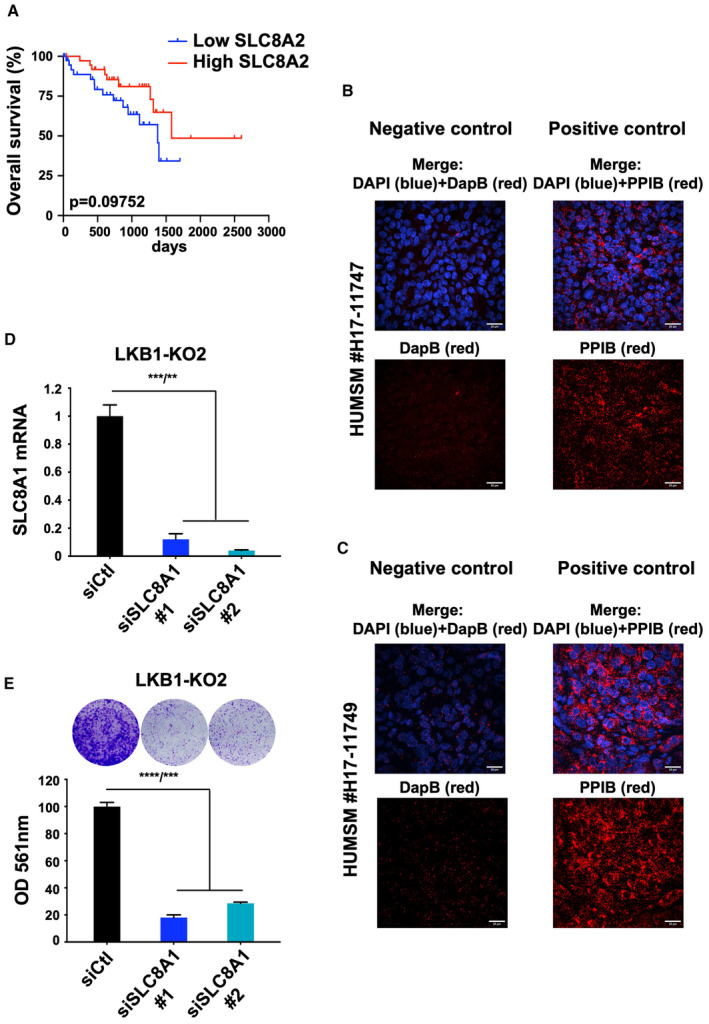
SLC8A1 is required for metastatic uveal melanoma cell proliferation AOverall survival stratified by SLC8A2 mRNA expression (median) from UM‐TCGA dataset (tumors *n* = 80); *P*‐value, log‐rank test.B, CStaining of skin metastasis of human uveal melanomas with human‐positive control probe (PPIB) or with negative control probe (DapB). Scale bars represent 20 μm.DRT–qPCR analysis of SLC8A1 mRNA level in LKB1‐KO2 OMM1.3 cells treated with control siRNA (siCtl) or two different SLC8A1 siRNA (siSLC8A1). Data represent mean ± SD of three biological replicates (unpaired *t*‐test with Welsch's correction); ***P = 0.0005/**P = 0.0023.EColony formation assay of LKB1‐KO2 OMM1.3 cells treated as in (D) grown for 10 days. A total of 75,000 cells were seeded. Representative images and crystal violet quantification at OD 561 nm are shown. Data represent mean ± SD of three biological replicates (unpaired *t*‐test with Welsch's correction); ****P = 0.00001/***P = 0.0002.

To confirm a direct link between SLC8A1 and LKB1, we re‐introduced either a wild‐type or a kinase‐dead version of LKB1 in LKB1‐KO cells and validated that the repression of SLC8A1 was specifically dependent on active LKB1 (Fig 3E). This indicates that SLC8A1 expression is a surrogate of LKB1 activity (Fig 3E). Thus, we interrogated how SLC8A1 expression could predict disease outcomes. It is worth noting that in our transcriptomic data, SLC8A2 levels are very low and not affected by LKB1 loss; nor significant to prognosis in the UM‐TCGA dataset (Fig EV2A), while SLC8A3 is not expressed. In striking contrast, expression of SLC8A1 is significantly associated with worse survival in uveal melanoma patients (Fig 3F).

### SLC8A1 plays a predominant role in metastatic uveal melanoma cell proliferation

**Figure 4:**
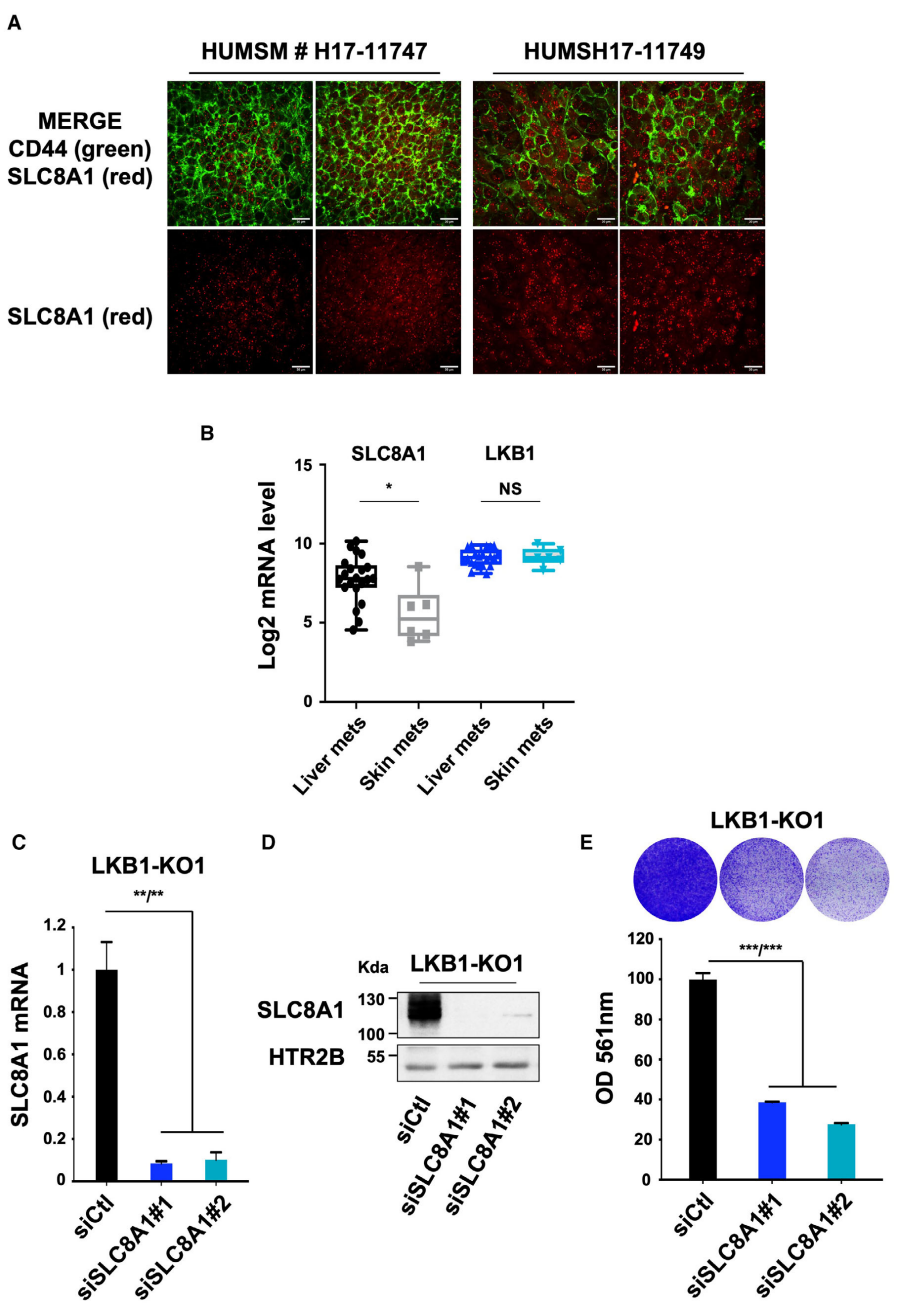
SLC8A1 controls metastatic uveal melanoma cell growth Sections from patients were labeled with the RNAscope probe for SLC8A1 (red). The membrane contours were detected by immunofluorescence using CD44 staining. Images were captured by spinning disk confocal microscopy. Scale bars represent 20 μm.Representative box and whisker plots of Log_2_ fold change mRNA level of SLC8A1 and LKB1 in liver and skin metastasis of human uveal melanomas (Karlsson *et al*, 2020). The central band denotes the median value, box contains interquartile ranges, while whiskers mark minimum and maximum values. Mann–Whitney test was performed for comparison between groups. *P = 0.0151; NS, not significant, *P* = 0.8425. All points are represented.RT–qPCR analysis of SLC8A1 mRNA level in LKB1‐KO1 OMM1.3 cells treated with control siRNA (siCtl) or two different SLC8A1 siRNA (siSLC8A1). Data represent mean ± SD of three biological replicates (unpaired *t*‐test with Welsch's correction); **P = 0.0065/**P = 0.0046.SLC8A1 protein level was studied by immunoblot in membrane‐enriched lysates in the same conditions. HTR2B was used as a loading control. Representative images of three independent experiments are shown.Colony formation assay of LKB1‐KO1 cells grown for 10 days in the same conditions. A total of 75,000 cells were seeded. Representative images of three independent experiments and crystal violet quantification at OD 561 nm are shown. Data represent mean ± SD of three biological replicates (unpaired *t*‐test with Welsch's correction); ***P = 0.0008/***P = 0.0004. Source data are available online for this figure.

To investigate the role of SLC8A1 in uveal melanoma biology, we first analyzed its expression in human metastatic uveal melanoma samples. Given the lack of high‐quality SLC8A1 antibody for immunochemistry, its expression in human skin metastasis was evaluated by RNAscope® fluorescence *in situ* hybridization assay in two different patients combined with immunofluorescence staining of CD44 to detect the membrane contour. The staining was heterogeneous showing cells with both high and low levels of SLC8A1 (Fig 4A). Positive and negative control stainings of these samples are shown in Fig EV2B and C. These findings are in line with the high and low LKB1 protein expression areas observed by immunohistochemistry. In agreement with the expression pattern in primary uveal melanomas, data showed that SLC8A1 expression is heterogeneous in metastatic uveal melanomas (Fig 4A). Consistent with SLC8A1 expression being a surrogate of LKB1 activity rather than LKB1 expression itself, we observed higher SLC8A1 levels in liver metastases than in skin metastases with no significant changes in LKB1 expression (Fig 4B). Altogether, these observations suggest that LKB1 activity is reduced in the liver.

We next evaluated the role of SLC8A1 in the proliferative effect mediated by LKB1 loss. Two different siRNAs, which efficiently reduced SLC8A1 at both mRNA and protein levels (Fig 4C and D), significantly impaired the enhanced colony formation ability induced by LKB1 loss (Fig 4E). The effect of SLC8A1 knockdown by siRNA on reducing metastatic uveal melanoma cell formation capacity was confirmed in a second LKB1‐KO clone (Fig EV2D and E). These results indicate that the upregulation of SLC8A1 expression downstream of LKB1 loss controls, at least in part, the proliferative capacity of metastatic uveal melanoma cells.

### SIK2 is a hub‐promoting SLC8A1‐dependent proliferation in LKB1‐deficient metastatic uveal melanoma

**Figure 5:**
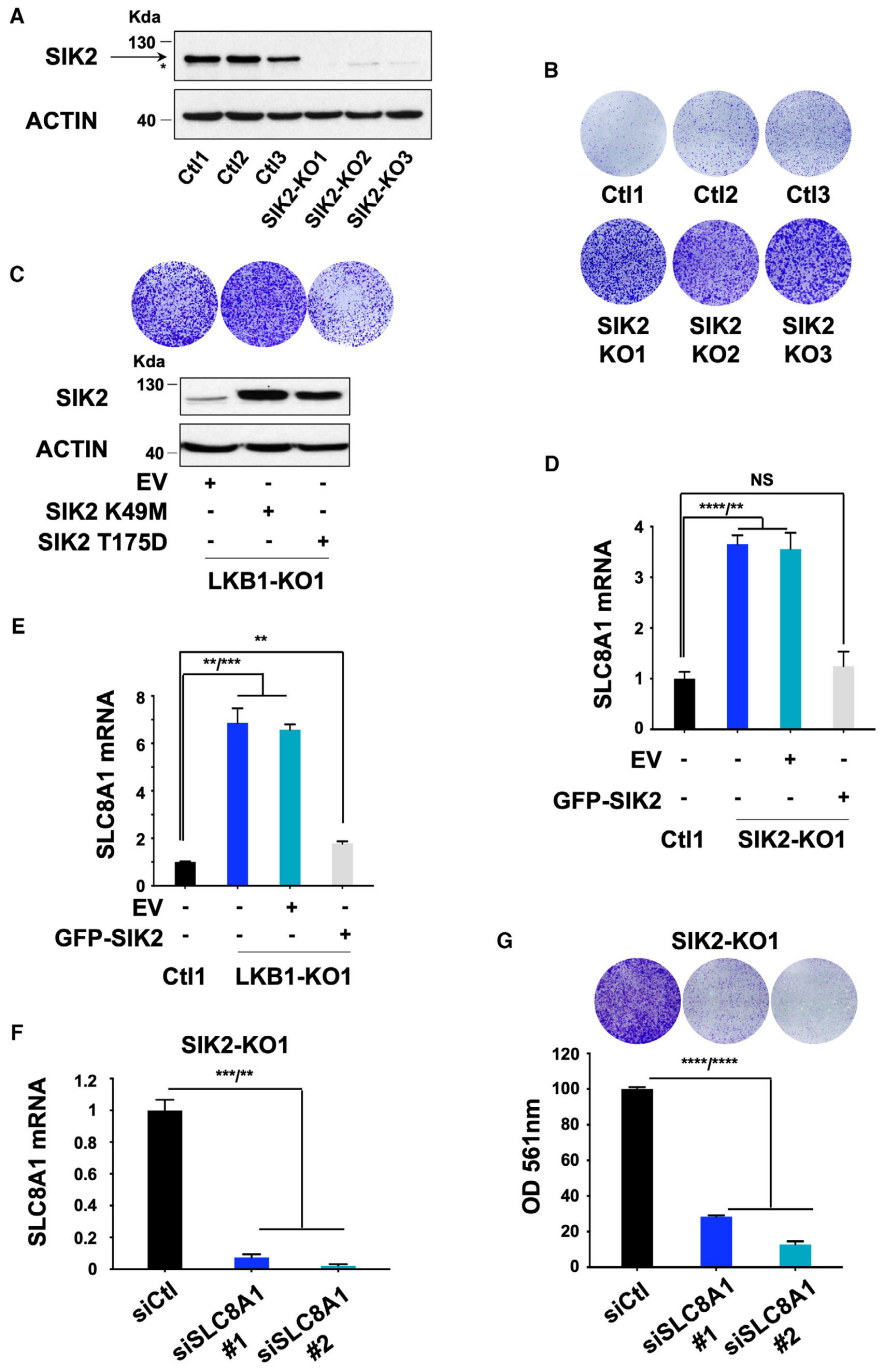
SIK2 loss is critically required for LKB1 loss‐driven metastatic uveal melanoma cell proliferation Immunoblot of SIK2 in the indicated whole‐cell lysates of OMM1.3 Ctl or SIK2‐KO cells. Three different OMM1.3 Ctl and SIK2‐KO cell lines are shown. B‐actin was used as a loading control.Colony formation assay of OMM1.3 Ctl and SIK2‐KO cells grown for 10 days. Cells were seeded at low density. Representative images of three independent experiments and crystal violet quantification at OD 561 nm are shown.LKB1‐KO1 cells were transfected with an empty vector (EV) or vectors encoding either a kinase‐dead (SIK2 K49M) or a constitutively active (SIK2 T175D) form of SIK2. Immunoblot (bottom) of SIK2 for the indicated cell lines and colony formation assay (top) are shown. B‐actin was used as a loading control. Representative images of three independent experiments are shown.RT–qPCR analysis of SLC8A1 mRNA level in OMM1.3 Ctl1 and SIK2‐KO1 noninfected (left) or infected with an empty vector (EV) or a vector‐encoding SIK2‐WT. Data represent mean ± SD of three biological replicates (unpaired *t*‐test with Welsch's correction); ****P = 0.000057/**P = 0.0018; NS, not significant, *P* = 0.2765.RT–qPCR analysis of SLC8A1 mRNA level in OMM1.3 Ctl1 and LKB1‐KO1 noninfected (left) or infected with an empty vector (EV) or a vector‐encoding SIK2‐WT. Data represent mean ± SD of three biological replicates (unpaired *t*‐test with Welsch's correction); **P = 0.0036/***P = 0.0005, **P = 0.0040.RT–qPCR analysis of SLC8A1 mRNA level in OMM1.3 SIK2‐KO1 cells treated with control siRNA (siCtl) or two different SLC8A1 siRNA (siSLC8A1). Data represent mean ± SD of three biological replicates (unpaired *t*‐test with Welsch's correction); ***P = 0.0008/**P = 0.0013.Colony formation assay of OMM1.3 SIK2‐KO1 cells grown for 10 days in the same conditions. A total of 75,000 cells were seeded. Representative images of three independent experiments and crystal violet quantification at OD 561 nm are shown. Data represent mean ± SD of three biological replicates (unpaired *t*‐test with Welsch's correction); ****P = 0.00000085/****P = 0.000002. Source data are available online for this figure.

**Figure EV3:**
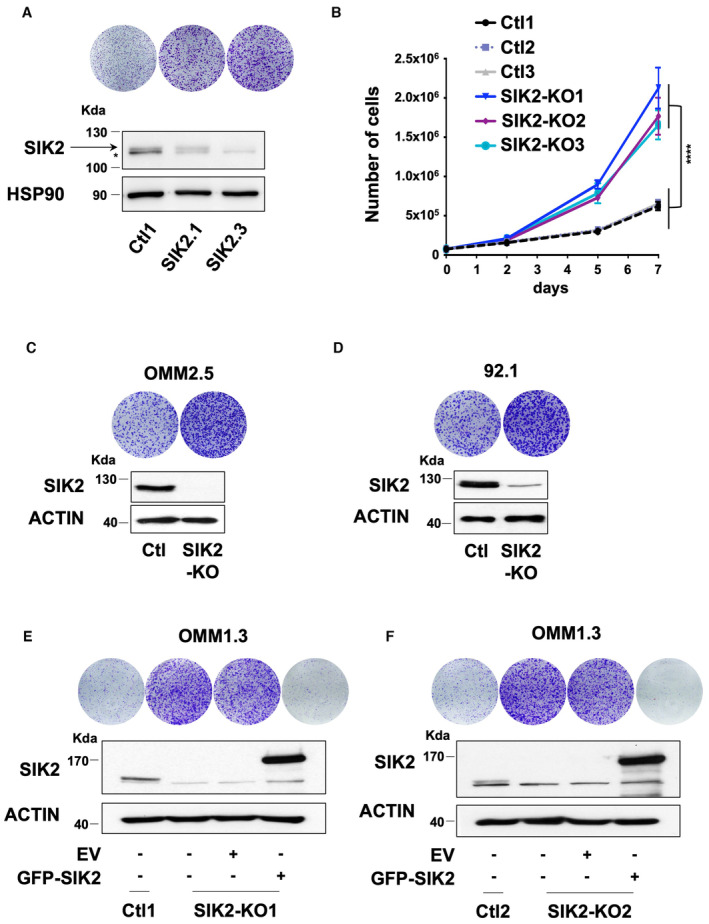
SIK2 deletion causes metastatic uveal melanoma cell proliferation A(Bottom) Immunoblot of SIK2 in the indicated whole‐cell lysates of OMM1.3‐pooled SIK2‐CRISPR cell lines. HSP90 was used as a loading control. (Top) Colony formation assay of OMM1.3 Ctl1 and pooled SIK2‐KD or KO cells grown for 10 days. Cells were seeded at low density. Representative images of three independent experiments are shown.BProliferation curves of OMM1.3 Ctl and SIK2‐KO cells. Data represent mean ± SD of three biological replicates (unpaired *t*‐test with Welsch's correction); ****P = 0.000041.C, D(Bottom) SIK2‐CRISPR‐KO was generated in human OMM2.5 metastatic and human 92.1 primary uveal melanoma cells. Representative western blot assay of SIK2 is shown. B‐actin was used as a loading control. (Top) Colony formation assay of control cells (Ctl) and pooled SIK2‐KO cells grown for 10 days. Cells were seeded at low density. Representative images of three independent experiments are shown. Ctl cells from Fig EV1C and E have been used.E, FOMM1.3 Ctl1, SIK2‐KO1 cells and OMM1.3 Ctl2, SIK2‐KO2 cells were noninfected (left) or infected with an empty vector (EV) or a vector‐encoding SIK2‐WT. Colony formation assay and immunoblot of SIK2 are shown. B‐actin was used as a loading control. Representative images of three independent experiments are shown.

LKB1 is known to regulate the activity of a wide range of downstream kinases, such AMPK, SIK, BRSK, and MARK, and different isoforms within these families. Interestingly, while sgRNAs targeting SIK2 were enriched in our CRISPR/Cas9 kinome screen, sgRNAs for other LKB1 downstream targets were not (Dataset EV1), thereby suggesting that in uveal melanoma, LKB1 operates through SIK2 regulation. To rule out involvement of AMPK, the most studied downstream LKB1 effector, we first assessed LKB1 KO effect on AMPK activity. Western blot experiments revealed no change in phosphorylation of AMPK, mTOR, and S6 in LKB1‐KO versus control cells (Appendix Fig S3). Together, these observations raise the hypothesis that SIK2 is the main direct downstream target of LKB1 involved in the regulation of SLC8A1. We first validated the functional impact of SIK2 loss by introducing individual sgRNAs in pooled OMM1.3 cells and observed an increased cell colony‐forming capacity (Fig EV3A). These findings were confirmed in SIK2‐KO clonal‐derived OMM1.3 cell lines (SIK2‐KO1, SIK2‐KO2, and SIK2‐KO3) (Fig 5A), in which SIK2 deficiency resulted in an increased colony‐forming capacity (Fig 5B) and growth rate (Fig EV3B). Validation was performed in both OMM2.5 and 92.1 cells, showing enhanced colony‐forming ability upon SIK2 depletion (Fig EV3C and D). Importantly, the hyperproliferative phenotype caused by SIK2 loss was rescued when SIK2‐WT was reintroduced into SIK2‐KO clonal cell lines (SIK2‐KO1 and SIK2‐KO2) compared to noninfected cells or introduction of an empty vector (Fig EV3E and F).

**Figure EV4:**
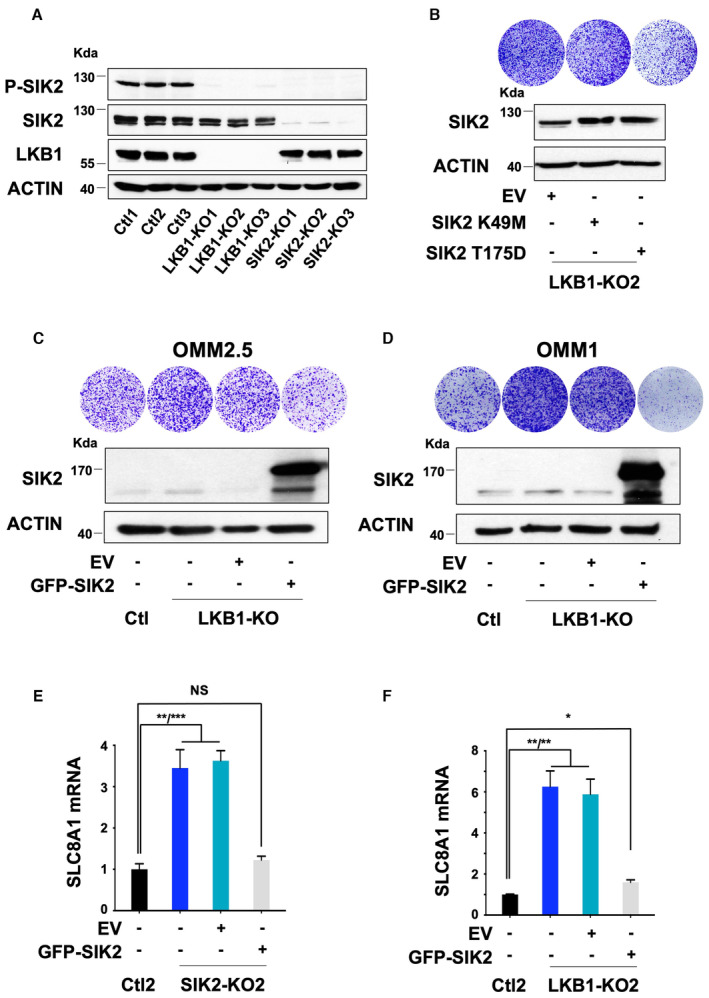
SIK2 is regulating metastatic uveal melanoma cell proliferation downstream LKB1 AImmunoblot of SIK2, phospho‐SIK2, and LKB1 in the indicated whole‐cell lysates from control, LKB1‐KO, and SIK2‐KO OMM1.3 cells. B‐actin was used as a loading control. Representative images of three independent experiments are shown.BLKB1‐KO2 cells transfected with an empty vector (EV) or vectors encoding either a kinase‐dead (SIK2 K49M) or a constitutively active (SIK2 T175D) form of SIK2. Immunoblot for SIK2 and colony formation assay are shown. B‐actin was used as a loading control. Representative images of three independent experiments are shown.C, DHuman OMM2.5 and OMM1 metastatic uveal melanoma cells Ctl or LKB1‐KO cells were noninfected (left) or LKB1‐KO cells were infected with an empty vector (EV) or a vector‐encoding SIK2‐WT. Colony formation assay and immunoblot of SIK2 are shown. B‐actin was used as a loading control. Representative images of three independent experiments are shown.ERT–qPCR analysis of SLC8A1 mRNA level in OMM1.3 Ctl2 and SIK2‐KO2 noninfected (left) or infected with an empty vector (EV) or a vector‐encoding SIK2‐WT. Data represent mean ± SD of three biological replicates (unpaired *t*‐test with Welsch's correction); **P = 0.0068/***P = 0.004; NS, not significant, *P* = 0.0844.FRT–qPCR analysis of SLC8A1 mRNA level in OMM1.3 Ctl2 and LKB1‐KO2 noninfected (left) or infected with an empty vector (EV) or a vector‐encoding SIK2‐WT. Data represent mean ± SD of three biological replicates (unpaired *t*‐test with Welsch's correction) **P = 0.0069/**P = 0076 and *P = 0.0109.

Next, we inquired whether SIK2 phosphorylation by LKB1 was involved in restraining proliferation. Our data demonstrate that LKB1‐KO dramatically reduced SIK2 phosphorylation to an extent similar to SIK2‐KO (Fig EV4A). Adding back a constitutively active form of SIK2 (SIK2 T175D), but not a kinase‐dead form (SIK2 K49M), alleviated the proliferation phenotype mediated by LKB1 suppression in two different clones (LKB1‐KO1 and LKB1‐KO2) (Figs 5C and EV4B). We showed in additional cellular models including human metastatic uveal melanoma cells (OMM2.5 and OMM1) and primary uveal melanoma cells (92.1 and Mel270) that adding back wild‐type SIK2 alleviated the hyperproliferative phenotype mediated by LKB1 loss (Fig EV4C and D and Appendix Fig S4). These findings demonstrate and support that SIK2 and its kinase activity are required for LKB1 function in constraining uveal melanoma cell proliferation.

In the following, we assessed the effect of SIK2 loss on SLC8A1 expression. Our data showed that SLC8A1 expression was enhanced in SIK2‐KO cells, while it returned to basal levels upon adding back SIK2 WT (Fig 5D). Likewise, the increase in SLC8A1 mediated by LKB1 loss was dramatically reduced by SIK2 WT forced expression (Fig 5E). These observations were confirmed in additional SIK2‐KO and LKB1‐KO clones (Fig EV4E and F). Finally, we also showed that SIK2‐KO cells treated with SLC8A1 siRNA proved to be less proliferative compared to cells treated with control siRNA (Fig 5F and G). These results demonstrate that SIK2 is a culprit of SLC8A1 regulation in the absence of LKB1 and of the constrained proliferation downstream of LKB1 in uveal melanoma cells.

### LKB1 and SIK2 suppression are associated with enhanced mitochondrial Ca^2+^ and ROS levels

**Figure 6:**
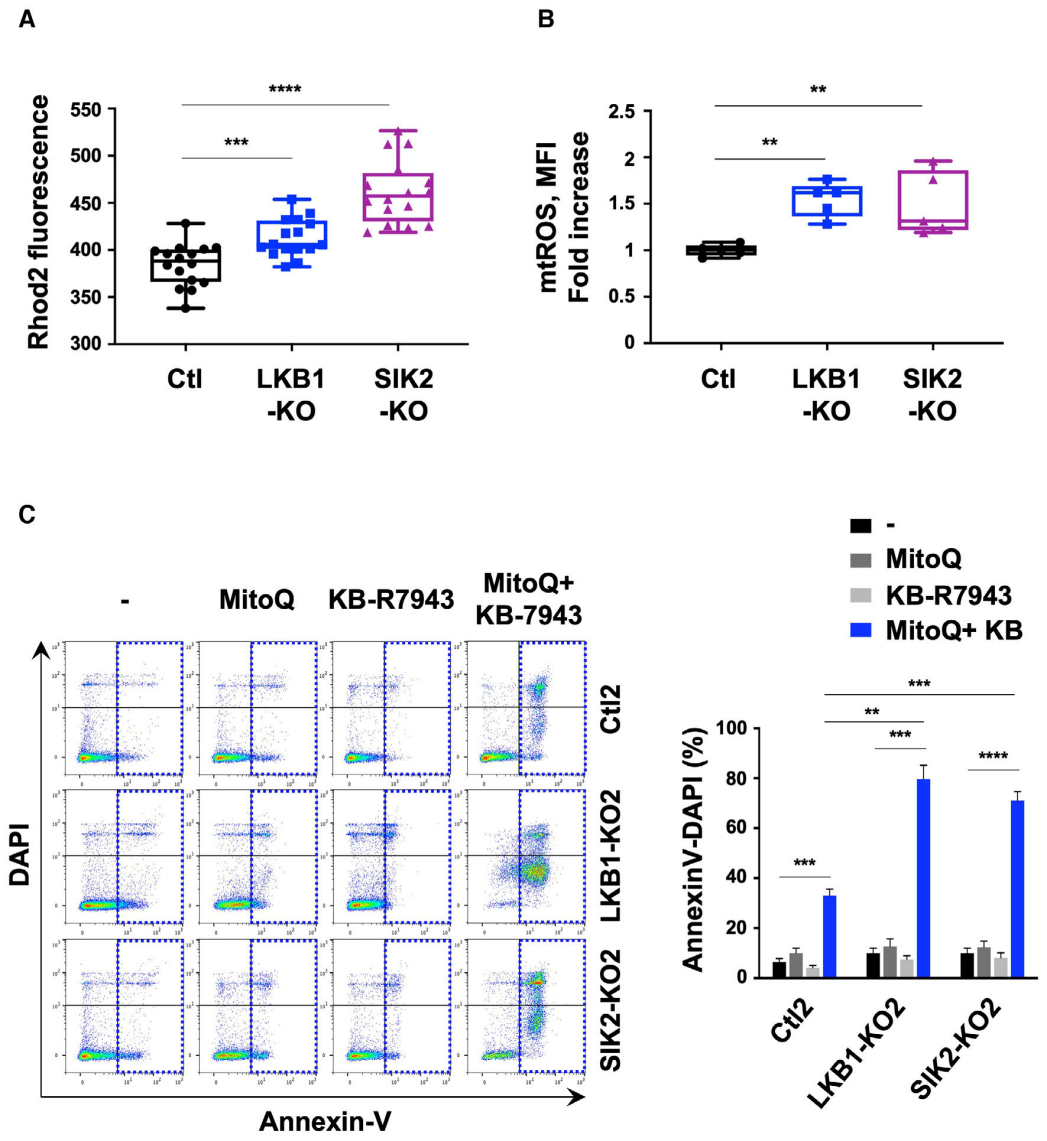
LKB1 and SIK2 deficiency impacts mitochondrial metabolism and metastatic uveal melanoma cell survival Representative box and whisker plots of Rhod2 fluorescence in control, LKB1‐KO, and SIK2‐KO OMM1.3 cells. The central band denotes the median value, box contains interquartile ranges, while whiskers mark minimum and maximum values. All technical replicates are shown (*n* = 16); Mann–Whitney test was performed for comparison between groups. ***P = 0.0007 and ****P = 0.00000004. All points are represented.Representative box and whisker plots of mitochondrial ROS levels in control, LKB1‐KO, and SIK2‐KO OMM1.3 cells measured using a dihydrorhodamine 123 staining. The central band denotes the median value, box contains interquartile ranges, while whiskers mark minimum and maximum values. All biological replicates are shown (*n* = 5); Mann–Whitney test was performed for comparison between groups. **P = 0.0079. All points are represented.Analysis of apoptosis in OMM1.3 cells (control, LKB1, and SIK2‐KO) treated with 200 nM MitoQ and/or 5 μM KB‐R7943 for 72 h. Annexin V diagram (left) and quantitation of the percentage of apoptotic cells using the Annexin V assay (right). Data represent mean ± SD of three biological replicates (unpaired *t*‐test with Welsch's correction); ***P = 0.0004, ***P = 0.0007, ****P = 0.000098, **P = 0.0012, and ***P = 0.0002. Source data are available online for this figure.

Mitochondria can act as a spatial Ca^2+^ buffer in many cells but the link to calcium addiction in cancer cells is poorly understood. We therefore measured mitochondrial Ca^2+^ (mtCa^2+^) level using the sensitive dye Rhod‐2 AM. Data revealed more Ca^2+^ taken up by mitochondria in LKB1‐KO and SIK2‐KO cells compared to the control cells (Fig 6A). High mtCa^2+^ level stimulates respiratory chain activity leading to higher amounts of mitochondrial reactive oxygen species (mtROS) (Görlach *et al*, 2015), which can activate intracellular signaling pathways and contribute to proliferation and survival in many cancers (Sabharwal & Schumacker, 2014). To determine mtROS, we used dihydrorhodamine 123‐based assays. Enhanced mtROS level was detected in LKB1‐KO and SIK2‐KO uveal melanoma cells compared to the control cells (Fig 6B). We next asked whether mtROS could be exploited to inhibit the growth of uveal melanoma cells. We evaluated the effect of a mitochondria‐targeted antioxidant, mitoquinol (MitoQ), which has been used in clinical trials to selectively deactivate mitochondrial superoxide (NCT04851288). MitoQ alone did not affect WT, or LKB1‐ and SIK2‐deficient cell survival (Fig 6C, left). We next reasoned that the mitochondrial calcium addiction‐driving proliferation may promote alternative pathways. Thus, we used the SLC8A1 inhibitor KB‐R7943 alone or in combination with MitoQ. The combination of these treatments promoted a significant increase in apoptosis in LKB1‐KO and SIK2‐KO cells than control cells (Fig 6C, right). Together, SLC8A1 and mitochondrial ROS mediate uveal melanoma cell survival and their co‐targeting may represent a new therapeutic option.

### Preclinical implications of the LKB1‐SIK2‐SLC8A1 cascade

Based on the complementary role of SLC8A1 and mtROS in promoting LKB1‐deficient uveal melanoma cell proliferation, we next investigated the therapeutic relevance of these pathways on tumor growth *in vivo*. Subcutaneous injections of control and LKB1‐KO cells were performed in athymic nude mice. When the tumors, formed with LKB1‐KO cells, reached 100 mm^3^, they were treated with KB‐R7943, MitoQ, or their combination and vehicle. Of note, control cells did not grow as xenografts within the time frame of the experiment.

**Figure 7:**
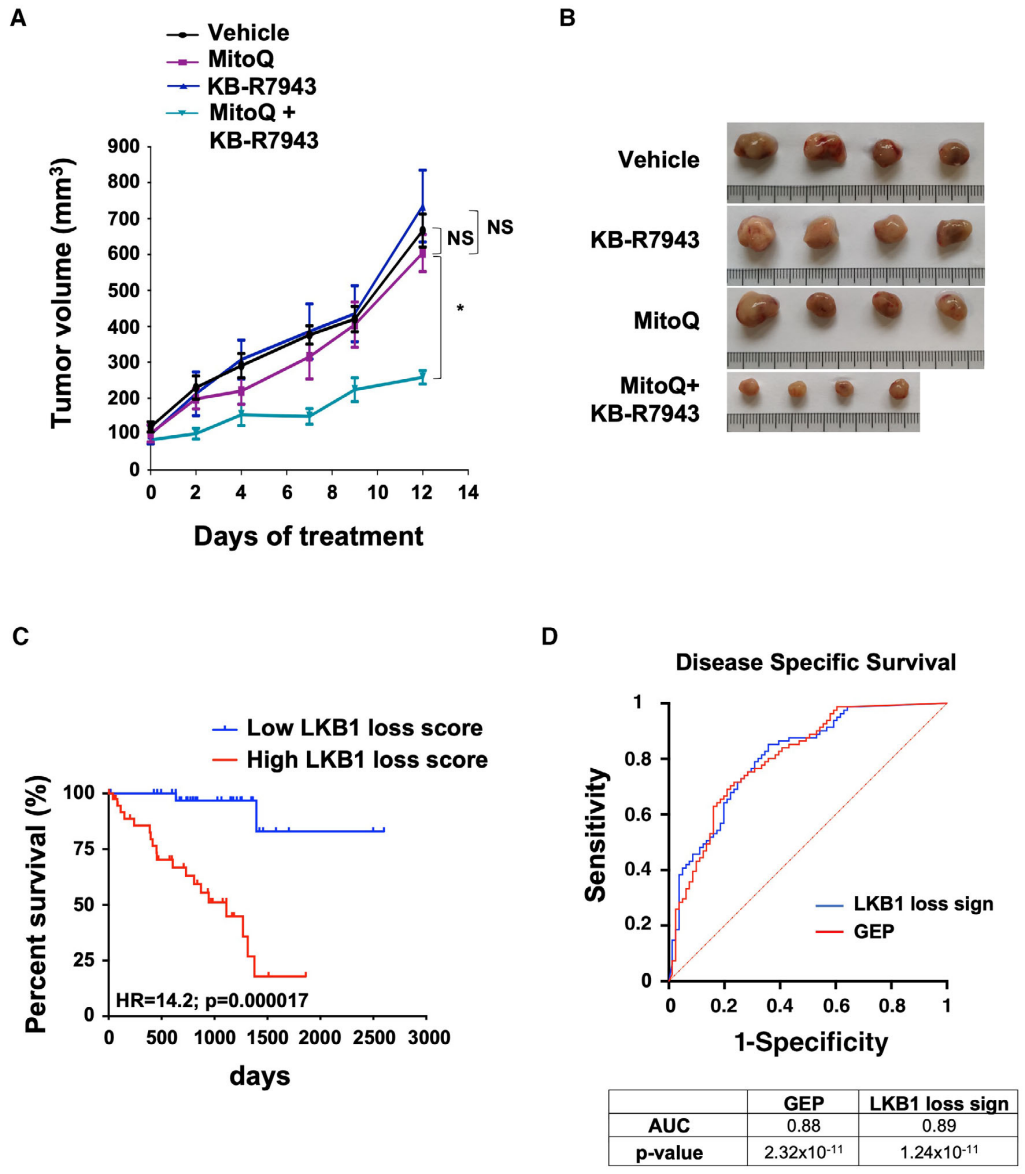
Inhibition of SLC8A1 and mtROS decreases malignant phenotypes in LKB1‐KO OMM1.3 cells *in vivo* Quantification of tumor volume in nude mice‐bearing xenograft tumors of LKB1‐KO cells treated with MitoQ (5 mg/kg), KB‐R7943 (5 mg/kg), a combination of both, or vehicle three times per week (*n* = 4 mice, each group). Mann–Whitney test was performed for comparison between groups. Data are mean ± SEM. *P = 0.0286; NS, not significant, *P* = 0.6857Representative images of tumors are shown.Kaplan–Meier analysis of the LKB1‐KO signature in UM‐TCGA dataset (tumors *n* = 80).Time‐dependent receiver operating characteristic (ROC) curves show the sensitivity and specificity of our LKB1 signature compared to the gene expression profiling signature (GEP) (Onken *et al*, 2004) for predicting the patient disease‐specific survival (UM‐TCGA cohort). Source data are available online for this figure.

**Figure EV5:**
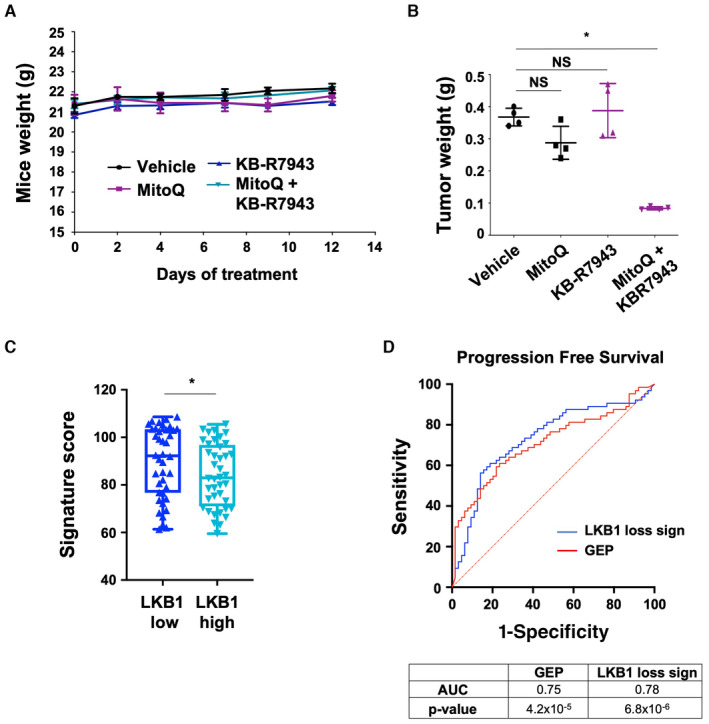
Inhibition of SLC8A1 and mitochondrial ROS trigger tumor regression ABody weights of mice during treatment are shown as the mean ± SEM (*n* = 4 mice, each group).BTumor weights of the indicated xenografts at the endpoint (12 days) are shown as the mean ± SD (*n* = 4 mice, each group). Mann–Whitney test was performed for comparison between groups. *P = 0.0286; NS, not significant, *P* = 0.1143.CRepresentative box and whiskers plots of the LKB1 loss signature score based on LKB1 mRNA expression level (low and high) from the UM‐TCGA dataset. Mann–Whitney test was performed for comparison between groups. *P = 0.0487. All points are represented.DTime‐dependent receiver operating characteristic (ROC) curves show the sensitivity and specificity of our LKB1 signature compared to the gene expression profiling signature (GEP) (Onken *et al*, 2004), for predicting the progression‐free survival (Laurent *et al*, 2011a; Data ref: Laurent *et al*, 2011b).

Note that the treatment did not affect body weight (Fig EV5A). A strong reduction in tumor volume, size, and weight was observed in athymic mice bearing the LKB1‐deficient cells treated with the combination of KB‐R7943 and MitoQ (Figs 7A and B, and EV5B). These findings further support the concept that calcium metabolism and mtROS are intertwined in supporting uveal melanoma progression *in vivo*.

### Identification of a clinical score that is predictive of the prognosis and response to SLC8A1 inhibitor and mitoquinol combination

To further test the clinical relevance of our findings, we developed an 11‐gene signature associated with LKB1 depletion from the RNA‐seq analysis (Table 1) to test and identify tumors with reduced LKB1 activity and/or expression level, and to predict the risk of metastasis in patients with uveal melanoma.

**Table 1:** LKB1 loss gene signature list along with Log_2_ fold change and adjusted *P*‐value from our transcriptomic data (LKB1 KO cells vs. control cells).

Gene name	Log_2_(FC)LKB1 KO	Adj *P*‐value
ADCY1	5.844413545	1.26006E‐77
SLC8A1	2.852637522	1.03953E‐14
PTPRH	2.487422366	3.06709E‐07
ECM1	2.455083114	3.80959E‐14
SH3PXD2B	2.372422178	6.76335E‐10
NECAB2	2.082100057	5.34412E‐05
ABCB4	1.92412242	3.97457E‐06
JAG1	1.530538254	4.0881E‐05
ST3GAL1	1.287585826	0.000154854
TNFRSF19	1.174432349	7.1963E‐05
SLC16A6	1.029611523	0.004884716

Using the TCGA cohort, we first compared LKB1 expression levels with our gene signature. Low LKB1 expression is correlated with high level of our gene signature and high LKB1 expression is correlated with low level of our gene signature (Fig EV5C). In addition to identifying low/high LKB1 expression in tumors, our signature might also provide an evaluation of LKB1 activity. Moreover, high LKB1 loss signature score is associated with reduced survival (Fig 7C) and with high sensitivity and specificity to identify patients who will develop metastasis and eventually die as shown by the ROC curve (Fig 7D). Time‐dependent *ROC* analyses indicated that this signature performed as well, or even slightly better than the gene expression profiling (GEP) signature used to predict the risk of metastasis in patients with uveal melanoma (Onken *et al*, 2004). Prognosis value of this signature was validated in another cohort (Laurent *et al*, 2011a; Data ref: Laurent *et al*, 2011b) (Fig EV5D). Therefore, the LKB1 loss gene signature might serve as a novel stratification tool for improving patient prognosis. Additionally, as KB‐R7943 and MitoQ treatment is more efficient in LKB1‐deficient uveal melanoma cells, our LKB1‐loss signature might also be predictive of the response to this therapeutic combination.

## Discussion

In this work, using a kinome‐wide CRISPR screen, we identified LKB1, as a kinase whose loss of function is critically required for uveal melanoma cell proliferation. Although genetic alterations in LKB1 have not been reported in uveal melanomas, nongenetic mechanisms could trigger decreased expression and/or activity. In line with this, our immunochemistry data showed a variable LKB1 expression in human uveal melanoma samples. Moreover, in those expressing LKB1, we observed either a membrane or cytoplasmic labeling that indicates an active or an inactive LKB1, respectively. How LKB1 activity can be modulated? We showed that HGF, which has been reported to contribute to uveal melanoma progression and which is expressed in skin and liver, two major sites of uveal melanoma metastasis, reduced activity of the LKB1‐SIK2 module. Of course, LKB1 activity can be regulated by other growth factors or metabolic conditions that remain to be disclosed.

LKB1 is a master kinase that activates AMPK and AMPK‐like proteins. Although the best‐studied substrate of LKB1 is AMPK (Shackelford & Shaw, 2009), our data showed that phosphorylation of AMPK, or its downstream effectors mTOR and S6 are not affected by LKB1 loss.

Moreover, we found that AMPK knockdown by siRNA reduced uveal melanoma cell growth (Appendix Fig S5). This is reminiscent of a previous study showing similar effect of AMPK inhibition in uveal melanoma cells (Chua *et al*, 2022) and suggesting that AMPK inhibition does not promote uveal cell proliferation.

One can argue that in culture conditions with high serum concentration, the effects of LKB1 KO on mTOR phosphorylation is masked. However, in these same conditions, LKB1 KO is able to promote uveal melanoma cell proliferation, indicating that the pro‐proliferative effect of LKB1 KO is not mediated through mTOR activation.

Here, we identified SIK2 as the pivotal downstream effector of LKB1. Indeed, adding back SIK2 in LKB1 KO cells almost entirely abrogated the ability of LKB1 loss to induce a hyperproliferative phenotype. Therefore, our data show that SIK2 is critically required downstream LKB1 to constrain uveal melanoma cell proliferation. Likewise, previous studies reported that LKB1's effect is mediated by non‐AMPK kinase such as the SIK family of kinases in lung adenocarcinoma cell lines (Pierce *et al*, 2021).

At the molecular level, we identified a novel mechanism linking LKB1 and SIK2 loss to enhanced expression of a unique SLC8A1‐dependent calcium transport system favoring intracellular Ca^2+^ level. Calcium is relevant to a variety of important processes in tumor progression such as proliferation and survival. These findings identified a novel mechanism of calcium addiction in uveal melanomas. However, how LKB1 and SIK2 loss affects SLC8A1 levels and/or activity remains to be determined. A few reports indicate that SLC8A1 activity is regulated by different factors, such as pH, ATP, phosphatidylinositol 4,5‐bisphosphate (PIP2), and post‐translational modifications including phosphorylation or palmitoylation (Schulze *et al*, 2003; Morad *et al*, 2011; Plain *et al*, 2017). These factors might perturb the structure of the transporter or the macromolecular complex in which it is functioning and impact its membrane anchorage.

Our findings were unexpected since the sodium/calcium exchanger of the SLC8A family generally transports Ca^2+^ ions out of the cell in exchange for sodium ions and is considered one of the most important cellular mechanisms for removing Ca^2+^. However, recent studies have called into question the reverse mode of this transporter, promoting Ca^2+^ influx, in pathological settings such as in diverse types of cancers (Sennoune *et al*, 2015; Rodrigues *et al*, 2019; Chovancova *et al*, 2020). Our data extend these previous findings by showing that LKB1 loss increases SLC8A1 expression and intracellular Ca^2+^ concentration in uveal melanoma. Consequently, we detected more mitochondrial Ca^2+^ in LKB1‐ and SIK2‐deficient cells. Ca^2+^ stimulates mitochondrial oxidative metabolism which are hallmark of tumors by playing an important role in ATP production, to meet the cells' energy demands. These results provide the basis for potential anti‐cancer therapeutic strategies targeting cellular metabolism.

Indeed, our data also show that LKB1‐ or SIK2‐deficient uveal melanoma cells are more sensitive and vulnerable to disruption of Ca^2+^ homeostasis and oxidative stress. This metabolic vulnerability provides a therapeutic strategy to treat these metastatic uveal melanoma subtypes. In fact, here we demonstrated *in vitro* and in a xenograft model that LKB1 loss increases uveal melanoma cells' sensitivity to the combination of the SLC8A1 inhibitor, KB‐R7943 with MitoQ, the only mitochondrial antioxidant that has safely been used in clinical trials (Smith & Murphy, 2010; Rossman *et al*, 2018). As we have identified a molecular signature for low LKB1 expression/activity and shown that LKB1 loss increases uveal melanoma cell sensitivity to the combination KB‐R7943 with MitoQ, this signature might therefore predict response to this treatment.

In summary, our data demonstrate that LKB1 or SIK2 deficiency increases SLC8A1 expression and creates a hypersensitivity to SLC8A1 inhibition and ROS blockers. This reveals a promising therapeutic perspective for a subset of metastatic uveal melanomas with low LKB1 activity that can be identified by a molecular prognostic and predictive signature disclosed in this work.

## Material and Methods

### Cell cultures

Human metastatic uveal melanoma cell lines OMM1.3 (GNAQ^Q209P^) (Chen *et al*, 1997) and OMM2.5 (GNAQ^Q209P^) (Chen *et al*, 1997) isolated from liver and human primary uveal melanoma cells 92.1 (GNAQ^Q209L^) (De Waard‐Siebinga *et al*, 1995) and Mel270 (GNAQ^Q209P^) (Chen *et al*, 1997) were all grown in RPMI supplemented with 10% FBS at 37°C in a humidified atmosphere containing 5% CO_2_ (Griewank *et al*, 2012). Human metastatic uveal melanoma cell line OMM1 isolated from skin was grown as previously described (Luyten *et al*, 1996).

Cell lines are regularly tested for mycoplasma and are mycoplasma free, and were authenticated through short tandem repeat (STR) profiling.

### Biochemicals

KB‐R7943 (HY‐15415 MedchemTronica), EDTA (E5134), and EGTA (E4378) were obtained from Sigma and MitoQ (*in vitro*: 89950 Cayman Chemical, *in vivo*: HY‐100116A MedchemTronica).

### Pooled CRISPR library details

In total, 3,052 unique sgRNAs targeting 763 human kinome genes for four guides per target were used for pooled CRISPR screens (Addgene #75312). Libraries were amplified following the Broad Institute protocol. To ensure library diversity, colonies were collected from 15 bacterial plates after transformation of STBL4 electrocompetent cells (New England Biolabs). The pool of plasmids was prepared for infection using Qiafilter plasmid midi kit (12243, Qiagen).

### CRISPR‐Cas9 screen

Human OMM1.3 uveal melanoma cells were first infected with the lentiCas9‐Hygro (LCH) (Addgene # 104995) and selected with 10 μg/ml hygromycin (10687010, Life Technologies). Cells were then infected with the sgRNA library at a low MOI (< 1) to ensure a single sgRNA vector per cell. After 48 h of infection, cells were treated with 0.5 μg of puromycin (P8833, Sigma) for 72 h and < 20% of cells were selected, corresponding to single vector copy. Cells were next expanded for 10 days. A fraction of cells were collected at day 0 to ensure a proper coverage of sgRNAs. Medium was changed every 3 days. At day 35, cells from all conditions were collected and genomic DNA was extracted. Since melanin pigment may interfere with DNA‐ and/or RNA‐based molecular profiling (Lagonigro *et al*, 2004), we purified the samples using the OneStep^TM^ PCR inhibitor Removal Kit (ZD6030, Zymo Research). The integrated sgRNAs were then amplified by PCR with primers containing multiplexing barcodes and adaptors and sequenced on the Illumina NextSeq500. Hits were selected based on the log_2_ fold change of sgRNA reads at day 35. Analyses and plots of the sequencing data were conducted using Prism 6 software (GraphPad Software) and rank products analysis to determine *P*‐values. Data were analyzed using the software Mageck which calculates a score based on a fold change where either sgRNA is depleted or enriched compared to the control condition.

### RNA sequencing and gene signature

Reads were preprocessed to remove adapter and low‐quality sequences (Phred quality score below 20). After this preprocessing, reads shorter than 40 bases were discarded for further analysis. These preprocessing steps were performed using cutadapt version 1.10. Reads were mapped to rRNA sequences using bowtie version 2.2.8 and reads mapping to rRNA sequences were removed for further analysis. Reads were mapped onto the hg38 assembly of *Homo sapiens* genome using STAR version 2.5.3a. Gene expression quantification was performed from uniquely aligned reads using htseq‐count version 0.6.1p1, with annotations from Ensembl version 99 and “union” mode. Only nonambiguously assigned reads have been retained for further analyses. Read counts have been normalized across samples with the median‐of‐ratios method (Anders & Wolfgang, 2010). Differential gene expression analysis was performed using the methodology implemented in the Bioconductor package DESeq2 version 1.16.1 (Love *et al*, 2014). *P*‐values were adjusted for multiple testing by the method proposed by Benjamini and Hochberg (Benjamini & Hochberg, 1995). Deregulated genes were defined as genes with log2(foldchange) ≥ 1 or ≤ −1 and adjusted *P*‐value ≤ 0.05. To generate the gene signature associated with LKB1 depletion, differentially expressed genes were selected as follows: log_2_(foldchange) ≥ 1 and adjusted *P*‐value ≤ 0.05. After sorting by adjusted *P*‐value, the top 100 protein‐coding genes were selected. Then, genes were filtered out based on their expression level within the TCGA‐UM cohort. The top 10 genes upregulated after LKB1 loss from our transcriptomic data ranked by adjusted *P*‐value show a high prognosis value (ROC AUC = 0.85, *P* < 0.0001). However, to improve the prognosis value of the signature, the retained genes were then explored by using Cox regression model to calculate the hazard ratio (HR) leading to the identification of an 11‐gene signature, with an even higher prognosis value (ROC AUC = 0.89, *P* < 0.0001) (Fig 7D).

### Transient transfection of siRNA

Briefly, a single pulse of 50 nM of control or SLC8A1 (J‐007620‐08‐0010, Dharmacon and SASI_Hs02_00325535, Sigma) or AMPK siRNA (sc‐29673, Sigma) was administered to the cells at 50% confluency through transfection with 5 μl of Lipofectamine™ RNAiMAX (13778–150, Life Technologies) in Opti‐MEM medium as described (Leclerc *et al*, 2019).

### Vectors

Vectors encoding LKB1 wild‐type (#8592) or kinase‐dead mutant (#8593) were purchased from Addgene. Vectors encoding SIK2 wild‐type, the constitutively active mutant (T175D), or the kinase‐dead mutant (K49M) were previously described (Tang *et al*, 2013).

### mRNA preparation and real‐time/quantitative PCR

The mRNAs were prepared using TRIzol (15596026, Fisher Scientific) according to a standard procedure. QRT–PCR was performed using SYBR® Green I (4368708, Fisher Scientific) and Multiscribe Reverse Transcriptase (4311235, Applied Biosystems) and subsequently monitored using the StepOnePlus Real‐Time PCR Systems (Applied Biosystems, Foster City, CA). The detection of the ACTIN gene was used to normalize the results. Primer sequences for each cDNA were designed using Primer bank software. (ACTIN: 5′‐GCTGTGCTACGTCGCCCTG‐3′, 5′‐GGAGGAGCTGGAAGCAGCC‐3′; SLC8A1: 5′‐AAAGAGGAAGAGGAGAGGCG‐3′, 5′‐CAAGGGCCAGGTTTGTCTTC‐3′) (https://pga.mgh.harvard.edu/primerbank/).

### Western blot assays

Briefly, cell lysates (30 μg) were separated using SDS–PAGE, transferred onto a PVDF membrane, and subsequently exposed to the appropriate antibodies, anti‐SLC8A1 (ab177952, 1/1,000), and anti‐pan phospho‐threonine SIK (ab199474, 1/1,000), from Abcam; anti‐LKB1 (D60C5; 1/1,000), anti‐SIK2 (D28G3; 1/1,000), anti‐mTOR (7C10; 1/1,000), anti‐phospho‐mTOR (#2971; 1/1,000), anti‐S6 (5G10; 1/1,000), anti‐phospho‐S6 (#4858; 1/1,000), anti‐AMPK (#2532; 1/1,000), and anti‐phospho‐AMPK (40H9; 1/1,000) from CST; and anti‐actin (sc‐47778, 1/1,000) and anti‐HSP90 (sc‐13119, 1/1,000) from Santa Cruz Biotechnology. The proteins were visualized using the ECL system (Amersham). Detection of SLC8A1 was conducted after membrane enrichment using the Mem‐PER™ Plus Membrane Protein Extraction Kit (89842, Thermo Fisher Scientific). The western blots shown are representative of at least three independent experiments.

### Colony formation assay

Human uveal melanoma cells were seeded onto six‐well plates at low density (2,500 cells/well for Mel270, 5,000 cells/well for 92.1, 7,500 cells/well for OMM1 and OMM2.5, and 10,000 cells/wells for OMM1.3), allowed to adhere overnight, and cultured as indicated. Then, the colonies were stained with 0.04% crystal violet/2% ethanol in PBS for 30 min. Photographs of the stained colonies were captured. Crystal violet was then solubilized, and growth was monitored by measuring the absorbance at 561 nm. Photographs of the stained colonies were captured. The colony formation assay was performed in triplicate.

### Immunohistochemistry and RNAscope staining

For immunohistochemical stainings, the cool immunohistochemistry machine was used. Dako Target Retrieval Solution pH9 (Enzo Life Sciences ADI‐950‐280‐0015) was used for all stainings. LKB1 (sc‐374334, Santa Cruz Biotechnology) was used. mRNAs for *SLC8A1* in sections from human metastatic uveal melanomas were detected with RNAscope assay (Biotechne) according to the manufacturer's protocols. Images were captured with a spinning disk confocal microscope (Nikon).

Uveal melanoma metastases were from the University Research Priority Program biobank Zurich, University Hospital Zurich, Switzerland. The experiments were conducted in conformity with the principles set out in the WMA Declaration of Helsinki and the Department of Health and Human Services Belmont Report. All patients included in this study have signed a patient release form, which has been approved by an ethics committee and assigned the numbers EK647 and EK800.

### Intracellular Ca^2+^ measurements

Cells were plated in 96‐well plates at 20,000 cells per well 24 h before the experiment. Adherent cells were loaded for 45 min at 37°C with the ratiometric dye Fura2‐AM (65‐0858‐39, Thermo Fisher Scientific) at 5 μM, then washed by PBS solution supplemented with 2 mM Ca^2+^ (Sigma). During the experiment, cells were incubated with physiologic saline solution (PSS) Ca^2+^. Fluorescence emission was measured at 510 nm using the FlexStation‐3 (Molecular Devices, San José, CA, USA) with excitation at 340 and 380 nm.

### Mitochondrial Ca^2+^ and ROS measurements

To measure mitochondrial Ca^2+^ using Rhod‐2 AM (543 nm/580–650 nm) dye (R1245MP, Invitrogen), the cells were cultured at 50–60% confluency. The cells were washed with media without FBS and antibiotic–antimycotic agents. Then, the cells were incubated in media containing 3 μM Rhod‐2 AM (without FBS and antibiotic–antimycotic agents) at 37°C for 45 min. The cells were washed and kept in PSS (HEPES‐buffered saline solution [140 mM NaCl, 1.13 mM MgCl_2_, 4.7 mM KCl, 2 mM CaCl_2_, 10 mM D‐glucose, and 10 mM HEPES, adjusted to pH 7.4 with NaOH]) containing 2 mM CaCl_2_ for imaging. Mitochondrial ROS were measured after cell incubation in a FACS buffer (PBS 1×, 1% BSA, and 2 mM EDTA) containing 5 μM dihydrorhodamine123 (D23806, Invitrogen) for 30 min. The cells were washed and kept in FACS buffer.

### Animal experimentation

Animal experiments were performed in accordance with French law and approved by a local institutional ethical committee (APAFIS #36147‐2022031316569590 v5). The animals were maintained on a 12 h light/dark cycle in a temperature‐controlled facility at 22°C and provided free access to food (standard laboratory chow diet). Human OMM1.3 LKB1‐WT and LKB1‐KO melanoma cells (3 × 10^6^ cells) were subcutaneously inoculated into 8‐week‐old female immune‐deficient athymic nude FOXN1nu mice 6‐week‐old (Janvier Laboratory). Based on the literature, different treatment regimens of intraperitoneal injection of KB‐R7943 have been used (Xu *et al*, 2009; Long *et al*, 2016). For MitoQ, different treatment regimens of intraperitoneal injection were also published (Mao *et al*, 2013; Bao *et al*, 2023). To cover the different protocols published for both drugs, we chose injection three times per week. Preliminary experiments using 5 or 10 mg/kg of KB‐R7943 showed that intraperitoneal injection of 10 mg/kg KB‐R7943 three times per week had a small effect on mouse body weight. Given that our experiment, based on the *in vitro* data, aimed to study the combination of KB‐R7943 and MitoQ, we chose to reduce KB‐R7943 dosage to 5 mg/kg and to use similar dosage for MitoQ. Thus, when the tumors reached 100 mm3, KB‐R7943 (5 mg/kg), MitoQ (5 mg/kg), a combination of both, or vehicle (0.5% methylcellulose + 0.1% Tween‐80 molecular‐grade sterile water) was administered three times per week for up to 12 days by intraperitoneal injection. The growth tumor curves were determined after measuring the tumor volume using the equation V = (L × W2)/2 as previously reported (Ohanna *et al*, 2018). Mice were randomly assigned to the different treatment groups.

### Statistics

No data were excluded from the analyses. Investigators were not blinded. No statistical methods were used to determine the sample size. Sample size was determined to be adequate based on the magnitude and consistency of measurable differences between groups. Statistical significance between groups was determined as indicated in the legends.

## Author contributions

**Thomas Strub:** Conceptualization; data curation; formal analysis; supervision; funding acquisition; writing – original draft; writing – review and editing. **Sarah Proteau:** Data curation; formal analysis; investigation. **Imène Krossa:** Investigation. **Chrystel Husser:** Investigation. **Maxime Guéguinou:** Conceptualization; data curation; formal analysis; investigation. **Federica Sella:** Data curation; formal analysis; investigation; methodology. **Karine Bille:** Investigation. **Marie Irondelle:** Software; methodology. **Mélanie Dalmasso:** Investigation. **Thibault Barouillet:** Investigation. **Yann Cheli:** Investigation. **Céline Pisibon:** Investigation. **Nicole Arrighi:** Investigation. **Sacha Nahon‐Estève:** Resources. **Arnaud Martel:** Resources. **Lauris Gastaud:** Resources. **Sandra Lassalle:** Methodology. **Olivier Mignen:** editing. **Patrick Brest:** editing. **Nathalie M Mazure:** editing. **Frédéric Bost:** editing. **Stéphanie Baillif:** Resources. **Solange Landreville:** Resources. **Simon Turcotte:** Resources. **Dan Hasson:** Formal analysis. **Saul Carcamo:** Formal analysis. **Christophe Vandier:** Conceptualization. **Emily Bernstein:** editing. **Laurent Yvan‐Charvet:** Conceptualization; editing. **Mitchell P Levesque:** Resources; methodology. **Robert Ballotti:** Conceptualization; data curation; formal analysis; funding acquisition; writing – original draft; writing – review and editing. **Corine Bertolotto:** Conceptualization; data curation; formal analysis; supervision; funding acquisition; writing – original draft; writing – review and editing.

## Disclosure and competing interests statement

The authors declare that they have no conflict of interest.

## Supporting information



Appendix S1

Expanded View Figures PDF

Dataset EV1

Dataset EV2

PDF+

Source Data for Figure 1

Source Data for Figure 2

Source Data for Figure 3

Source Data for Figure 4

Source Data for Figure 5

Source Data for Figure 6

Source Data for Figure 7

## Data Availability

The RNA‐sequencing data generated and/or analyzed during this study have been deposited in the NCBI Gene Expression Omnibus (GEO) database (https://www.ncbi.nlm.nih.gov/geo/) under the SuperSerie GSE233458 (http://www.ncbi.nlm.nih.gov/geo/query/acc.cgi?acc=GSE233458).
