# Publisher Correction: LKB1-SIK2 loss drives uveal melanoma proliferation and hypersensitivity to SLC8A1 and ROS inhibition

**DOI:** 10.1038/s44321-024-00114-1

**Published:** 2024-08-20

**Authors:** Sarah Proteau, Imène Krossa, Chrystel Husser, Maxime Guéguinou, Federica Sella, Karine Bille, Marie Irondelle, Mélanie Dalmasso, Thibault Barouillet, Yann Cheli, Céline Pisibon, Nicole Arrighi, Sacha Nahon-Estève, Arnaud Martel, Lauris Gastaud, Sandra Lassalle, Olivier Mignen, Patrick Brest, Nathalie M Mazure, Frédéric Bost, Stéphanie Baillif, Solange Landreville, Simon Turcotte, Dan Hasson, Saul Carcamo, Christophe Vandier, Emily Bernstein, Laurent Yvan-Charvet, Mitchell P Levesque, Robert Ballotti, Corine Bertolotto, Thomas Strub

**Affiliations:** 1 University Côte d’Azur, Nice, France; 2 Inserm, Biology and Pathologies of melanocytes, team1, Equipe labellisée Ligue 2020, and Equipe labellisée ARC 2022, Mediterranean Centre for Molecular Medicine, Nice, France; 3 University of Tours, Tours, France; 4 Inserm, N2C UMR 1069, Tours, France; 5 Department of Dermatology, University Hospital Zurich, University of Zurich, Zurich, Switzerland; 6 Inserm, Hematometabolism and metainflammation, team 13, Mediterranean Centre for Molecular Medicine, Nice, France; 7 Centre Hospitalier Universitaire of Nice, Department of Ophthalmology, Nice, France; 8 Centre Antoine Lacassagne, Nice, France; 9 Laboratory of Clinical and Experimental Pathology, University Hospital of Nice, FHU OncoAge, Cote d’Azur University, Biobank BB-0033-00025, IRCAN team 4, OncoAge FHU, Nice, France; 10 LBAI, UMR1227, Univ Brest, Inserm, Brest, France; 11 IRCAN team 4, Inserm, CNRS, FHU-oncoAge, IHU-RESPIRera, Nice, France; 12 Inserm, Cancer, Metabolism and environment, team, Equipe labellisée Ligue 2022, Mediterranean Centre for Molecular Medicine, Nice, France; 13 Département d’ophtalmologie et d’ORL-CCF, Faculté de médecine, Université Laval, Quebec City, QC Canada; 14 CUO-Recherche and Axe médecine régénératrice, Centre de recherche du CHU de Québec-Université Laval, Quebec City, QC Canada; 15 Centre de recherche sur le cancer de l’Université Laval, Quebec City, QC Canada; 16 Centre de recherche en organogénèse expérimentale de l’Université Laval/LOEX, Quebec City, QC Canada; 17 Cancer Axis, Centre de recherche du Centre Hospitalier de l’Université de Montréal/Institut du cancer de Montréal, Montréal, QC Canada; 18 Hepato-Pancreato-Biliary Surgery and Liver Transplantation Service, Centre hospitalier de l’Université de Montréal, Montréal, QC Canada; 19 Department of Oncological Sciences, Tisch Cancer Institute, Icahn School of Medicine at Mount Sinai, New York, NY 10029 USA; 20 Tisch Cancer Institute Bioinformatics for Next Generation Sequencing (BiNGS) Facility, Icahn School of Medicine at Mount Sinai, New York, NY USA

## Abstract

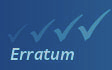

**Correction to:** *EMBO Mol Med* (2023) 15:e17719. 10.15252/emmm.202317719 | Published online 15 November 2023

During the publication proofing process, the author identified several formatting errors. These errors were not corrected by the publisher prior to the release of the publication. These errors are now corrected.

**Figure 7D is corrected.**

**Figure EV5D is corrected.**

**Figure:**
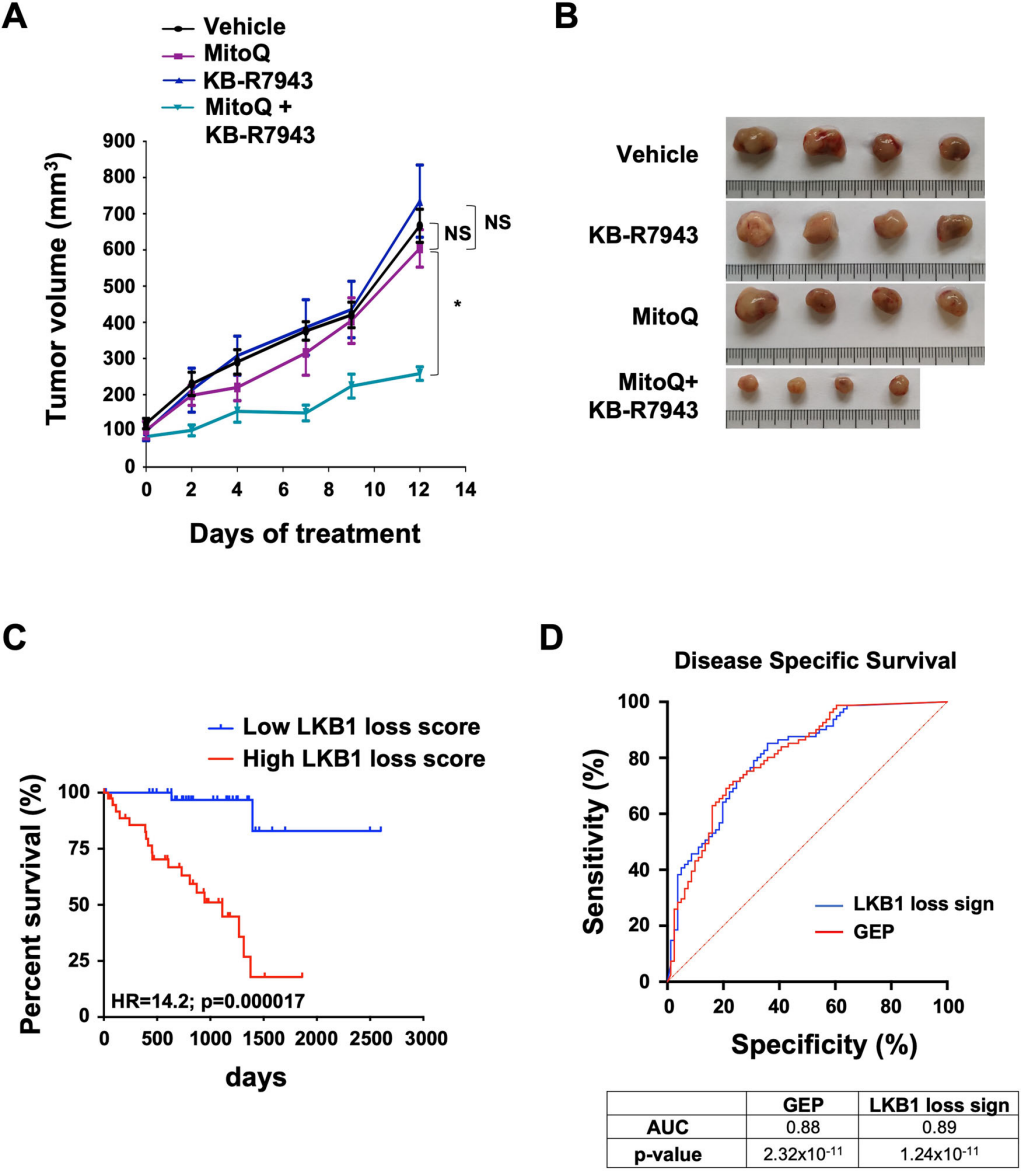
Figure 7. Original.

**Figure:**
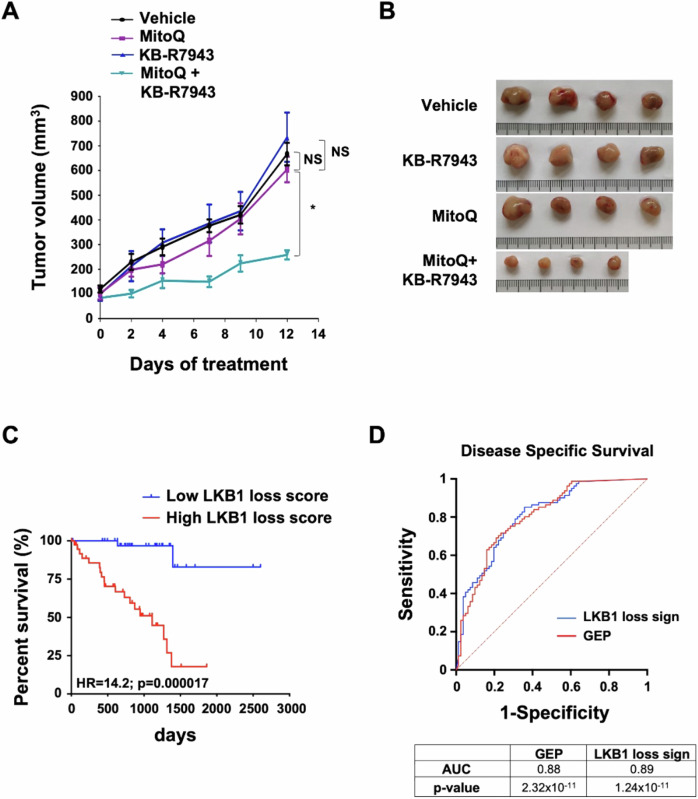
Figure 7. Corrected.

**Figure:**
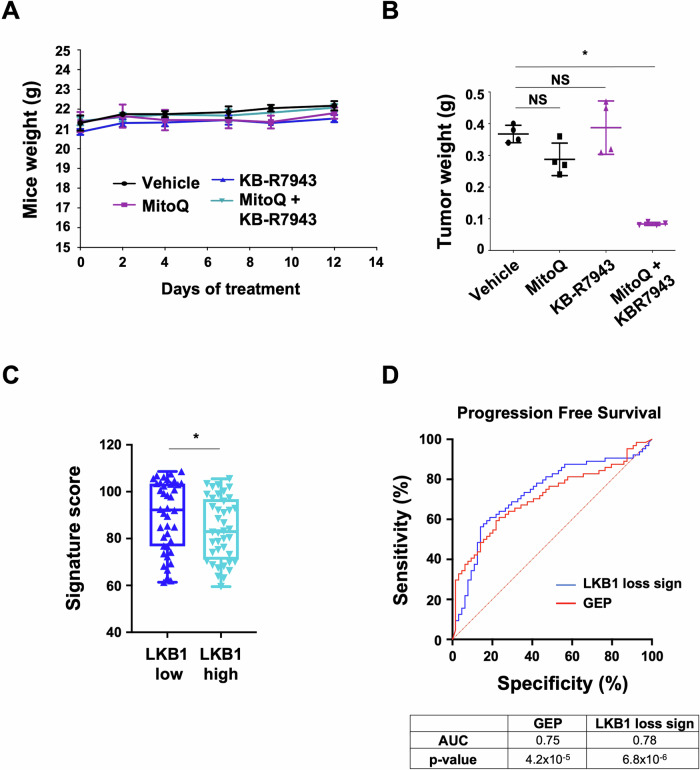
Figure EV5. Original.

**Figure:**
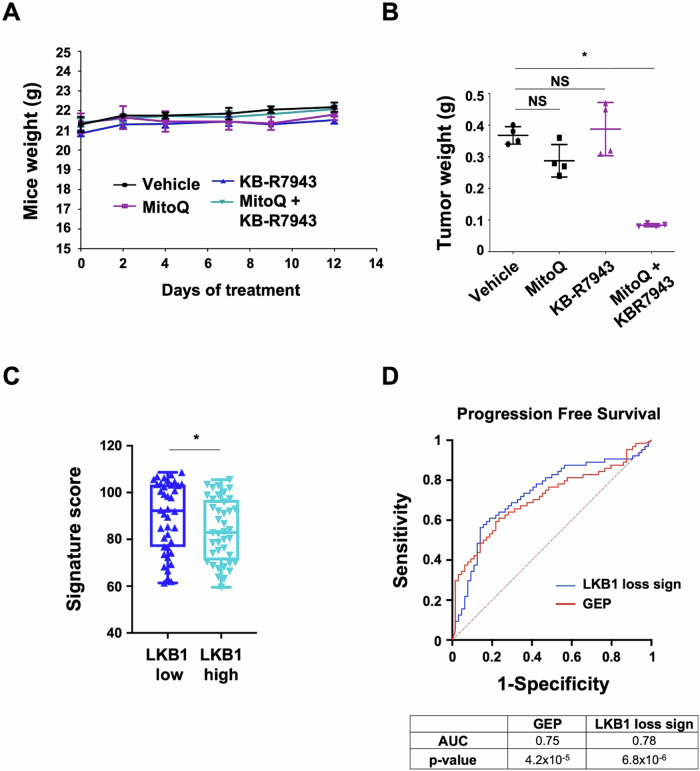
Figure EV5. Corrected.

**The Acknowledgments section is corrected**.

The Acknowledgments section is corrected from:

The wild-type and mutant SIK2 expression vectors were kindly provided by Dr. Dong-Yan Jin (The University of Hong Kong, Hong Kong). This work was supported by the French government, INSERM, La Ligue Nationale contre le cancer, INCA PLBio to C. B. (INCA-16070), Labex Signalife and La Ville de Nice. SP is fellowship from La Ligue Nationale contre le cancer. SL is a Junior 2 Research Scholar of the Fonds de recherche du Québec-Santé (FRQS). ST was supported by the FRQS Young Clinician Scientist Seed Grant, the FRQS Clinician Scientist Junior 1&2 Salary Award, the Institut du Cancer de Montréal Establishment Award, and the Université de Montréal Roger Des Groseillers Research Chair in Hepatopancreatobiliary Surgical Oncology. The procurement of human UM tissues was possible thanks to the Quebec Uveal Melanoma Biobank (funded by the Vision Health Research Network, a thematic network supported by the FRQS), and the Centre hospitalier de l’Université de Montréal hepatopancreatobiliary cancer biobank and prospective registry. The authors thank the following persons for patient recruitment, biospecimen acquisition and handling, and prospective maintenance of clinicopathological data: Dr. C. Bellerive and S. Marcoux (Clinique des tumeurs oculaires du CHU de Québec-Université Laval), as well as L. Rousseau, S. Langevin and J. Bilodeau (CHUM hepatopancreatobiliary cancer and prospective registry). I.K is a former fellowship from Région Provence-Alpes-Côte d’Azur “Emplois jeunes doctorants”. T.S acknowledges the financial support provided by La Fondation pour la Recherche Médicale (FRM ARF201809006989) and the Fondation de France (00120250/WB-2021-33281). The authors thank the C3M imaging facility (INSERM U1065) supported by IBiSA, the C3M genomic facility (GenoC3M), le conseil Régional and la cancéropole PACA. The authors thank Nicolas Nottet for analyses of sequencing data using the MAGeCK method. The authors thank Celine Keim (IGBMC, Strasbourg) for analyses of sequencing data.

To: (changes in bold)

The wild-type and mutant SIK2 expression vectors were kindly provided by Dr. Dong-Yan Jin (The University of Hong Kong, Hong Kong). This work was supported by the French government, INSERM, La Ligue Nationale contre le cancer, INCA PLBio to C.B. (INCA-16070), Labex Signalife and La Ville de Nice. S.P. is fellowship from La Ligue Nationale contre le cancer. S.L. is a Junior 2 Research Scholar of the Fonds de recherche du Québec-Santé (FRQS). ST was supported by the FRQS Young Clinician Scientist Seed Grant, the FRQS Clinician Scientist Junior 1&2 Salary Award, the Institut du Cancer de Montréal Establishment Award, and the Université de Montréal Roger Des Groseillers Research Chair in Hepatopancreatobiliary Surgical Oncology. The procurement of human UM tissues was possible thanks to the Quebec Uveal Melanoma Biobank (funded by the Vision Health Research Network, a thematic network supported by the FRQS), and the Centre hospitalier de l’Université de Montréal hepatopancreatobiliary cancer biobank and prospective registry. The authors thank the following persons for patient recruitment, biospecimen acquisition and handling, and prospective maintenance of clinicopathological data: Dr. C. Bellerive and S. Marcoux (Clinique des tumeurs oculaires du CHU de Québec-Université Laval), as well as L. Rousseau, S. Langevin and J. Bilodeau (CHUM hepatopancreatobiliary cancer and prospective registry). I.K. is a former fellowship from Région Provence-Alpes-Côte d’Azur “Emplois jeunes doctorants”. T.S. acknowledges the financial support provided by La Fondation pour la Recherche Médicale (FRM ARF201809006989) and the Fondation de France (00120250/WB-2021-33281). The authors thank the C3M imaging facility (INSERM U1065) supported by IBiSA, the C3M genomic facility (GenoC3M), le conseil Régional and la cancéropole PACA. The authors thank Nicolas Nottet for analyses of sequencing data using the MAGeCK method. The authors thank Celine Keim (IGBMC, Strasbourg) for analyses of sequencing data. **C.B. and S.L. thank The France Canada Research Fund**.

