# Supplementary material for: LKB1‐SIK2 loss drives uveal melanoma proliferation and hypersensitivity to SLC8A1 and ROS inhibition
Source: EMBO Mol Med. 2023 Nov 15;15(12):e17719. doi: 10.15252/emmm.202317719 (PMC10701601; doi:10.15252/emmm.202317719)
Supplement: Supplementary file 1 — Appendix S1 [file EMMM-15-e17719-s010.pdf]

Table of contents

Appendix Figure S1. Heterogeneous expression of LKB1 in human metastatic uveal melanoma

Appendix Figure S2. Role of calcium in uveal melanoma cell proliferation

Appendix Figure S3. mTOR signalling in uveal melanoma

Appendix Figure S4. SIK2 is regulating metastatic uveal melanoma cell proliferation downstream LKB1

Appendix Figure S5. AMPK knockdown in uveal melanoma

Proteau_ Appendix Figure S1

HUMSM #H21-2981

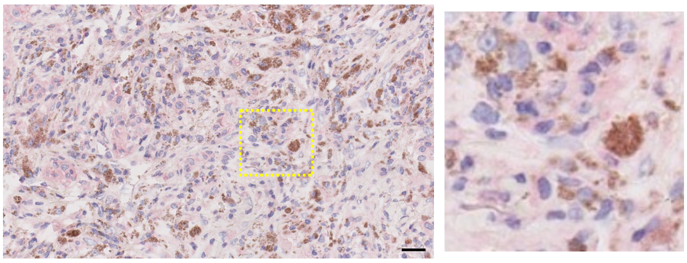

HUMSM #H21-14991

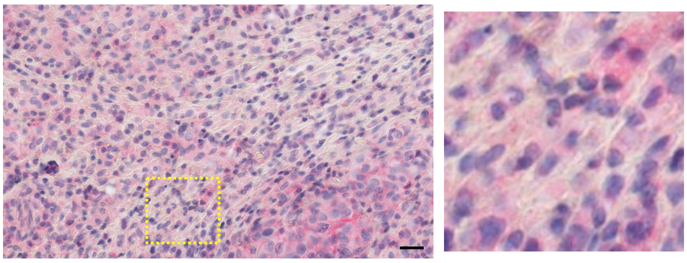

HUMSM # H16-10540

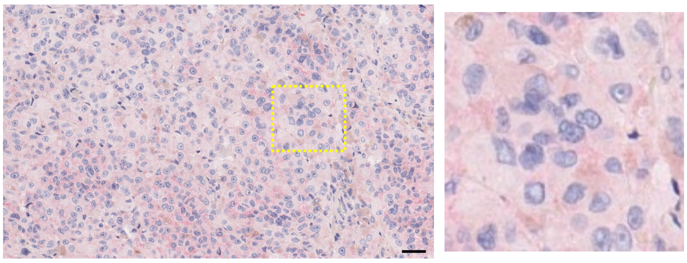

HUMSM #H20-8372

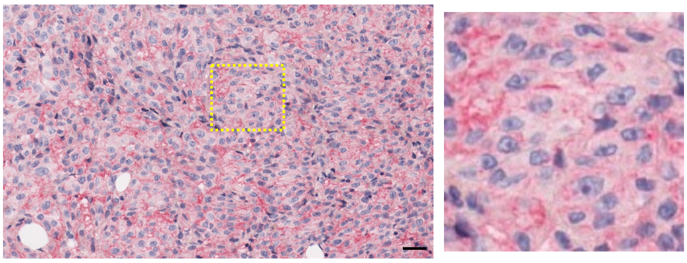

HUMSM #H17-11747

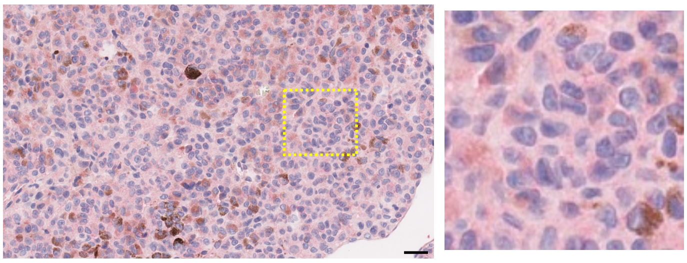

HUMSM # H17-16621

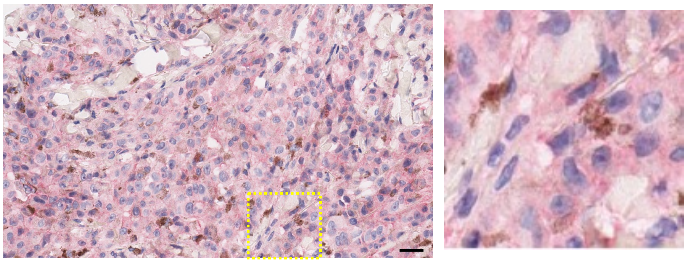

HUMSM #H17-12566

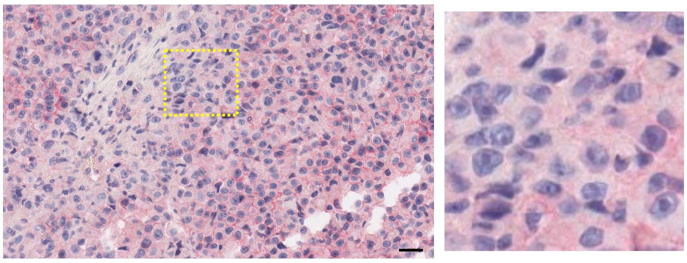

HUMSM #H17-11620

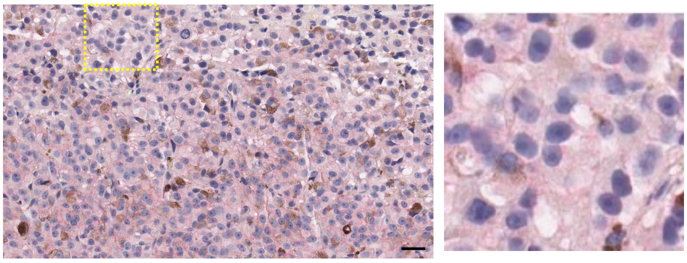

Heterogeneous expression of LKB1 in human metastatic uveal melanoma. Immunohistochemical stainings for LKB1 performed on skin metastasis of human uveal melanomas. Bar= 30 μm

Proteau_ Appendix Figure S2

A

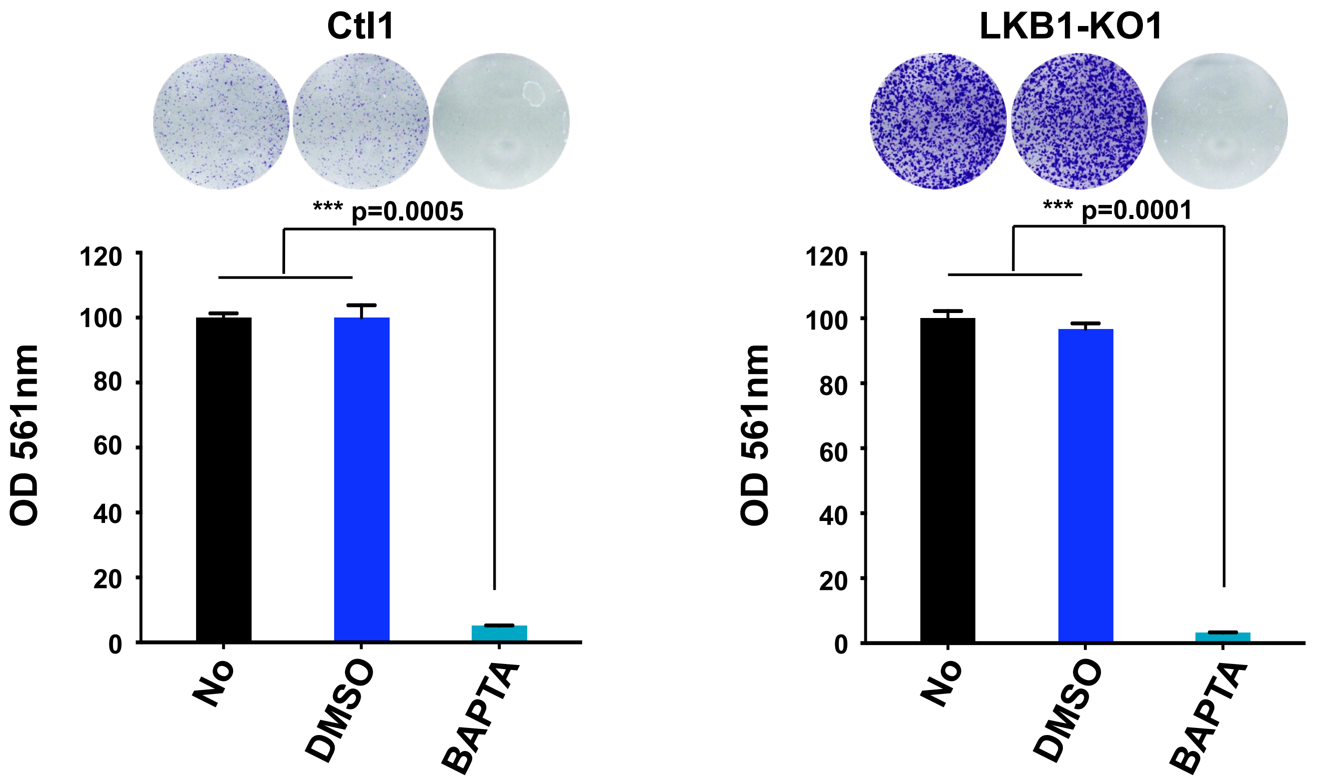

B

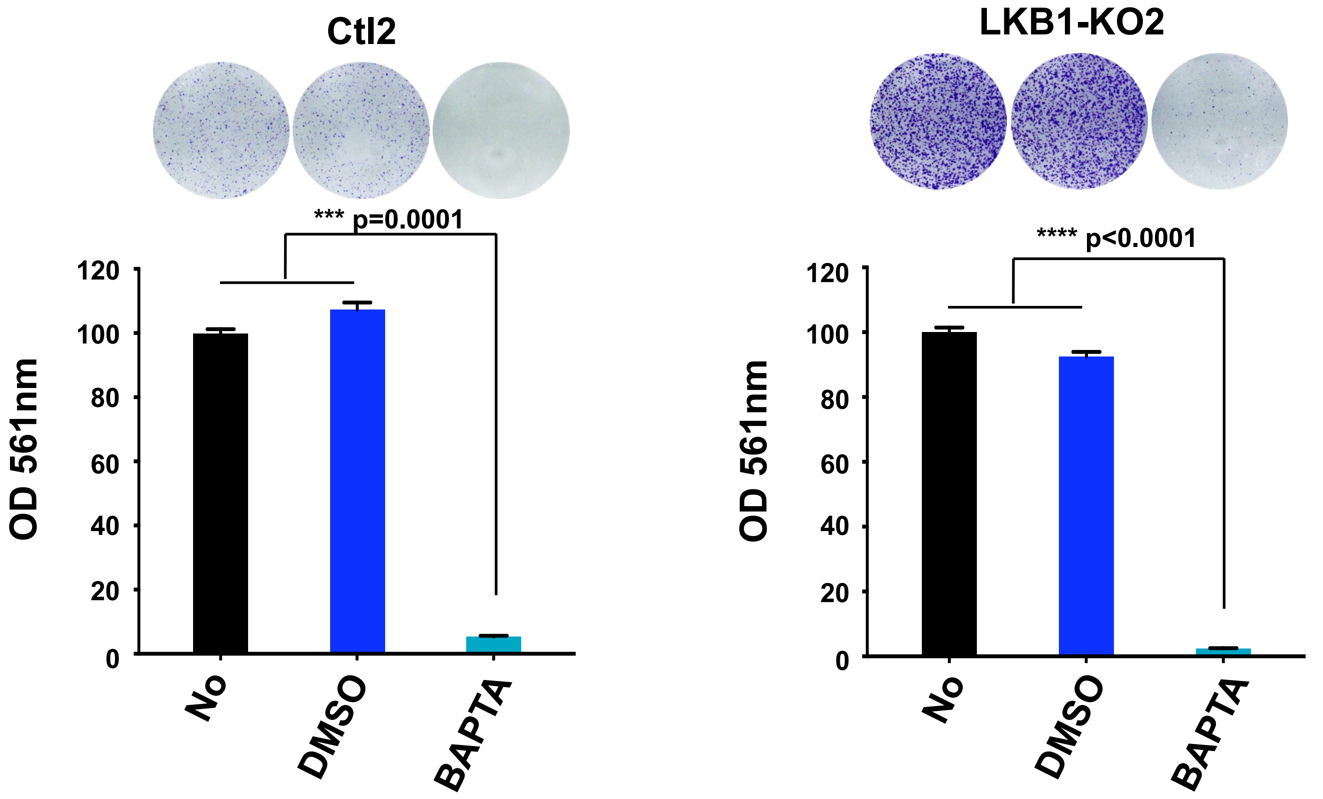

Role of calcium in uveal melanoma cell proliferation. A-B. Colony formation ability of OMM1.3 Ctl1, LKB1-KO1 (A), OMM1.3 Ctl2, LKB1-KO2 (B) cells nontreated or in presence of DMSO or BAPTA (5µM) for 7 days. Cells were seeded at low density. Representative images and crystal violet quantification at OD 561nm are shown, n=3. Data are mean ± SD.

Proteau_ Appendix Figure S3

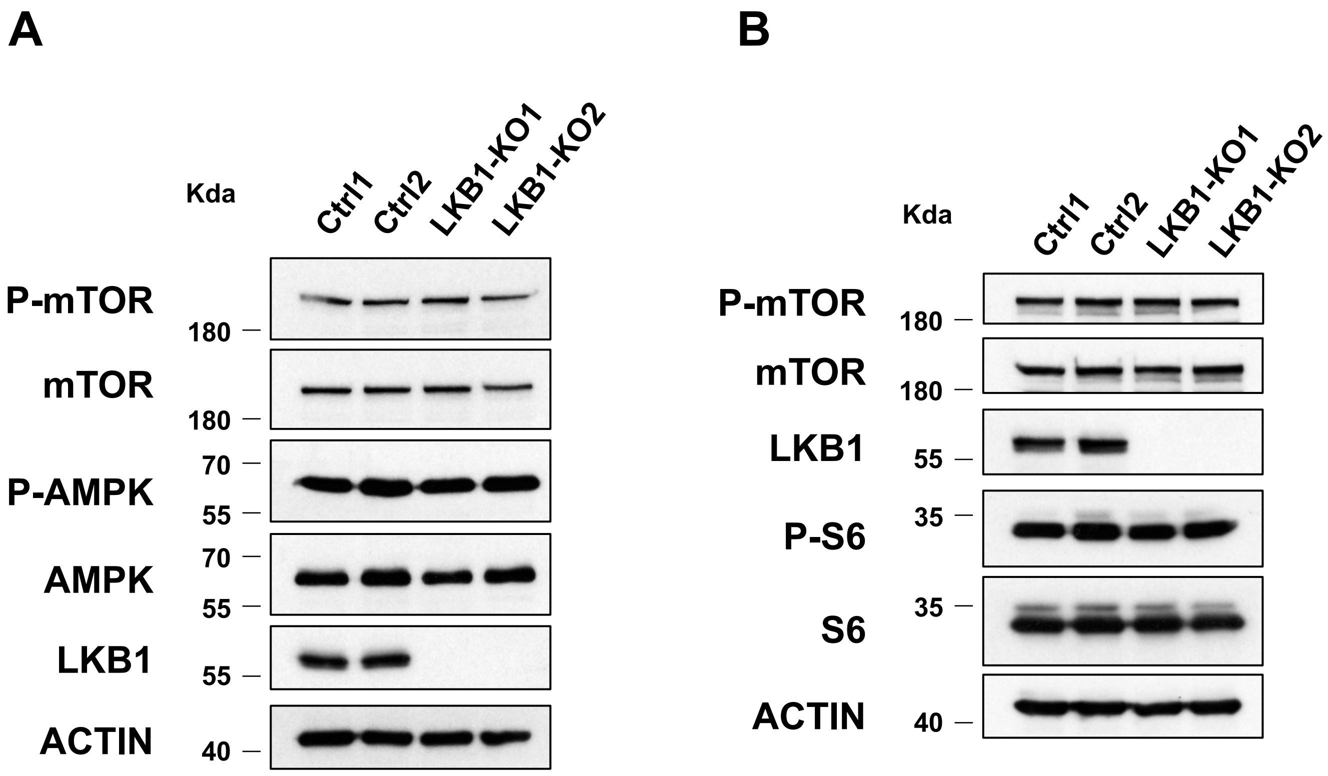

mTOR signalling in uveal melanoma. A. Immunoblot of mTOR, AMPK and their respective phosphorylation and LKB1 in OMM1.3 Ctl and LKB1-KO cells. B-Actin was used as a loading control. Representative images of three independent experiments are shown. **B.** Immunoblot of mTOR, S6 and their respective phosphorylation and LKB1 in OMM1.3 Ctl and LKB1-KO cells. B-Actin was used as a loading control. Representative images of three independent experiments are shown.

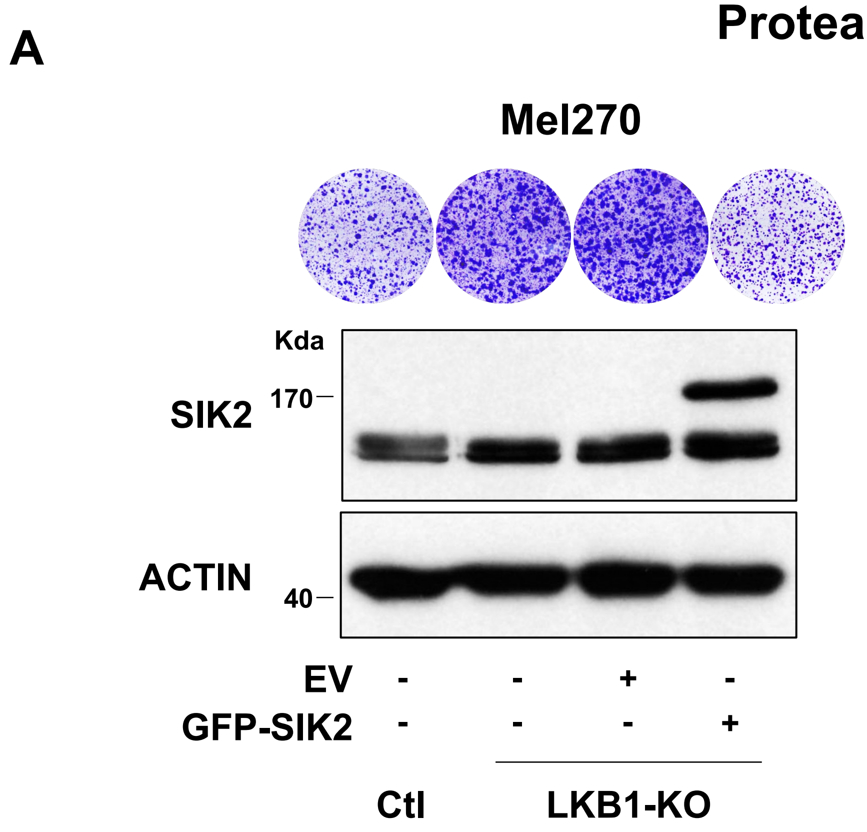

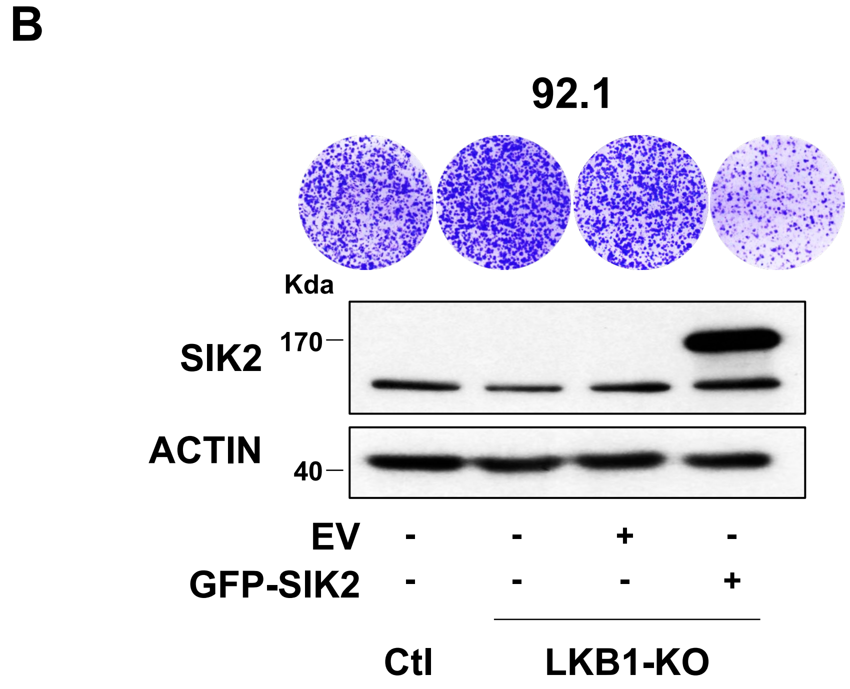

SIK2 is regulating metastatic uveal melanoma cell proliferation downstream LKB1. Human Mel270 and 92.1 primary uveal melanoma cells Ctl or LKB1-KO cells were noninfected (left) or LKB1-KO cells were infected with an empty vector (EV) or a vector encoding SIK2-WT. Colony formation assay and immunoblot of SIK2 are shown. B-Actin was used as a loading control. Representative images of three independent experiments are shown.

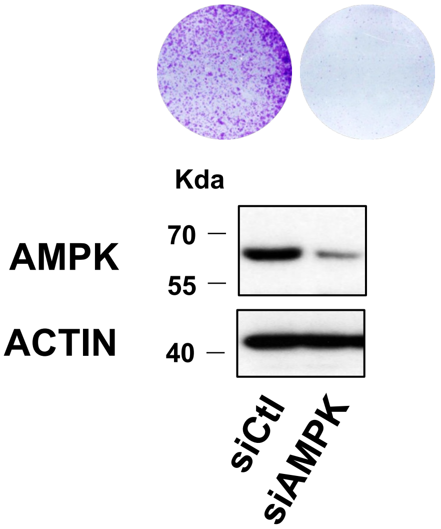

AMPK knockdown in uveal melanoma. AMPK protein level in OMM1.3 cells treated with control siRNA (siCtl) or an AMPK siRNA (siAMPK). B-Actin was used as a loading control. Representative images of three independent experiments are shown. Colony formation assay of OMM1.3 cells grown for 10 days in the same conditions. 75 000 cells were seeded. Representative images of three independent experiments
