# Supplementary material for: LKB1‐SIK2 loss drives uveal melanoma proliferation and hypersensitivity to SLC8A1 and ROS inhibition
Source: EMBO Mol Med. 2023 Nov 15;15(12):e17719. doi: 10.15252/emmm.202317719 (PMC10701601; doi:10.15252/emmm.202317719)
Supplement: Supplementary file 2 — Expanded View Figures PDF [file EMMM-15-e17719-s004.pdf]

Expanded View Figures

Figure EV1. LKB1 deletion enhances metastatic uveal melanoma proliferation.

A Schematic of the CRISPR-Cas9 kinome screen with Log_{10}-transformed MAGeCK robust ranking aggregation (RRA) scores for either depletion (left) or enrichment (right) of sgRNAs in OMM1.3 cells at D35 compared to D0.

B (Bottom) Immunoblot of LKB1 in the indicated whole-cell lysates of OMM1.3 pooled LKB1-CRISPR cell lines. HSP90 was used as a loading control. (Top) Colony formation assay of OMM1.3 Ctl1 and pooled LKB1-KD or KO cells grown for 10 days. Representative images of three independent experiments are shown.

C–F (Bottom) Immunoblot of LKB1 in the indicated whole-cell lysates of pooled LKB1-CRISPR generated in human OMM2.5 and OMM1 metastatic uveal melanoma cells and human 92.1 and Mel270 primary uveal melanoma cells. B-actin was used as a loading control. (Top) Colony formation assay of Ctl and pooled LKB1-KO cells grown for 10 days. Representative images of three independent experiments are shown.

G OMM1.3 Ctl2 and LKB1-KO2 cells were noninfected (left) or infected with a vector-encoding FLAG-tagged kinase-dead (FLAG-LKB1-KD) or wild-type (FLAG-LKB1-WT) LKB1. Colony formation assay (top) and immunoblot (bottom) of LKB1 for the indicated cell lines are shown. B-actin was used as a loading control. Representative images of three independent experiments are shown.

© 2023 The Authors

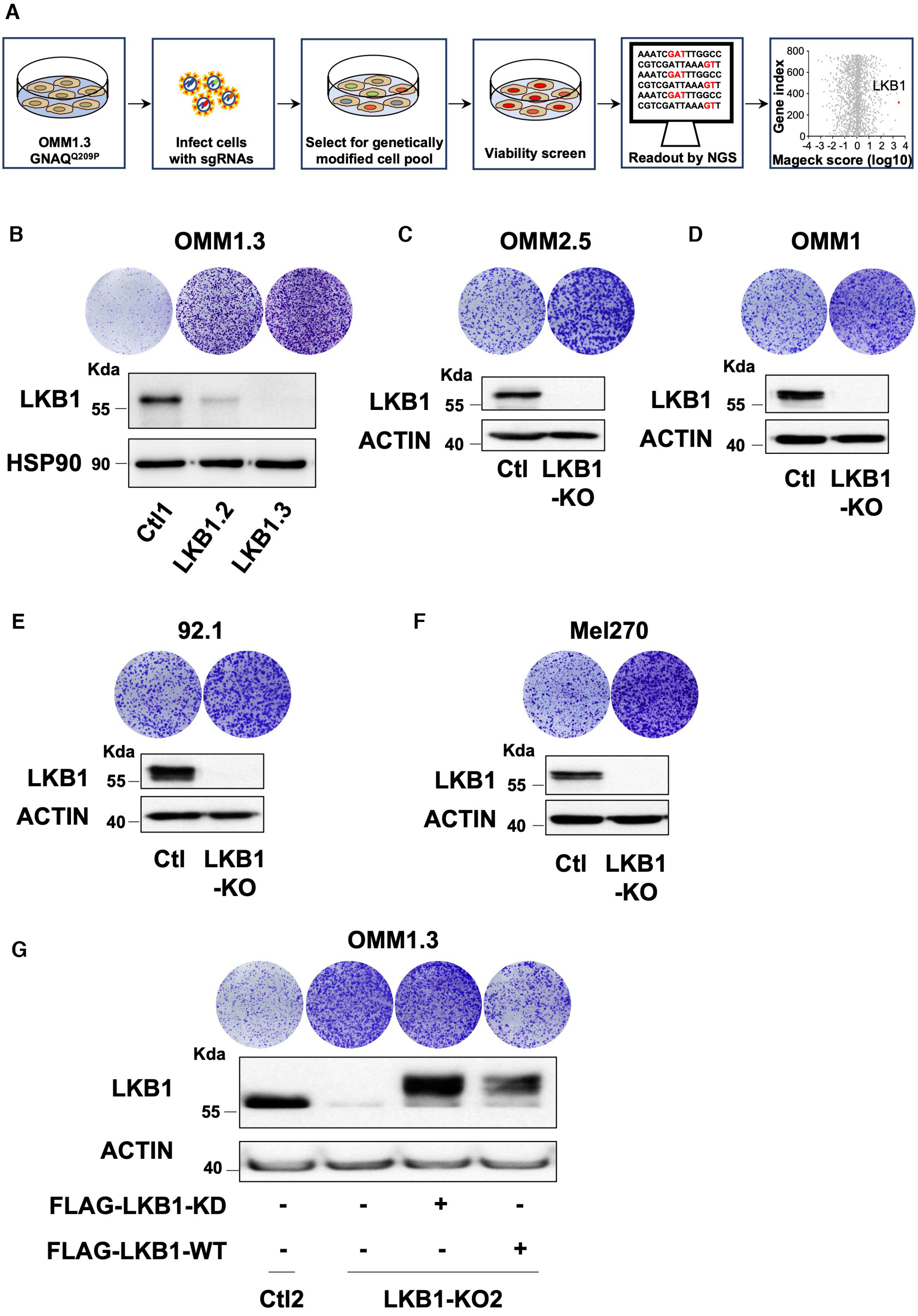

Figure EV1.

 © 2023 The Authors

Figure EV2. SLC8A1 is required for metastatic uveal melanoma cell proliferation.

A Overall survival stratified by SLC8A2 mRNA expression (median) from UM-TCGA dataset (tumors $n = 80$); P-value, log-rank test.

B, C Staining of skin metastasis of human uveal melanomas with human-positive control probe (PPIB) or with negative control probe (DapB). Scale bars represent 20 μm.

D RT–qPCR analysis of SLC8A1 mRNA level in LKB1-KO2 OMM1.3 cells treated with control siRNA (siCtl) or two different SLC8A1 siRNA (siSLC8A1). Data represent mean ± SD of three biological replicates (unpaired t-test with Welsch's correction); ***$P = 0.0005$/**$P = 0.0023$.

E Colony formation assay of LKB1-KO2 OMM1.3 cells treated as in (D) grown for 10 days. A total of 75,000 cells were seeded. Representative images and crystal violet quantification at OD 561 nm are shown. Data represent mean ± SD of three biological replicates (unpaired t-test with Welsch's correction); ****$P = 0.00001$/***$P = 0.0002$.

© 2023 The Authors

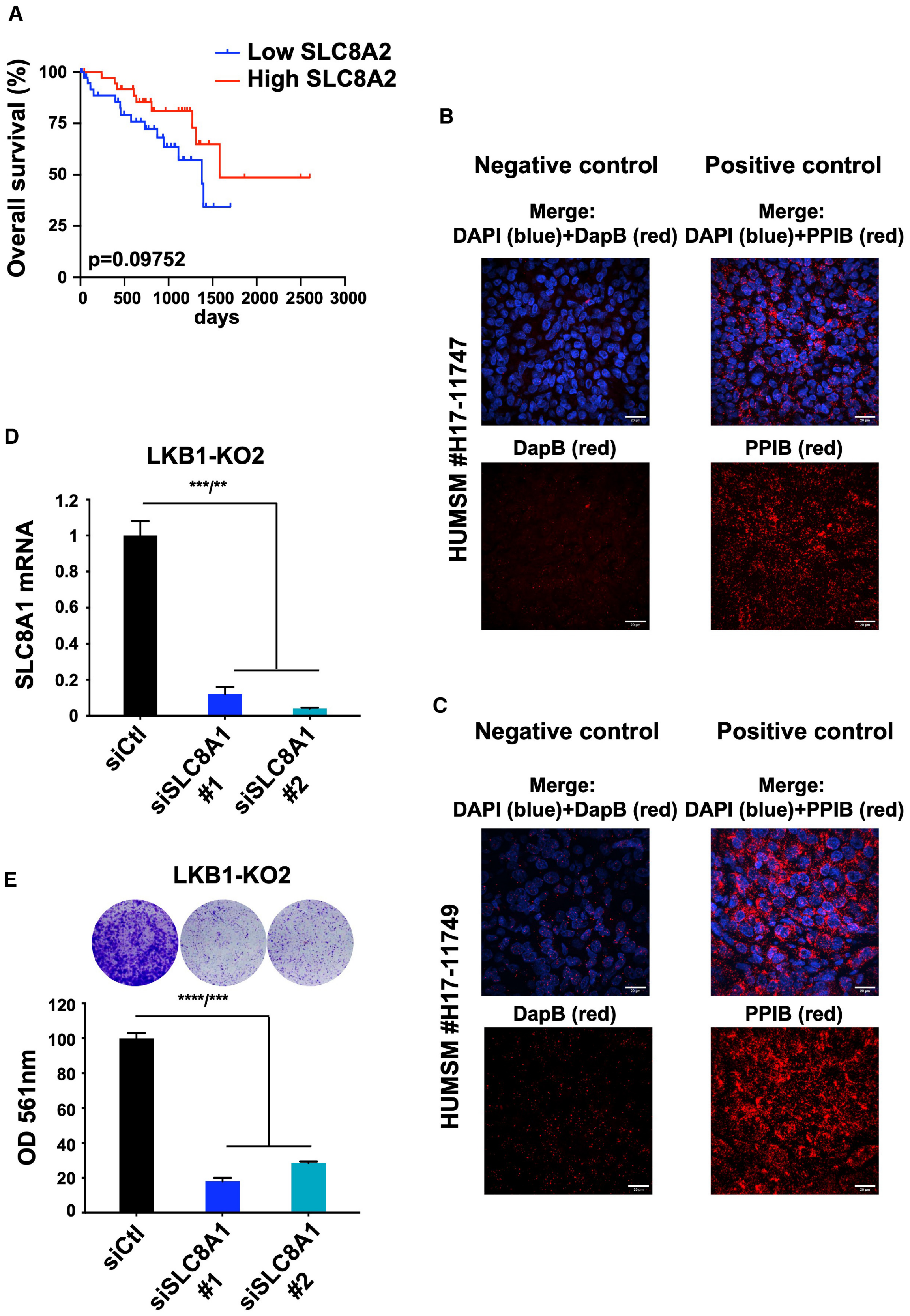

Figure EV2.

 © 2023 The Authors

Figure EV3. SIK2 deletion causes metastatic uveal melanoma cell proliferation.

A (Bottom) Immunoblot of SIK2 in the indicated whole-cell lysates of OMM1.3-pooled SIK2-CRISPR cell lines. HSP90 was used as a loading control. (Top) Colony formation assay of OMM1.3 Ctl1 and pooled SIK2-KD or KO cells grown for 10 days. Cells were seeded at low density. Representative images of three independent experiments are shown.

B Proliferation curves of OMM1.3 Ctl and SIK2-KO cells. Data represent mean ± SD of three biological replicates (unpaired *t*-test with Welsch's correction); ****P = 0.000041.

C, D (Bottom) SIK2-CRISPR-KO was generated in human OMM2.5 metastatic and human 92.1 primary uveal melanoma cells. Representative western blot assay of SIK2 is shown. B-actin was used as a loading control. (Top) Colony formation assay of control cells (Ctl) and pooled SIK2-KO cells grown for 10 days. Cells were seeded at low density. Representative images of three independent experiments are shown. Ctl cells from Fig EV1C and E have been used.

E, F OMM1.3 Ctl1, SIK2-KO1 cells and OMM1.3 Ctl2, SIK2-KO2 cells were noninfected (left) or infected with an empty vector (EV) or a vector-encoding SIK2-WT. Colony formation assay and immunoblot of SIK2 are shown. B-actin was used as a loading control. Representative images of three independent experiments are shown.

© 2023 The Authors

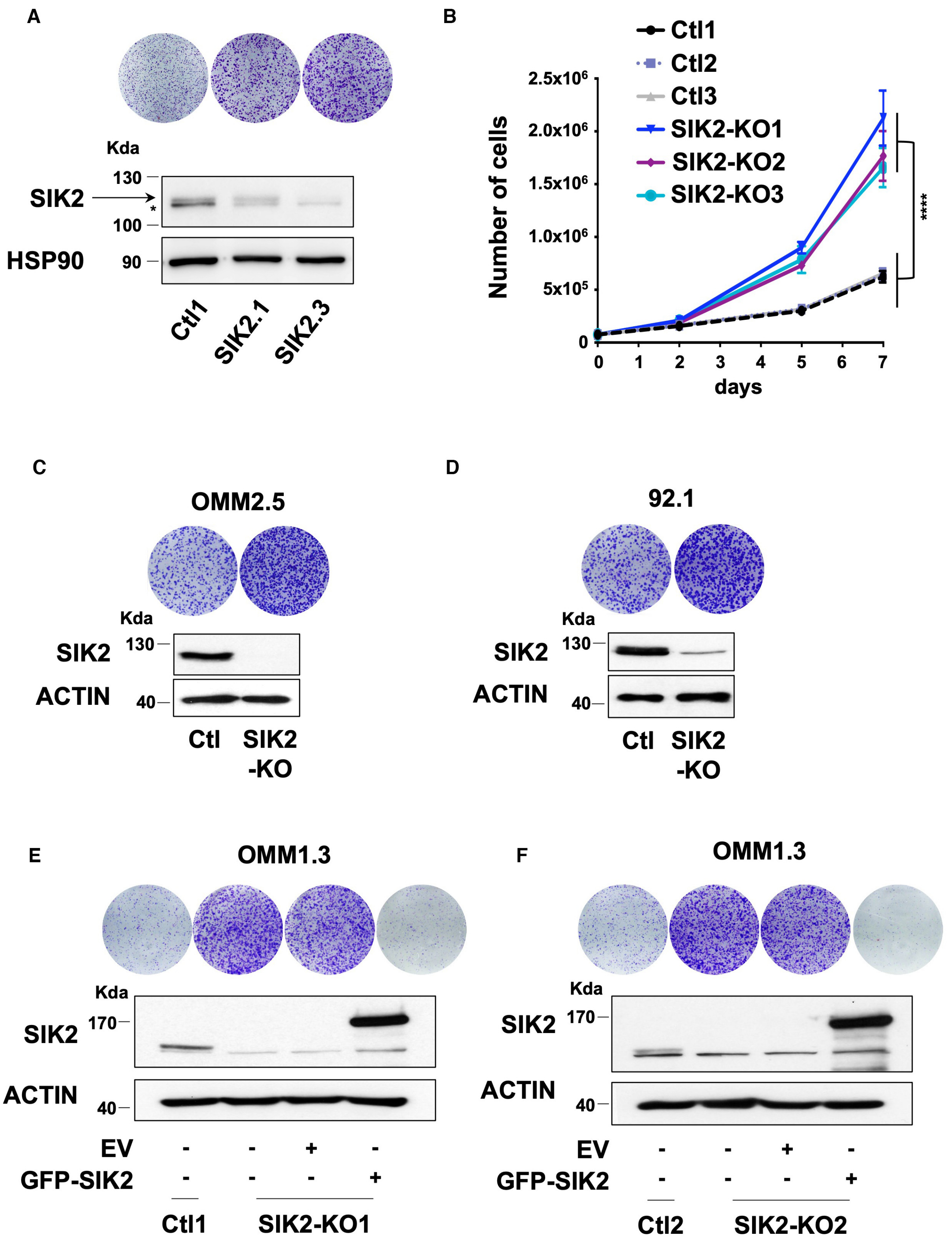

Figure EV3.

© 2023 The Authors

Figure EV4. SIK2 is regulating metastatic uveal melanoma cell proliferation downstream LKB1.

A Immunoblot of SIK2, phospho-SIK2, and LKB1 in the indicated whole-cell lysates from control, LKB1-KO, and SIK2-KO OMM1.3 cells. B-actin was used as a loading control. Representative images of three independent experiments are shown.

B LKB1-KO2 cells transfected with an empty vector (EV) or vectors encoding either a kinase-dead (SIK2 K49M) or a constitutively active (SIK2 T175D) form of SIK2. Immunoblot for SIK2 and colony formation assay are shown. B-actin was used as a loading control. Representative images of three independent experiments are shown.

C, D Human OMM2.5 and OMM1 metastatic uveal melanoma cells Ctl or LKB1-KO cells were noninfected (left) or LKB1-KO cells were infected with an empty vector (EV) or a vector-encoding SIK2-WT. Colony formation assay and immunoblot of SIK2 are shown. B-actin was used as a loading control. Representative images of three independent experiments are shown.

E RT–qPCR analysis of SLC8A1 mRNA level in OMM1.3 Ctl2 and SIK2-KO2 noninfected (left) or infected with an empty vector (EV) or a vector-encoding SIK2-WT. Data represent mean ± SD of three biological replicates (unpaired *t*-test with Welsch's correction); ***P* = 0.0068/****P* = 0.004; NS, not significant, *P* = 0.0844.

F RT–qPCR analysis of SLC8A1 mRNA level in OMM1.3 Ctl2 and LKB1-KO2 noninfected (left) or infected with an empty vector (EV) or a vector-encoding SIK2-WT. Data represent mean ± SD of three biological replicates (unpaired *t*-test with Welsch's correction) ***P* = 0.0069/***P* = 0076 and **P* = 0.0109.

© 2023 The Authors

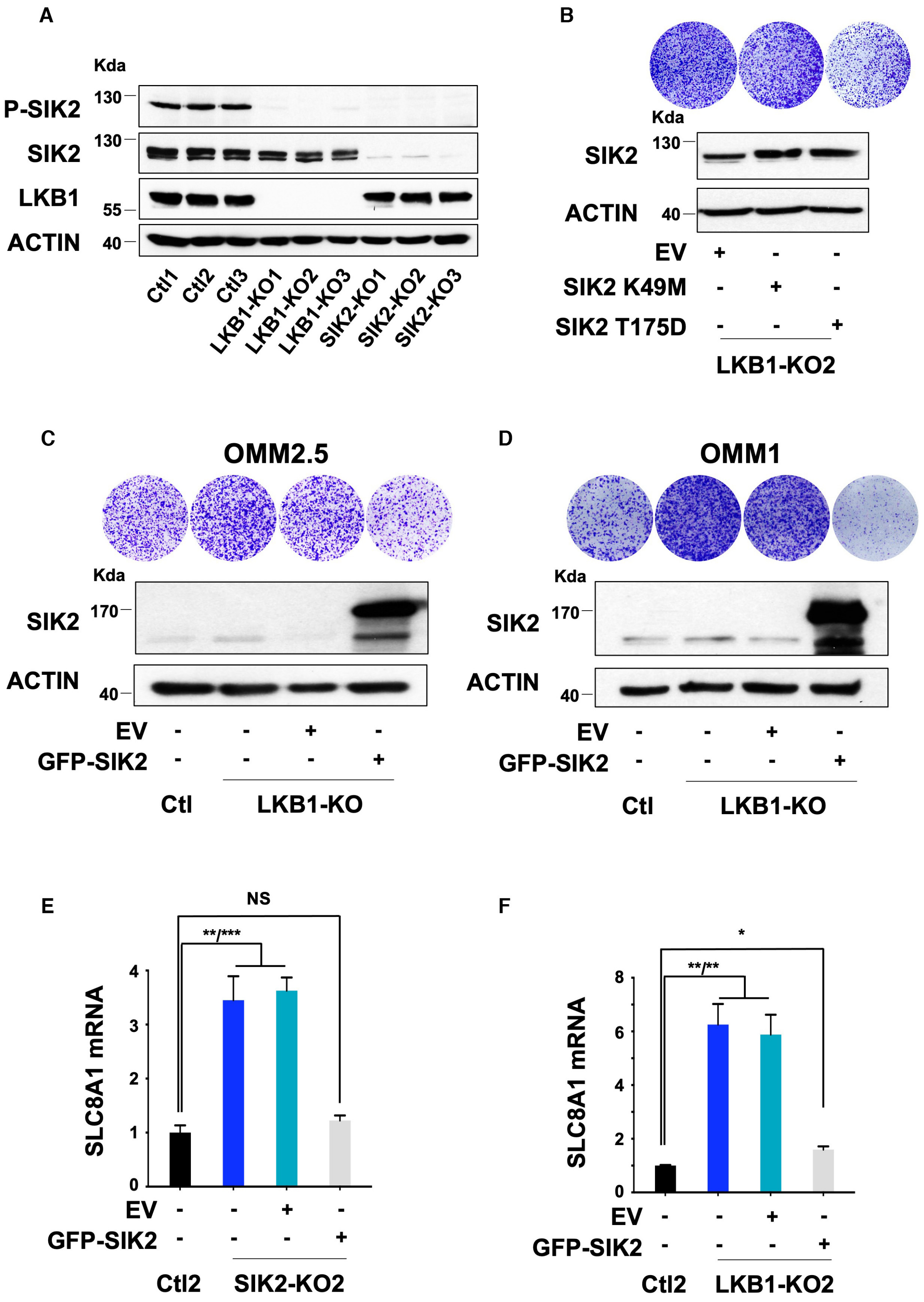

Figure EV4.

 © 2023 The Authors

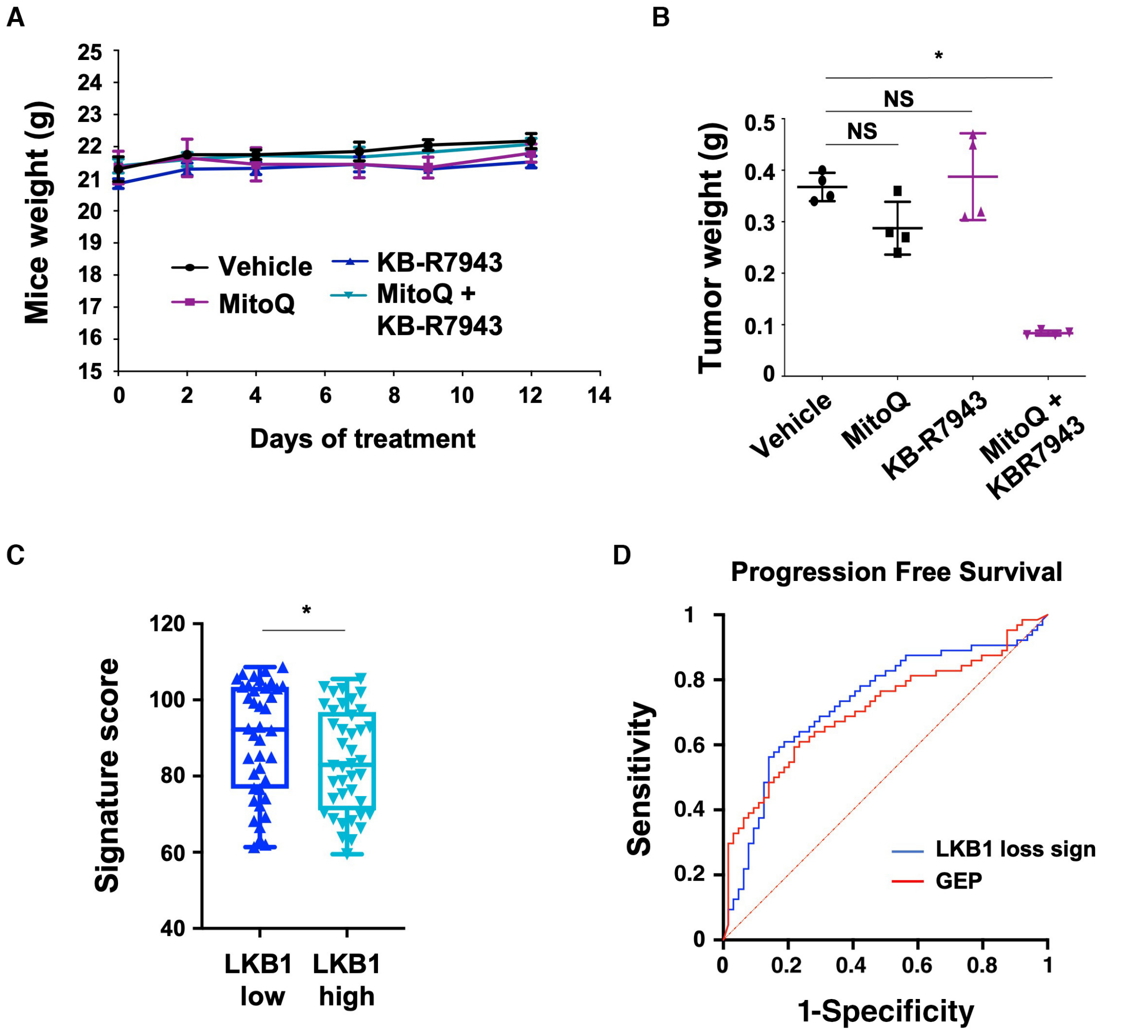

	GEP	LKB1 loss sign
**AUC**	0.75	0.78
**p-value**	4.2×10^{-5}	6.8×10^{-6}

Figure EV5. Inhibition of SLC8A1 and mitochondrial ROS trigger tumor regression.

A Body weights of mice during treatment are shown as the mean ± SEM (*n* = 4 mice, each group).

B Tumor weights of the indicated xenografts at the endpoint (12 days) are shown as the mean ± SD (*n* = 4 mice, each group). Mann–Whitney test was performed for comparison between groups. *P = 0.0286; NS, not significant, P = 0.1143.

C Representative box and whiskers plots of the LKB1 loss signature score based on LKB1 mRNA expression level (low and high) from the UM-TCGA dataset. Mann–Whitney test was performed for comparison between groups. *P = 0.0487. All points are represented.

D Time-dependent receiver operating characteristic (ROC) curves show the sensitivity and specificity of our LKB1 signature compared to the gene expression profiling signature (GEP) (Onken *et al*, 2004), for predicting the progression-free survival (Laurent *et al*, 2011a; Data ref: Laurent *et al*, 2011b).

© 2023 The Authors
