# Supplementary figures and images for: LKB1‐SIK2 loss drives uveal melanoma proliferation and hypersensitivity to SLC8A1 and ROS inhibition
Source: EMBO Mol Med. 2023 Nov 15;15(12):e17719. doi: 10.15252/emmm.202317719 (PMC10701601; doi:10.15252/emmm.202317719)

**Figure 1A**

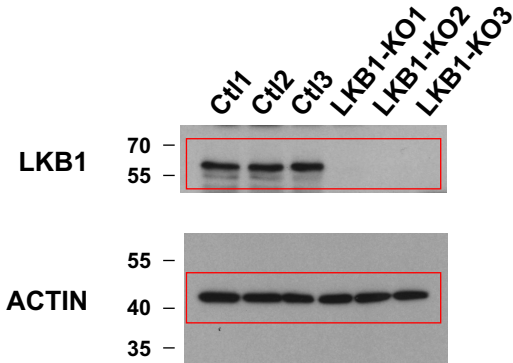

Supplement: Supplementary file 6 — Source Data for Figure 1 [file EMMM-15-e17719-s011.zip › Figure 1/1A/western LKB1_ACTIN.pdf]

**Figure 1D**

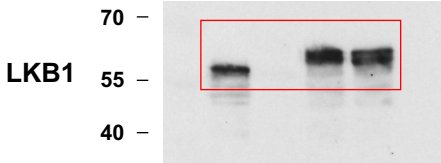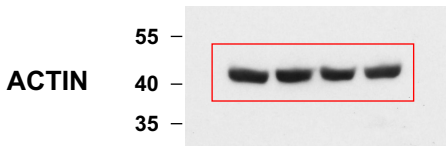

**FLAG-LKB1-KD**  
**FLAG-LKB1-WT**

|   |   |   |   |
|---|---|---|---|
| - | - | + | - |
| - | - | - | + |

---

**Ctl1**    **LKB1-KO1**

Supplement: Supplementary file 6 — Source Data for Figure 1 [file EMMM-15-e17719-s011.zip › Figure 1/1D/western LKB1_ACTIN.pdf]

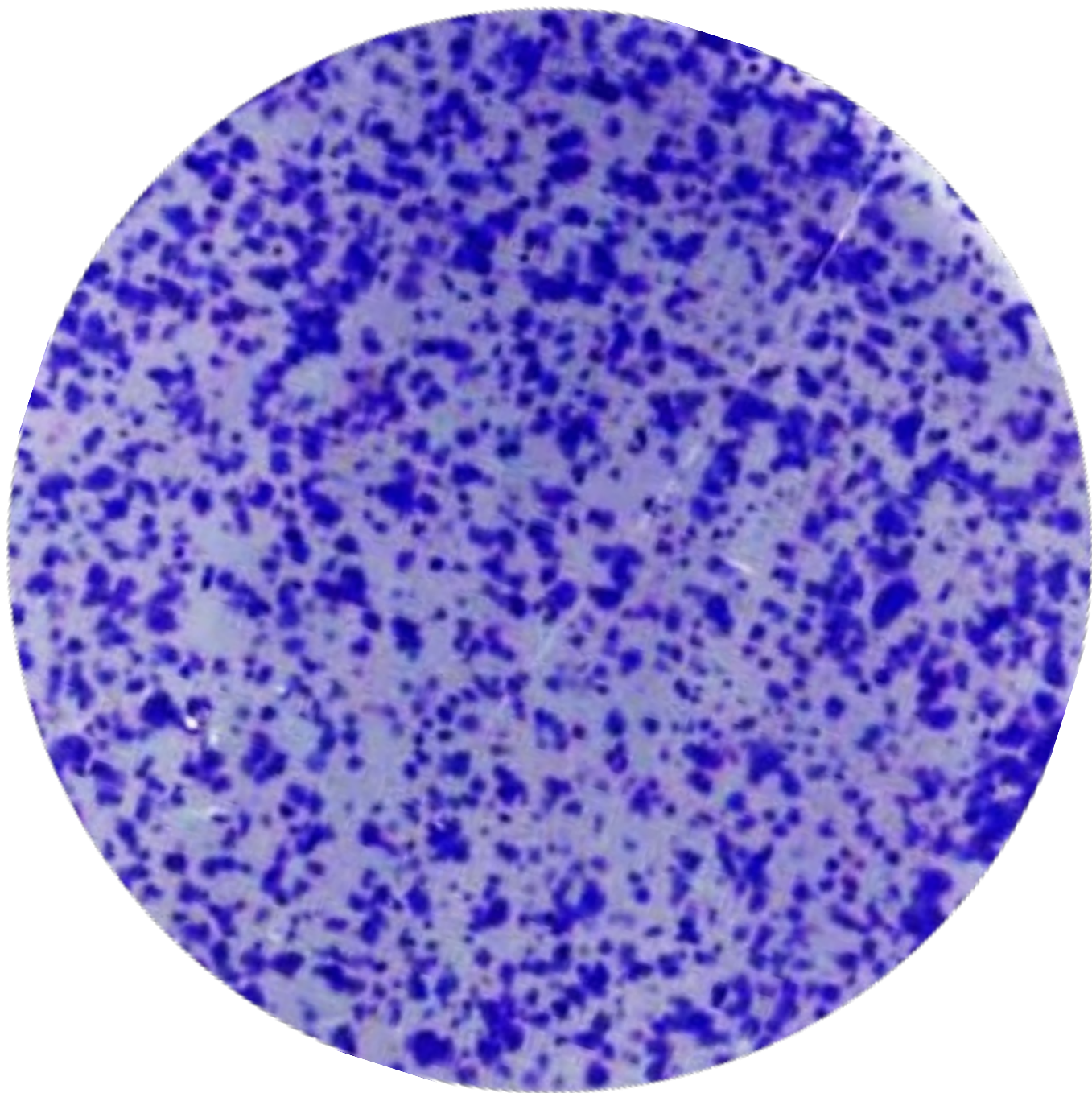

Supplement: Supplementary file 6 — Source Data for Figure 1 [file EMMM-15-e17719-s011.zip › Figure 1/1D/Flag-LKB1-KD.pdf]

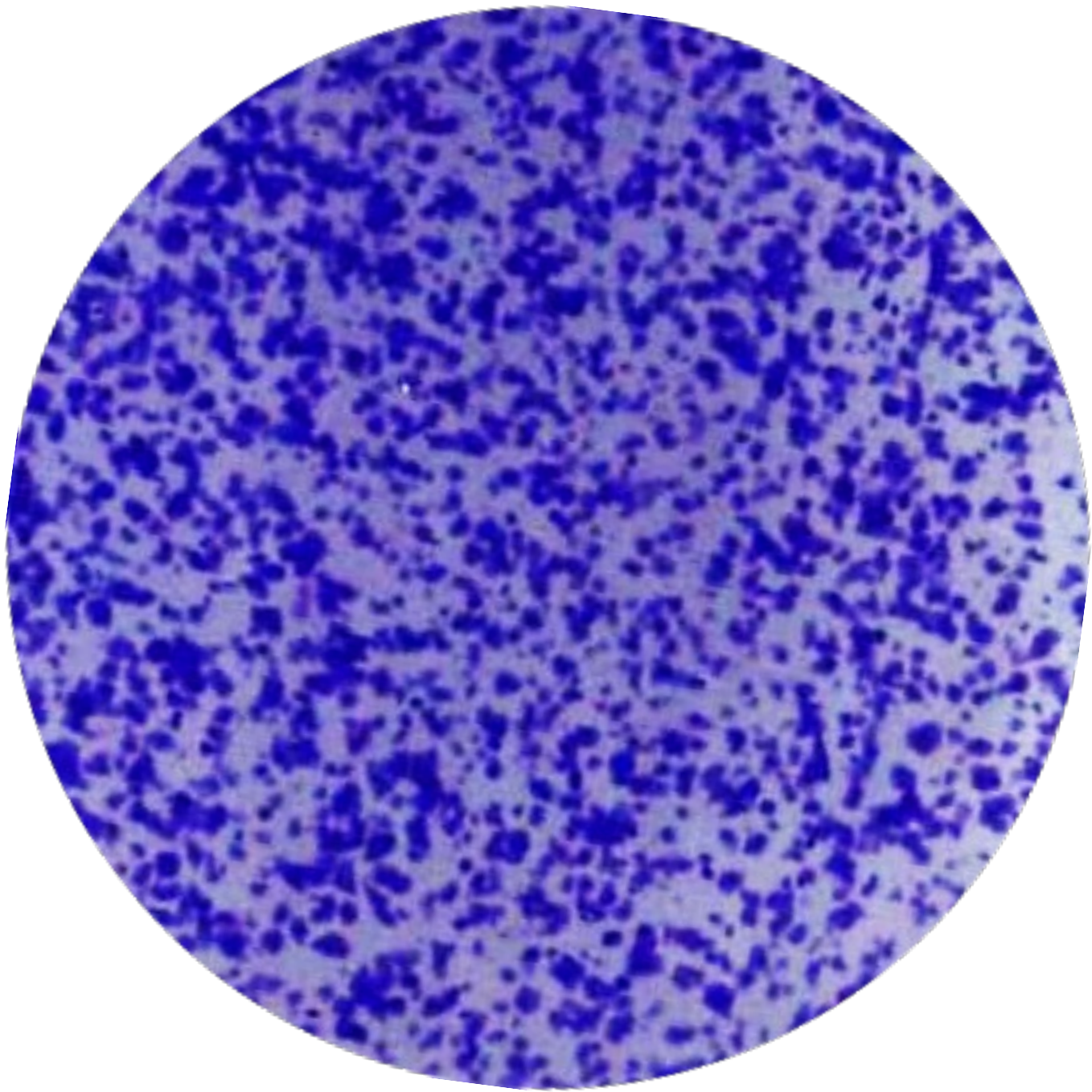

Supplement: Supplementary file 6 — Source Data for Figure 1 [file EMMM-15-e17719-s011.zip › Figure 1/1D/LKB1 KO.pdf]

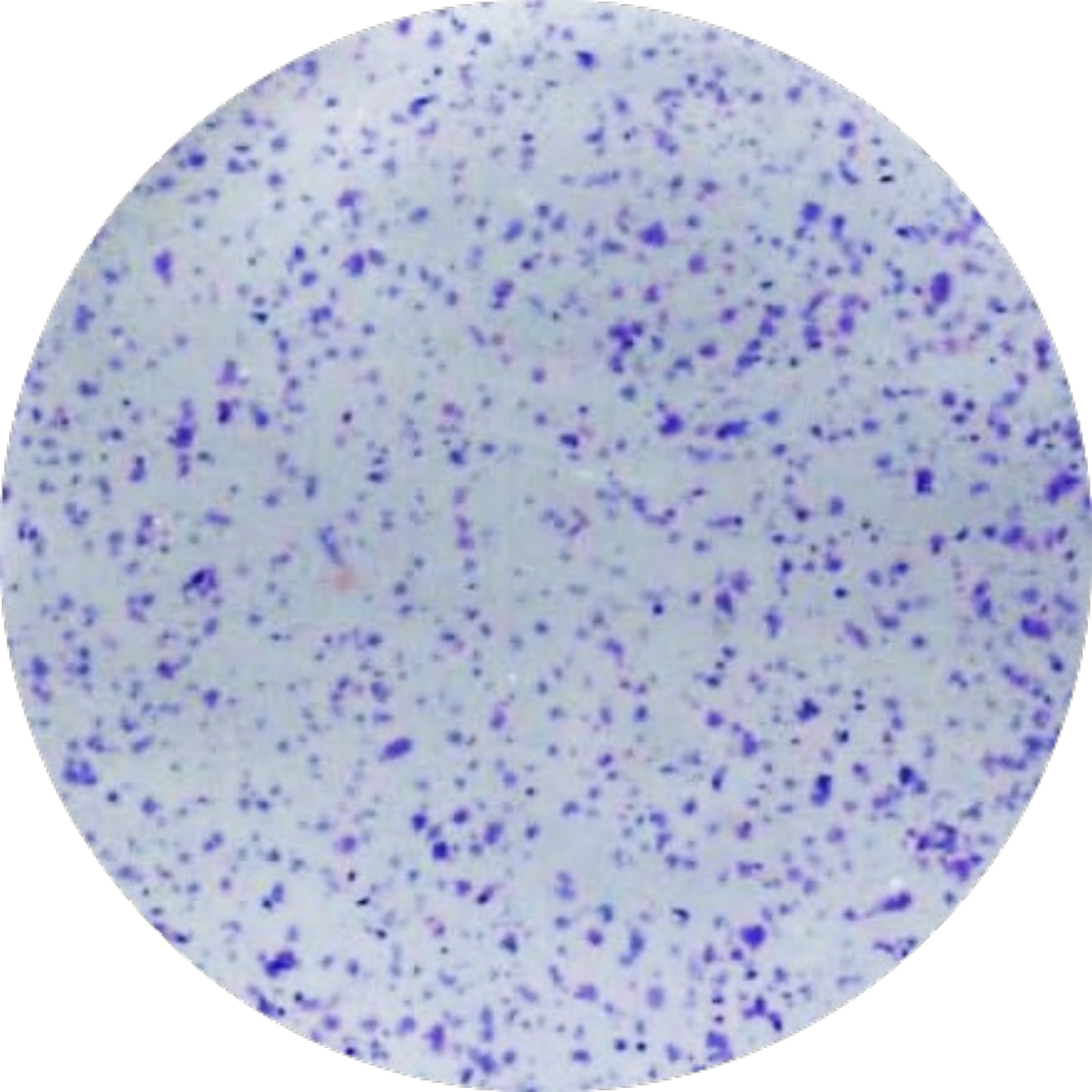

Supplement: Supplementary file 6 — Source Data for Figure 1 [file EMMM-15-e17719-s011.zip › Figure 1/1D/Ctl1.pdf]

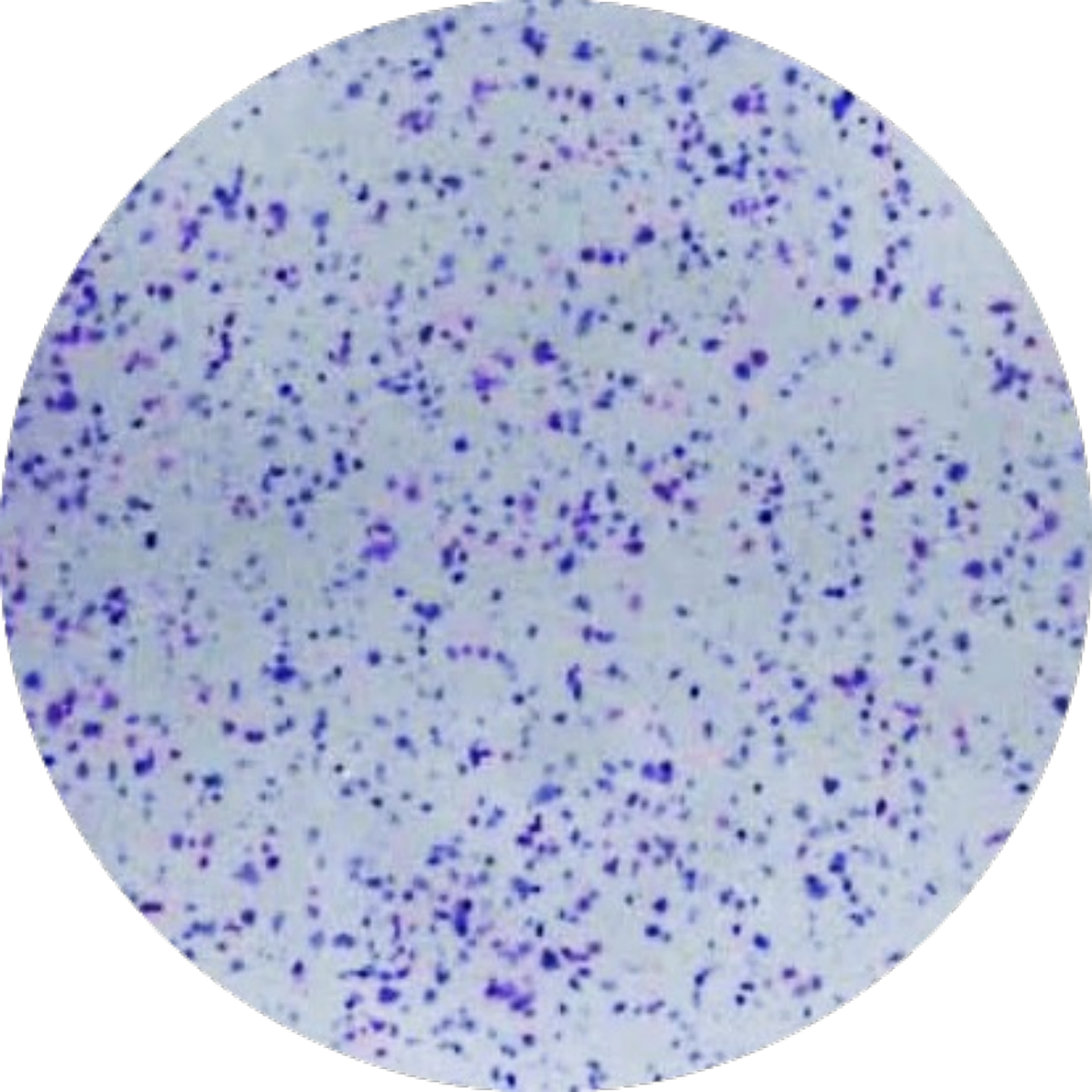

Supplement: Supplementary file 6 — Source Data for Figure 1 [file EMMM-15-e17719-s011.zip › Figure 1/1D/Flag-LKB1-WT.pdf]

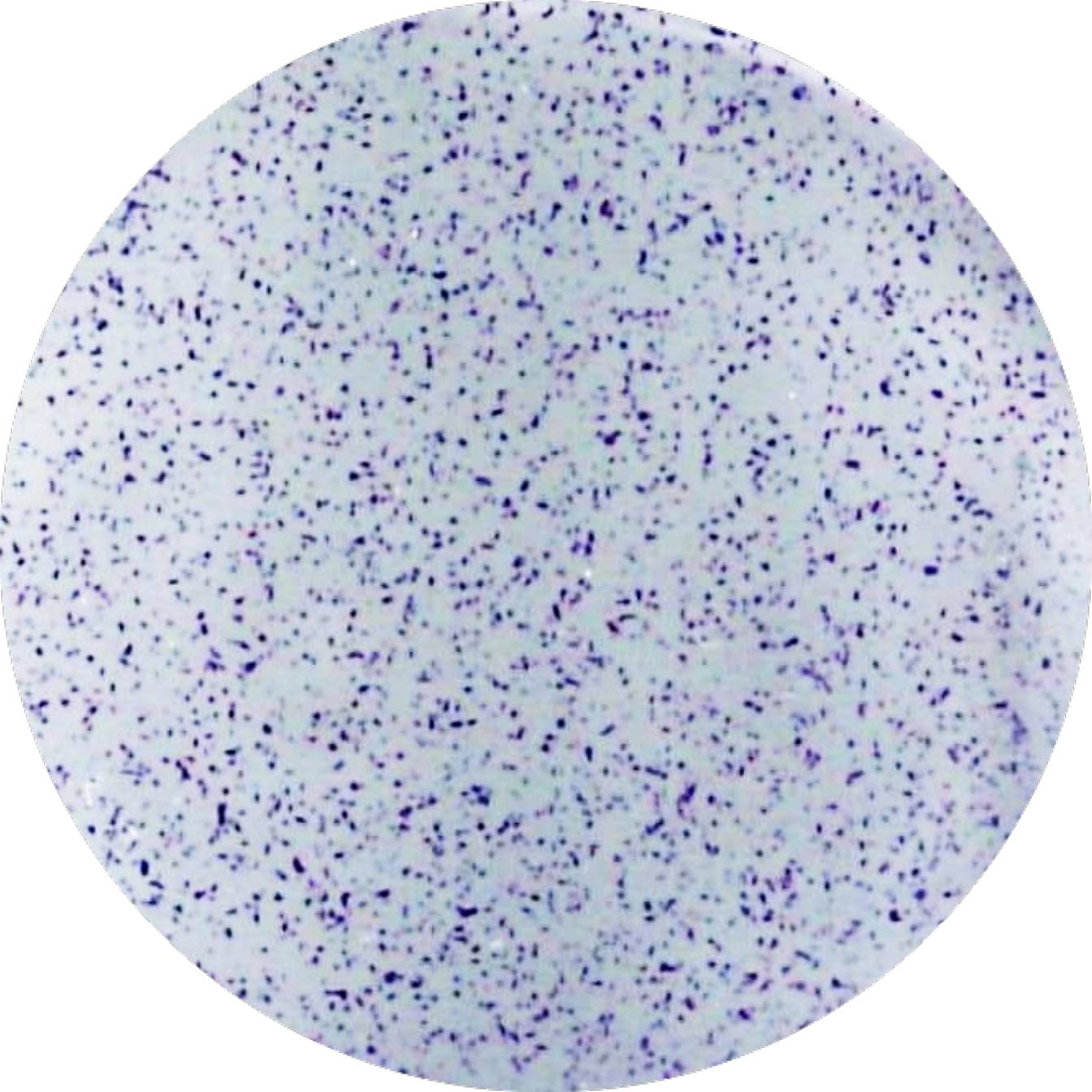

Supplement: Supplementary file 6 — Source Data for Figure 1 [file EMMM-15-e17719-s011.zip › Figure 1/1C/Ctl3.pdf]

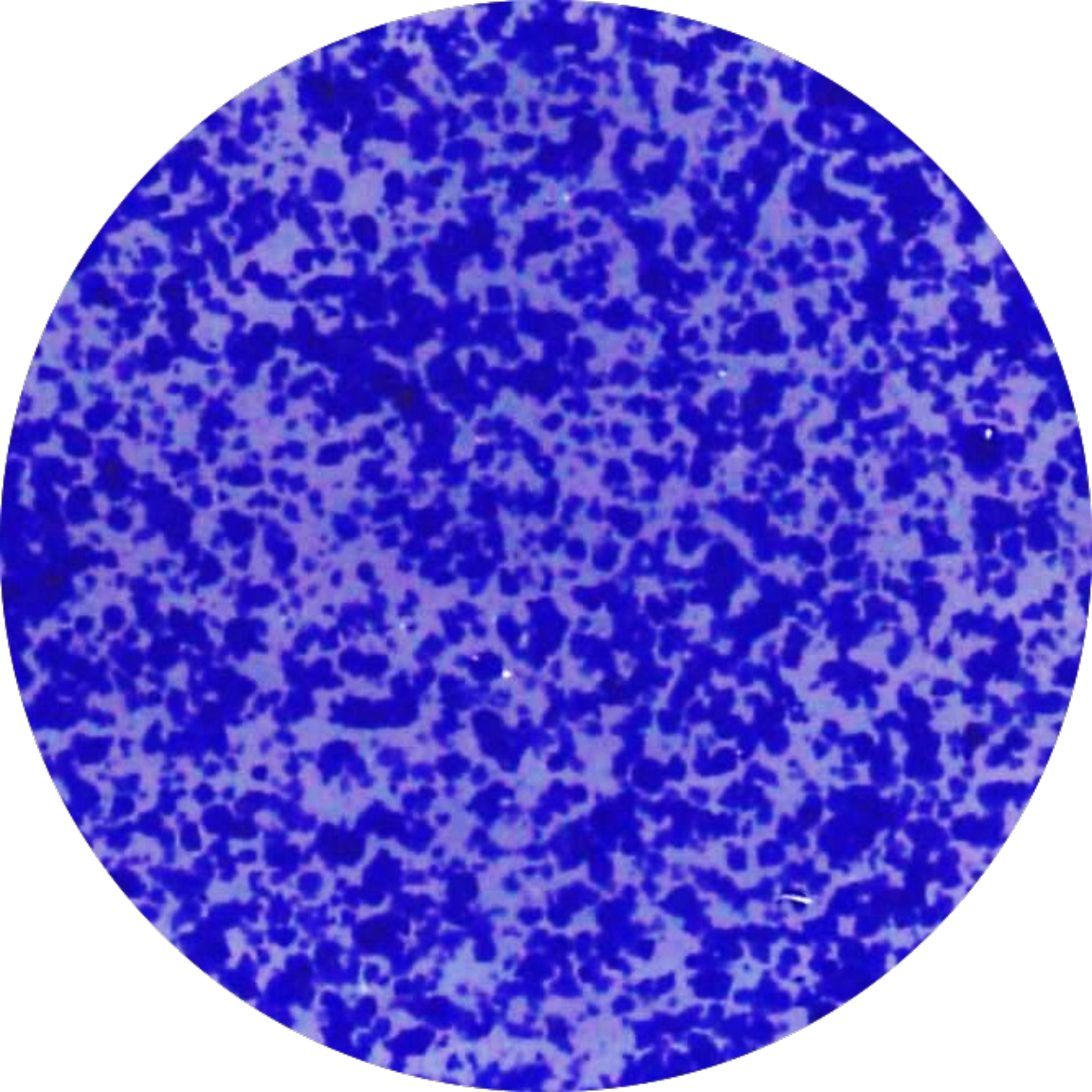

Supplement: Supplementary file 6 — Source Data for Figure 1 [file EMMM-15-e17719-s011.zip › Figure 1/1C/LKB1 KO3.pdf]

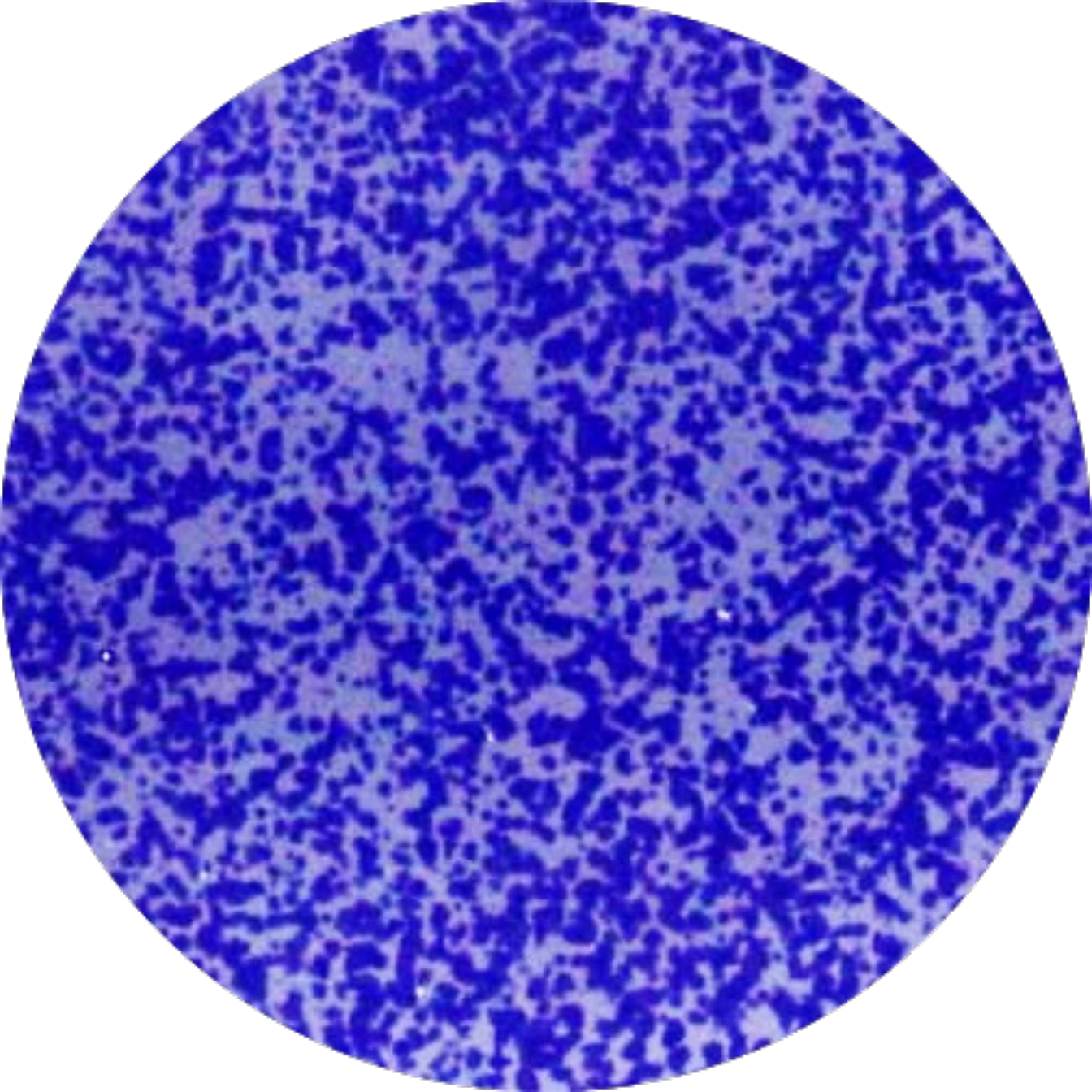

Supplement: Supplementary file 6 — Source Data for Figure 1 [file EMMM-15-e17719-s011.zip › Figure 1/1C/LKB1 KO2.pdf]

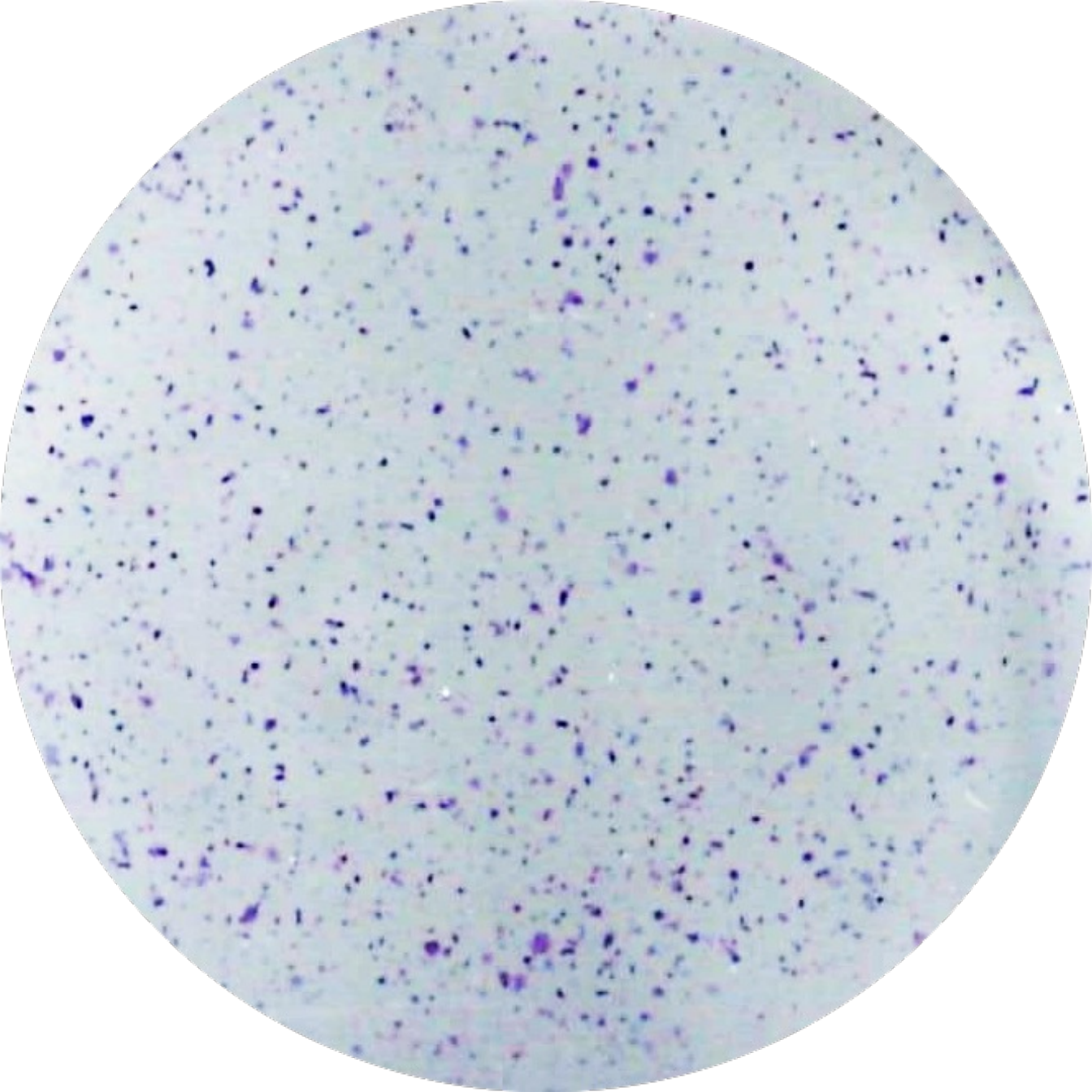

Supplement: Supplementary file 6 — Source Data for Figure 1 [file EMMM-15-e17719-s011.zip › Figure 1/1C/Ctl2.pdf]

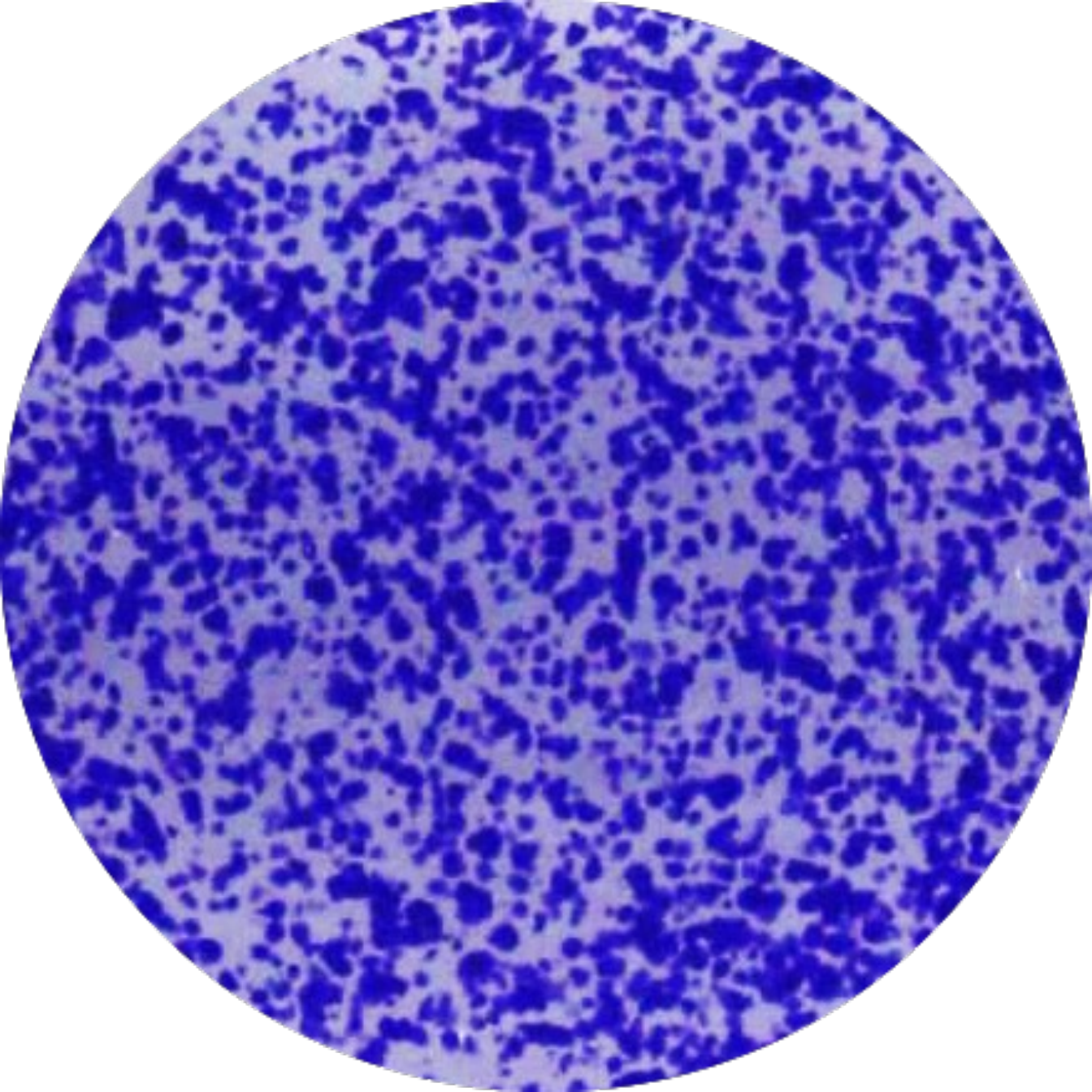

Supplement: Supplementary file 6 — Source Data for Figure 1 [file EMMM-15-e17719-s011.zip › Figure 1/1C/LKB1 KO1.pdf]

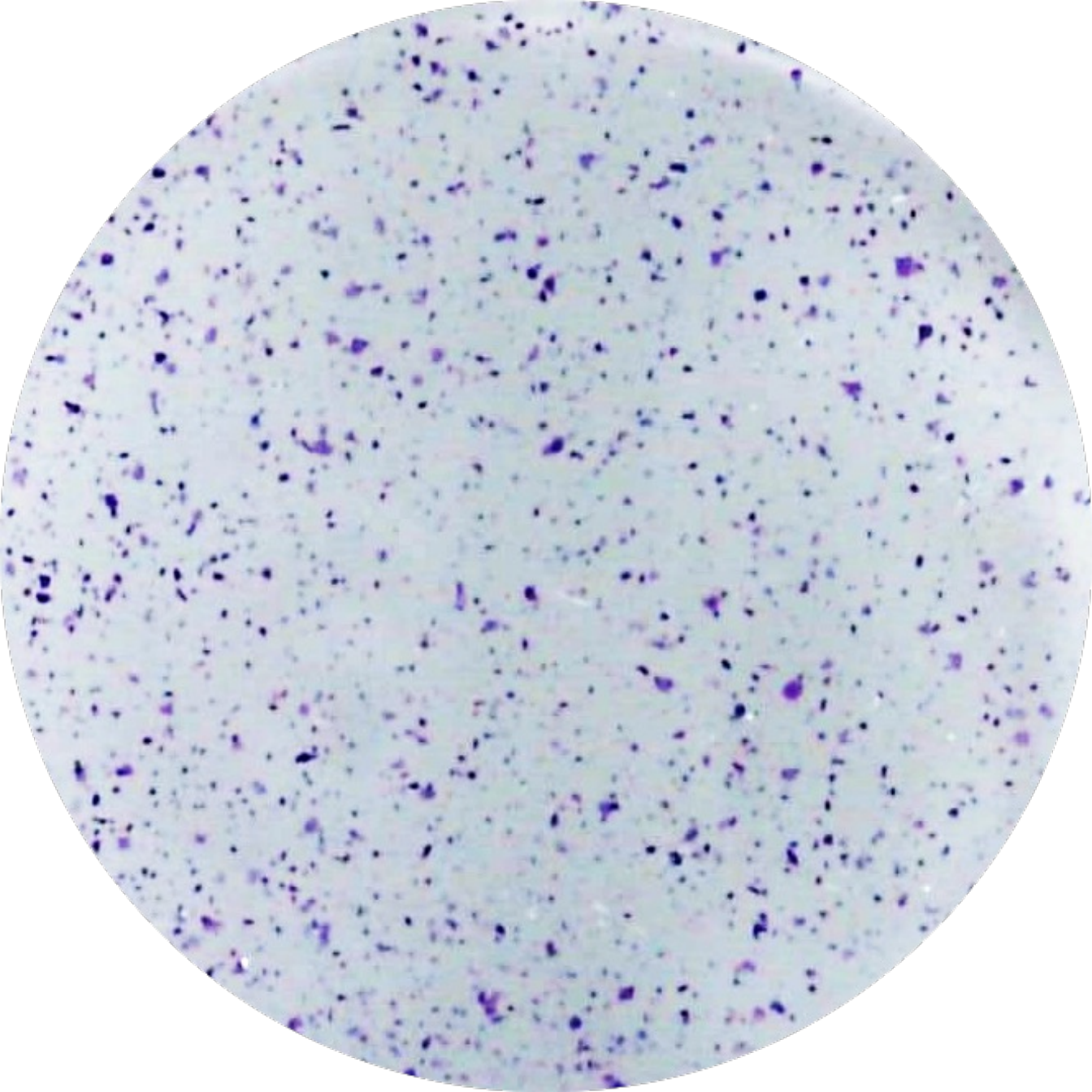

Supplement: Supplementary file 6 — Source Data for Figure 1 [file EMMM-15-e17719-s011.zip › Figure 1/1C/Ctl1.pdf]

**Figure 2C**

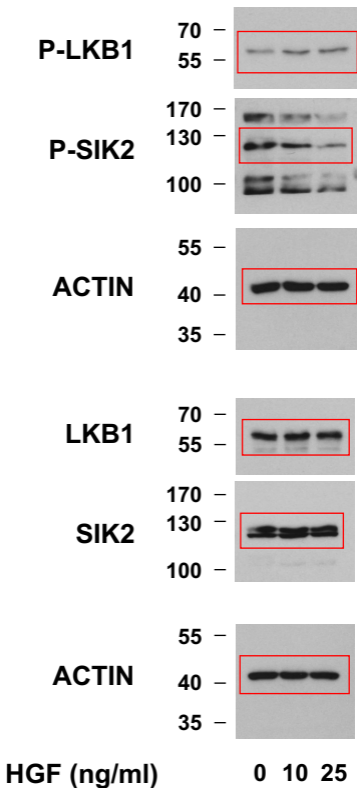

Supplement: Supplementary file 7 — Source Data for Figure 2 [file EMMM-15-e17719-s006.zip › Figure 2/2C/western LKB1_SIK2_ACTIN.pdf]

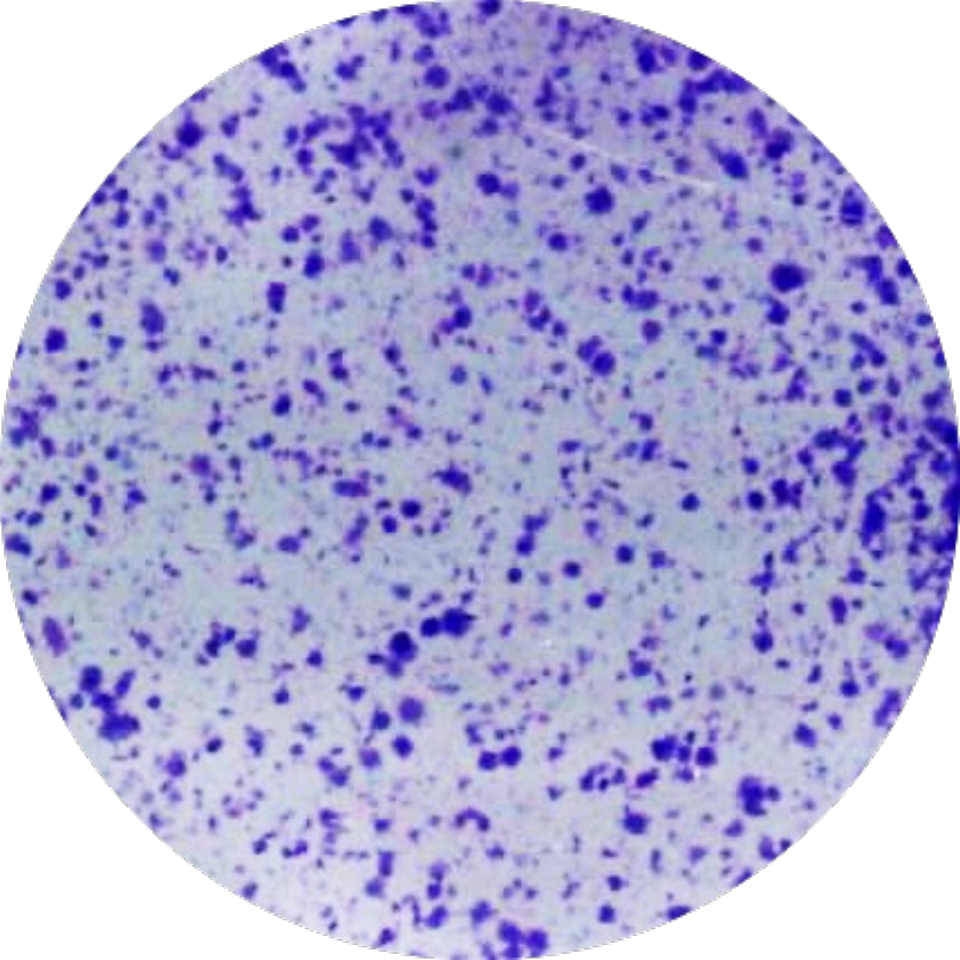

Supplement: Supplementary file 7 — Source Data for Figure 2 [file EMMM-15-e17719-s006.zip › Figure 2/2D/HGF_25.pdf]

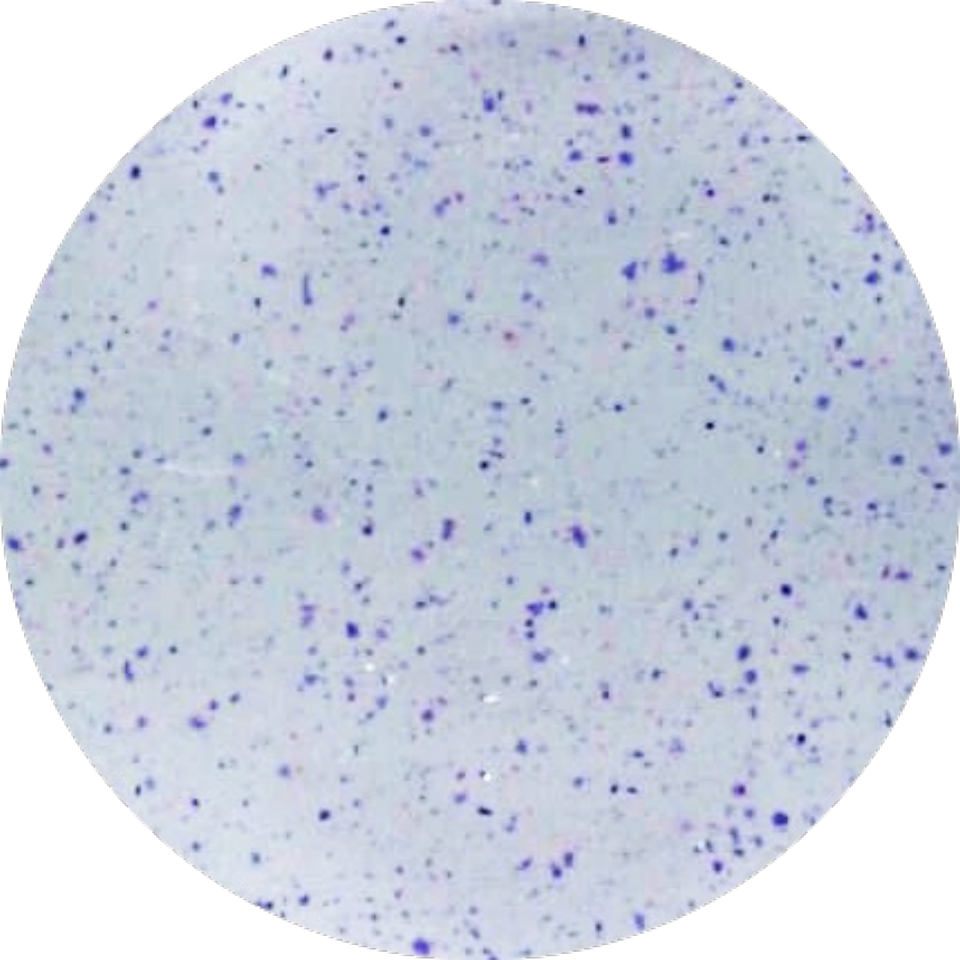

Supplement: Supplementary file 7 — Source Data for Figure 2 [file EMMM-15-e17719-s006.zip › Figure 2/2D/HGF_0.pdf]

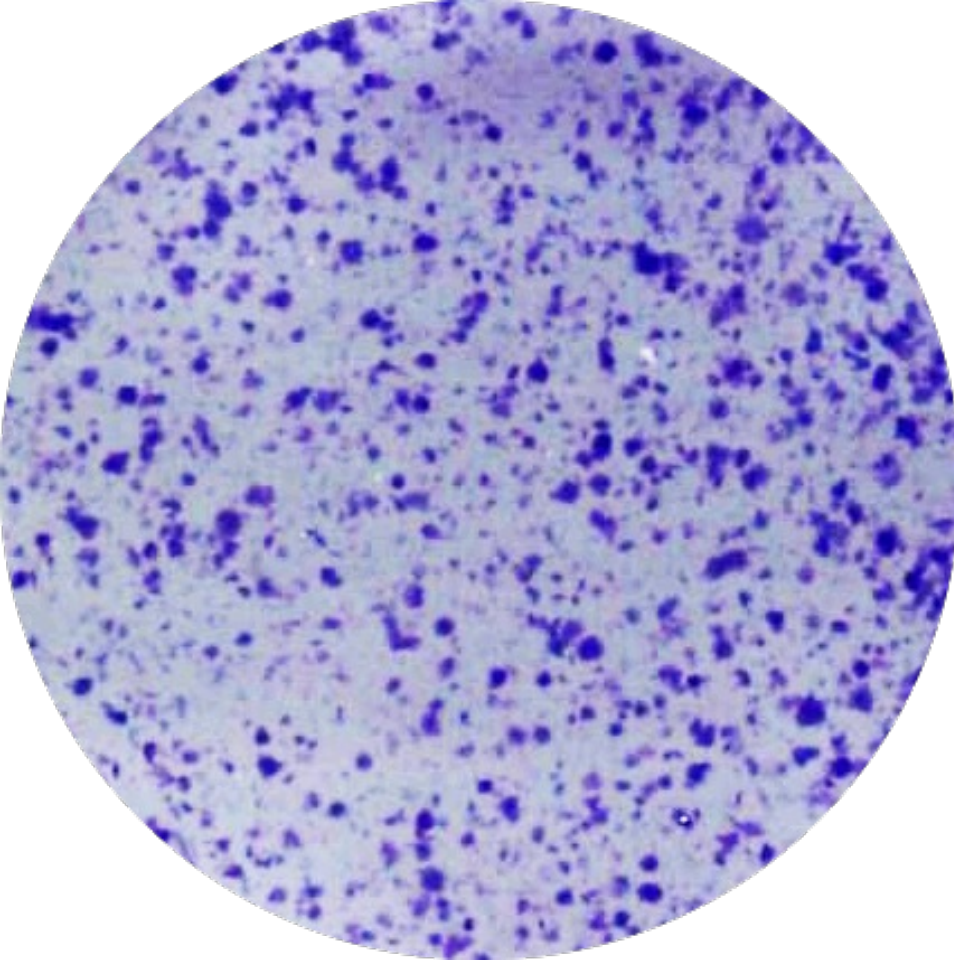

Supplement: Supplementary file 7 — Source Data for Figure 2 [file EMMM-15-e17719-s006.zip › Figure 2/2D/HGF_10.pdf]

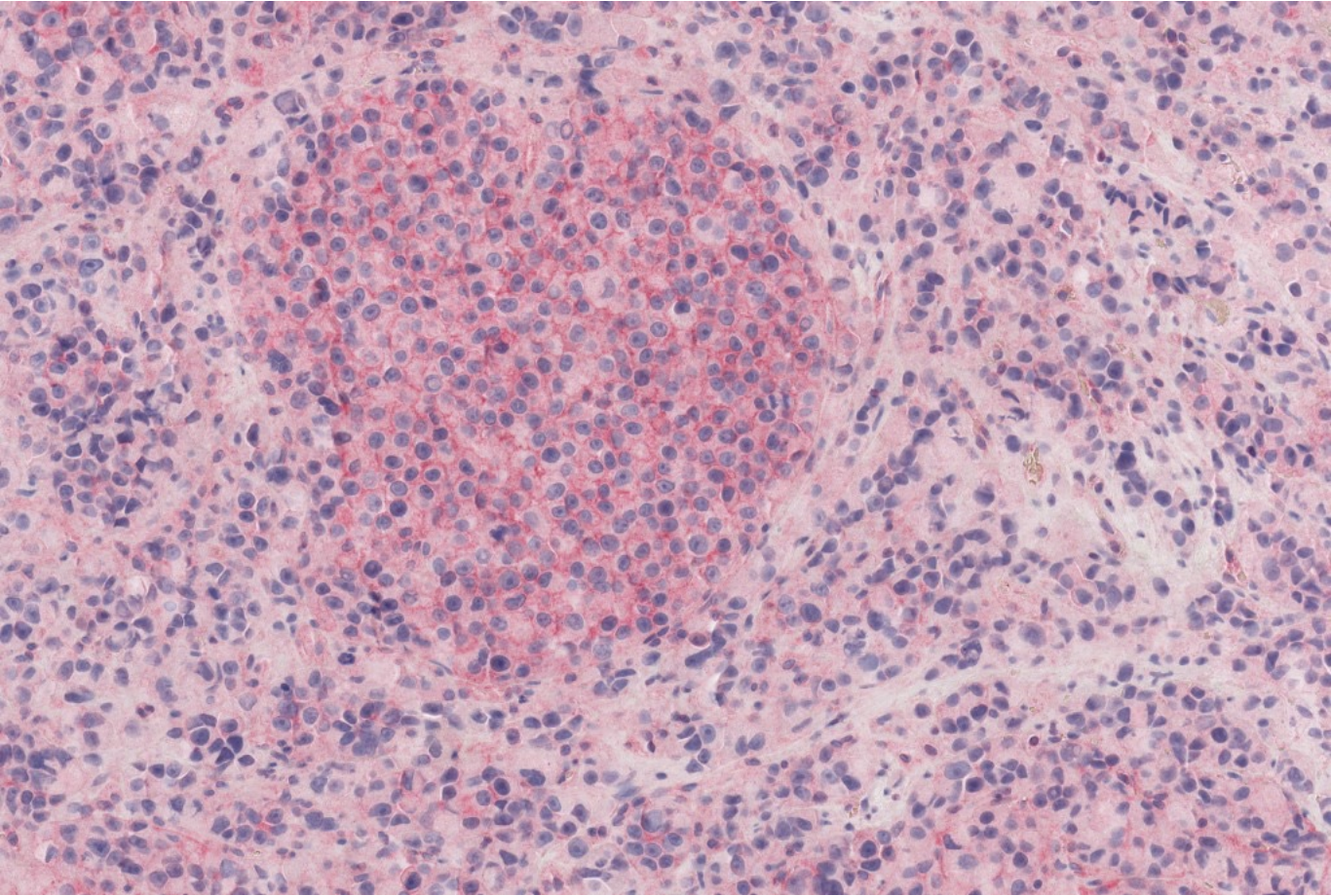

Supplement: Supplementary file 7 — Source Data for Figure 2 [file EMMM-15-e17719-s006.zip › Figure 2/2B/HUMSM #H17-11749.pdf]

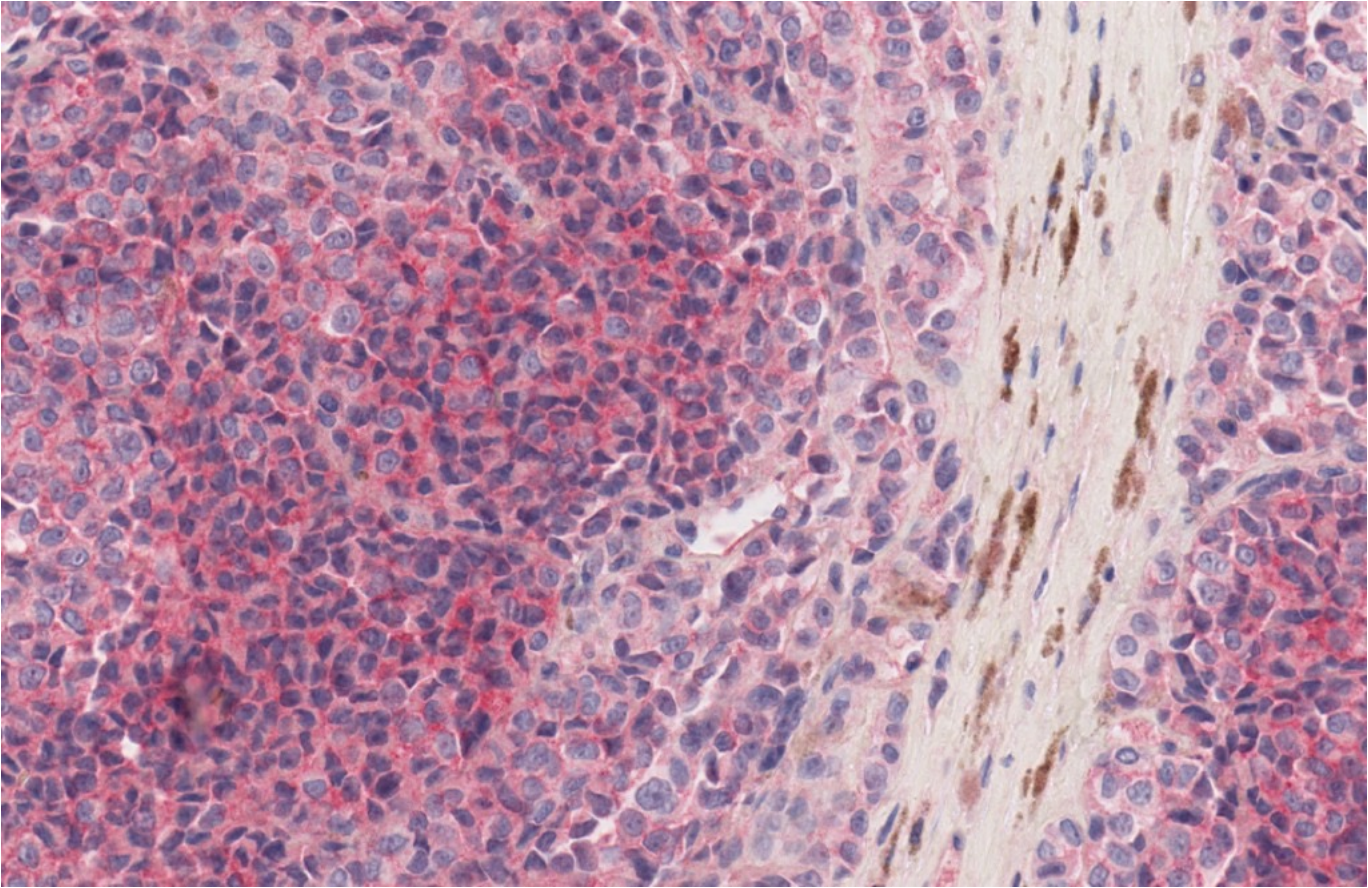

Supplement: Supplementary file 7 — Source Data for Figure 2 [file EMMM-15-e17719-s006.zip › Figure 2/2B/HUMSM #H17-20665.pdf]

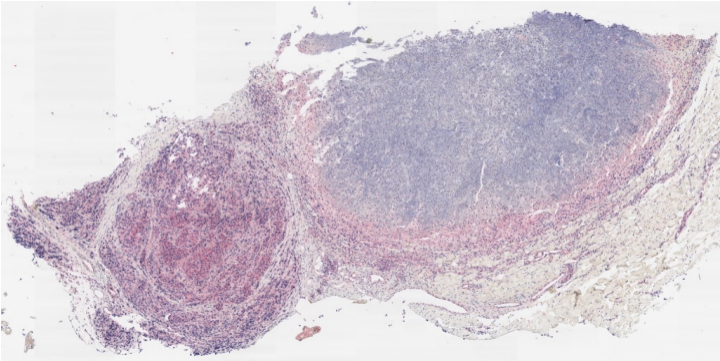

Supplement: Supplementary file 7 — Source Data for Figure 2 [file EMMM-15-e17719-s006.zip › Figure 2/2B/HUMSM #H17-12565.pdf]

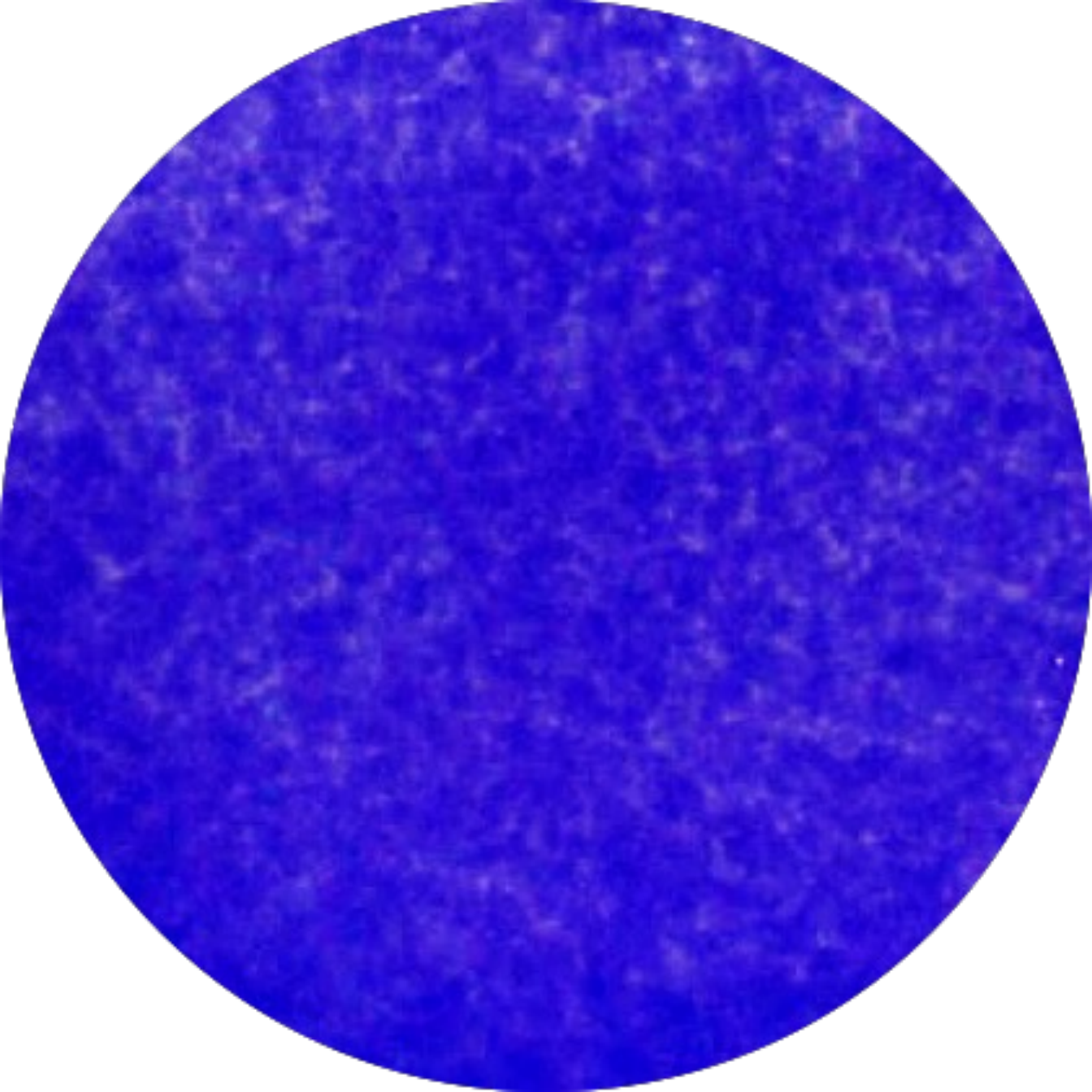

Supplement: Supplementary file 9 — Source Data for Figure 4 [file EMMM-15-e17719-s008.zip › Figure 4/4E/LKB1-KO1_siCtl.pdf]

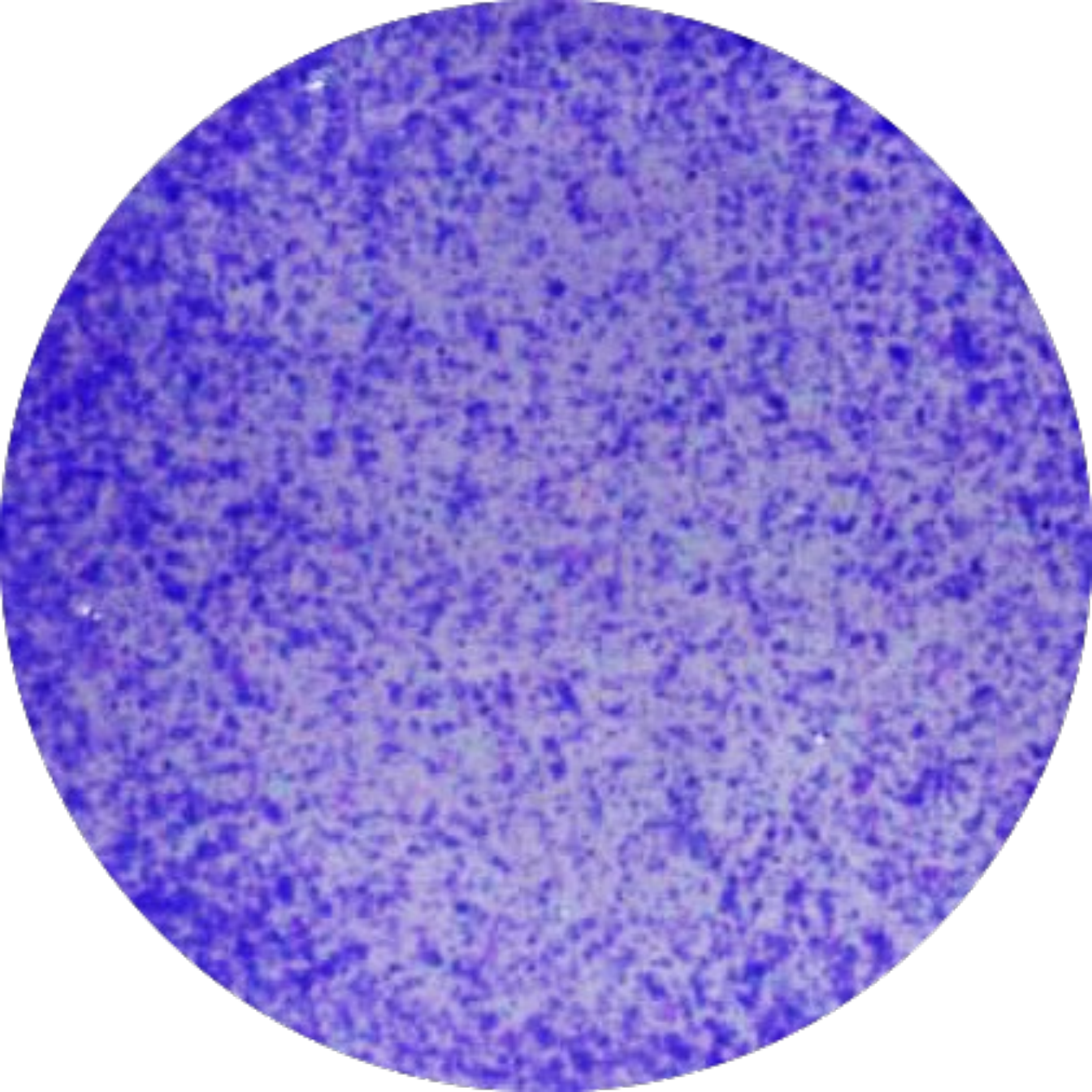

Supplement: Supplementary file 9 — Source Data for Figure 4 [file EMMM-15-e17719-s008.zip › Figure 4/4E/LKB1-KO1_siSLC8A1#1.pdf]

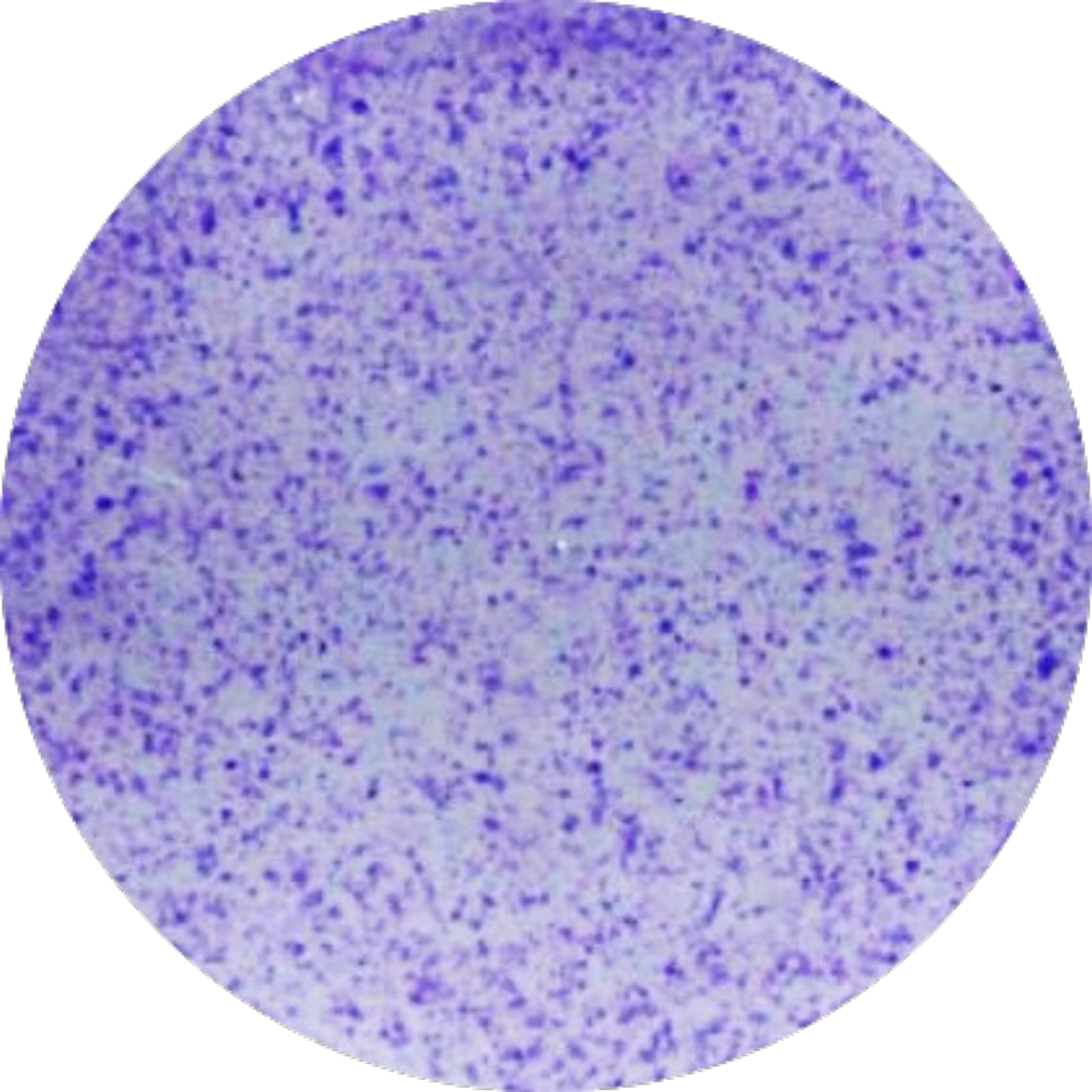

Supplement: Supplementary file 9 — Source Data for Figure 4 [file EMMM-15-e17719-s008.zip › Figure 4/4E/LKB1-KO1_siSLC8A1#2.pdf]

**Figure 4D**

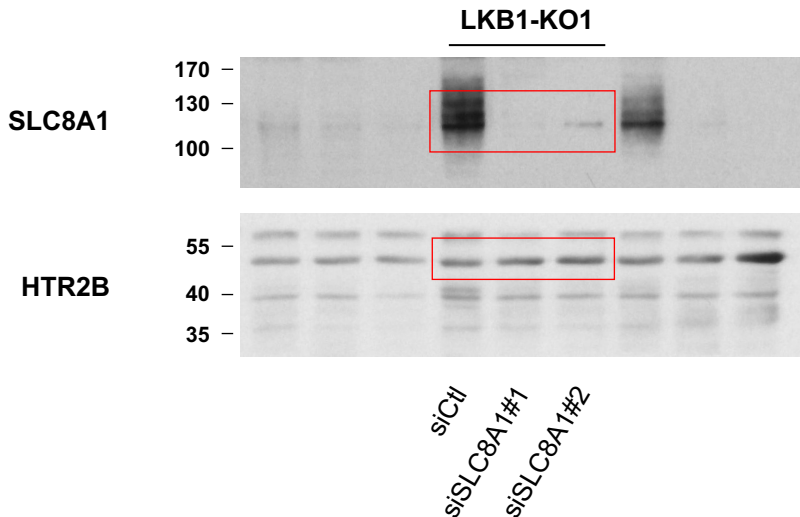

Supplement: Supplementary file 9 — Source Data for Figure 4 [file EMMM-15-e17719-s008.zip › Figure 4/4D/western SLC8A1_HTR2B.pdf]

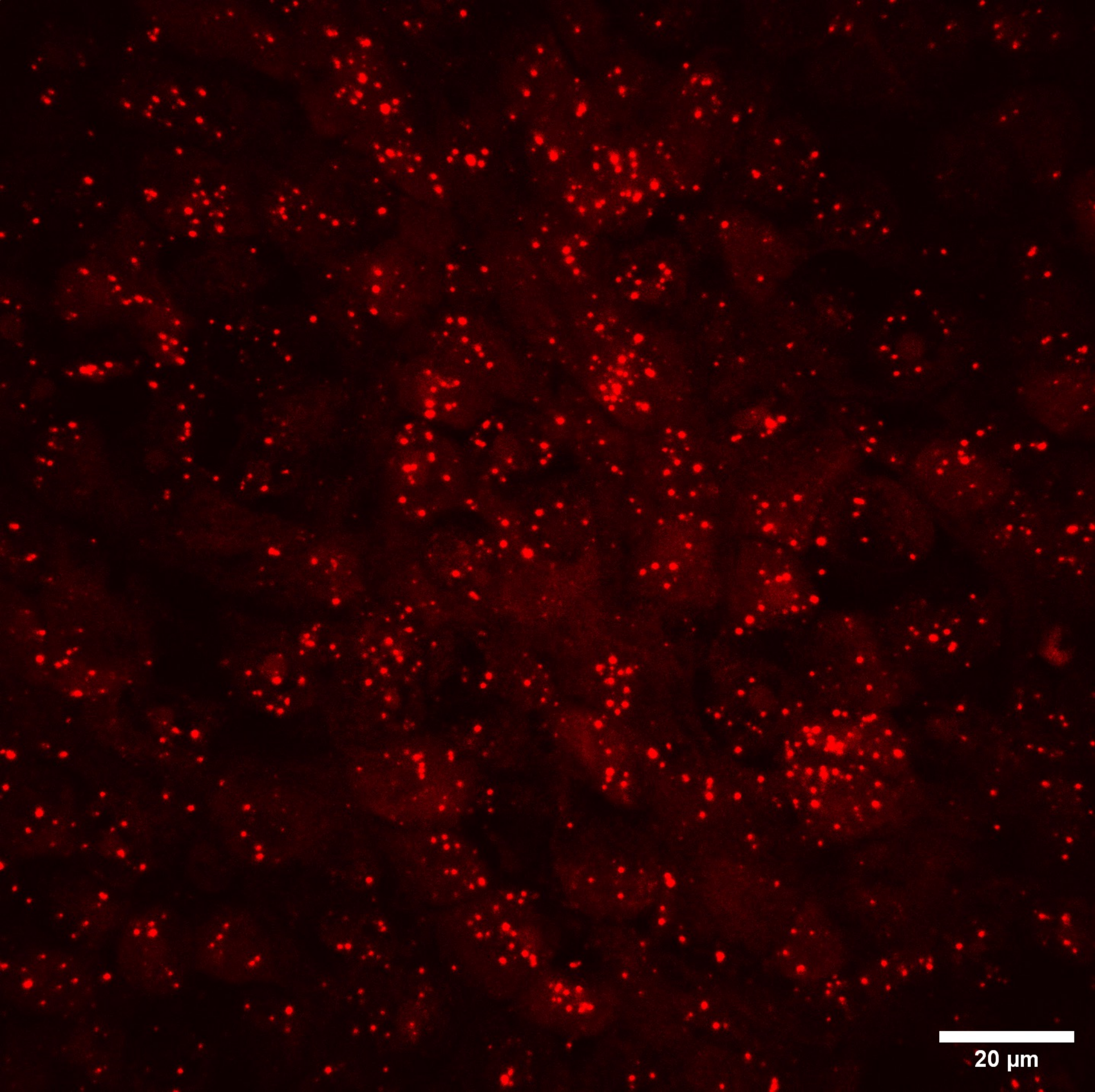

Supplement: Supplementary file 9 — Source Data for Figure 4 [file EMMM-15-e17719-s008.zip › Figure 4/4A/HUMSH17-11749 _BOTTOM_LEFT.pdf]

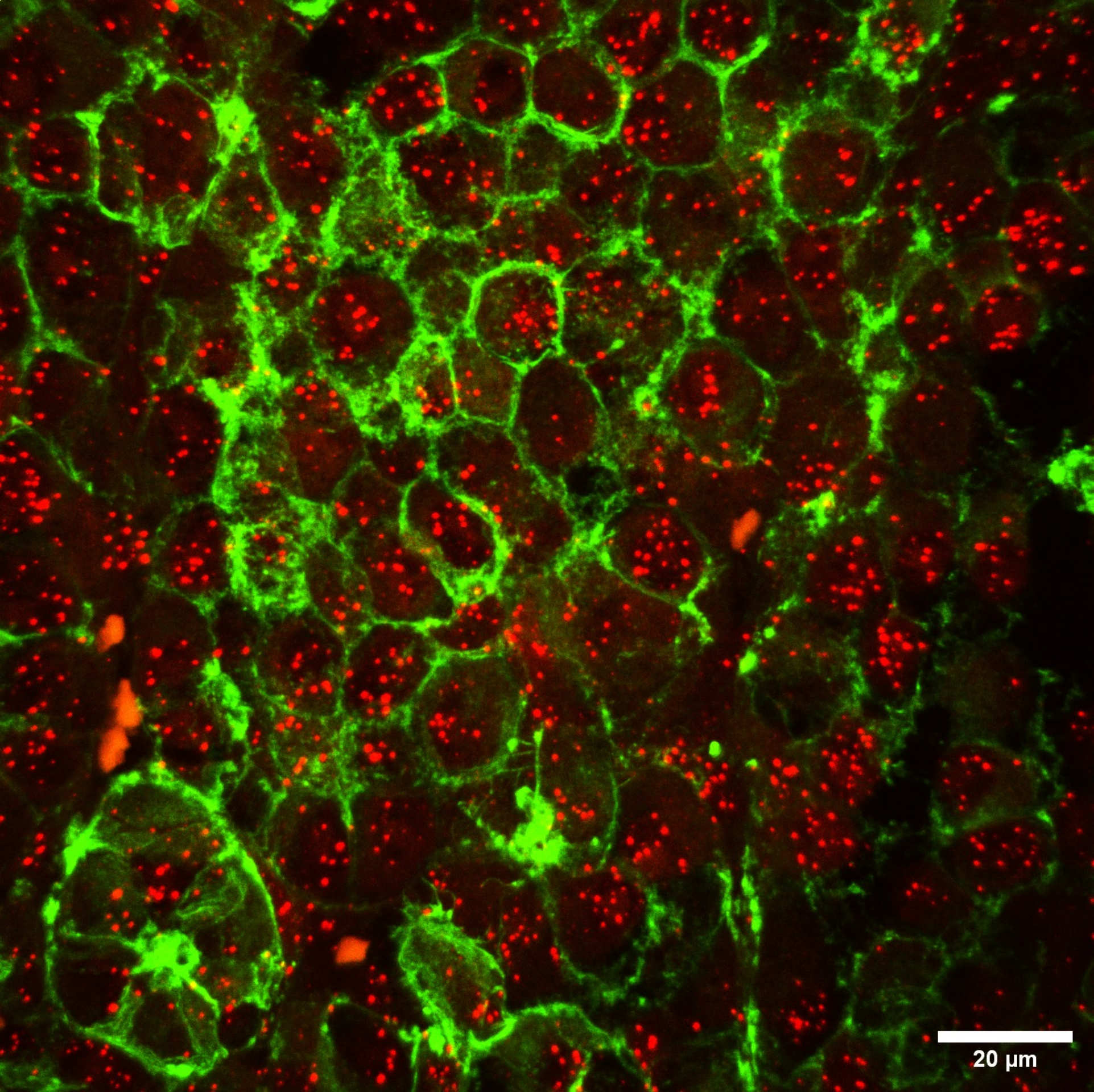

Supplement: Supplementary file 9 — Source Data for Figure 4 [file EMMM-15-e17719-s008.zip › Figure 4/4A/HUMSH17-11749 _TOP_RIGHT.pdf]

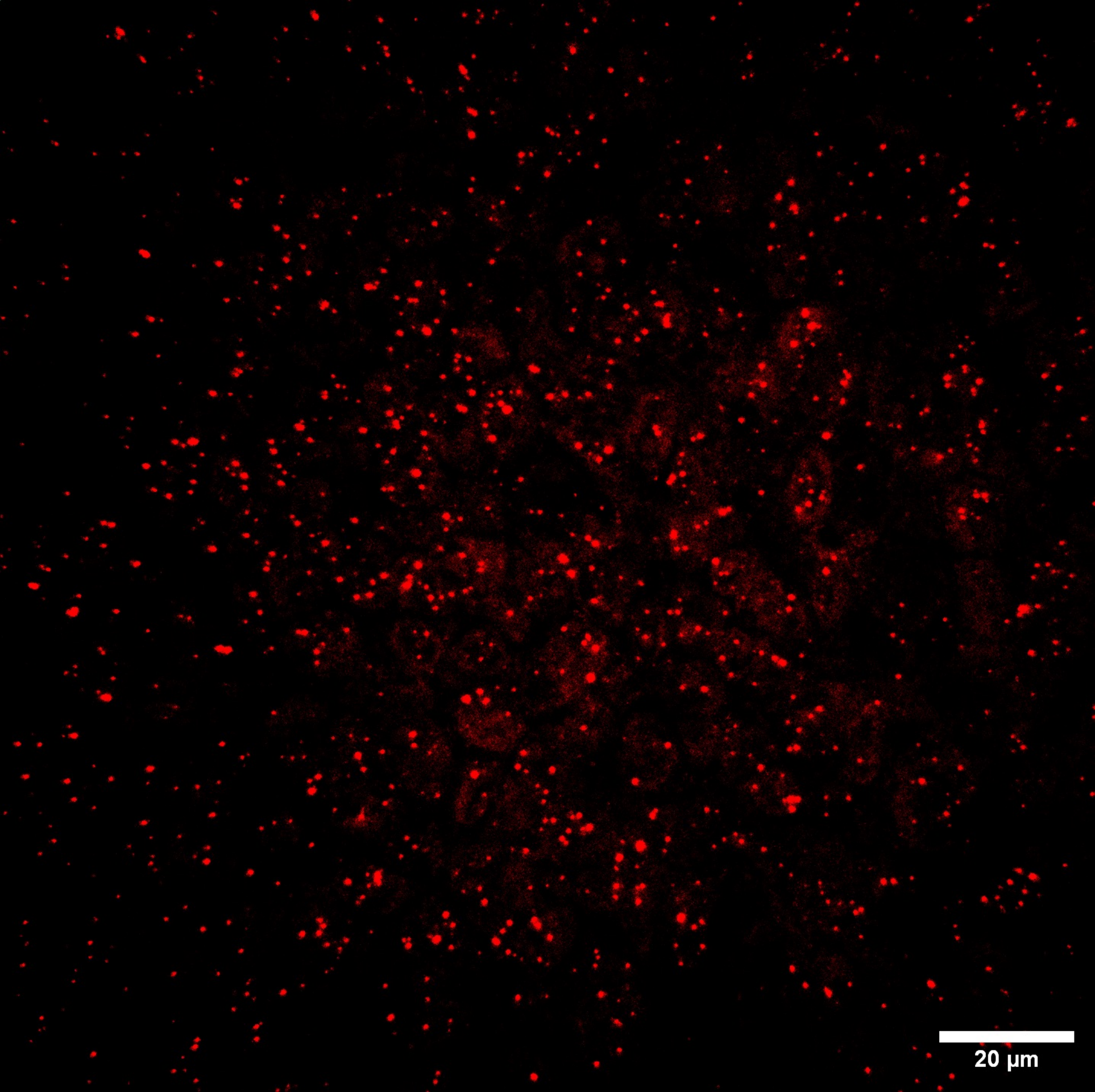

20 μm

Supplement: Supplementary file 9 — Source Data for Figure 4 [file EMMM-15-e17719-s008.zip › Figure 4/4A/HUMSM #H17-11747_BOTTOM_LEFT.pdf]

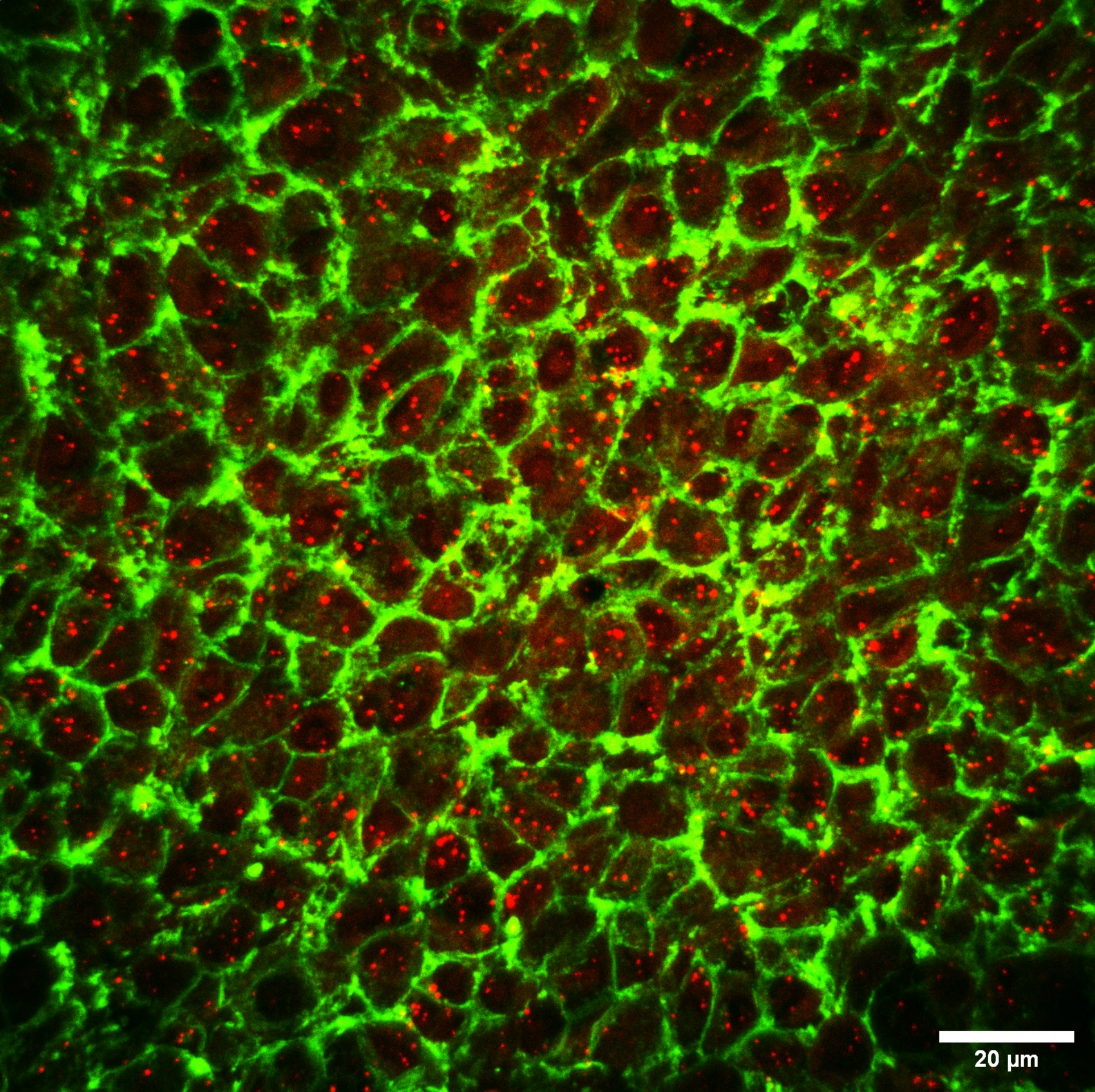

20  $\mu\text{m}$

Supplement: Supplementary file 9 — Source Data for Figure 4 [file EMMM-15-e17719-s008.zip › Figure 4/4A/HUMSM #H17-11747_TOP_RIGHT.pdf]

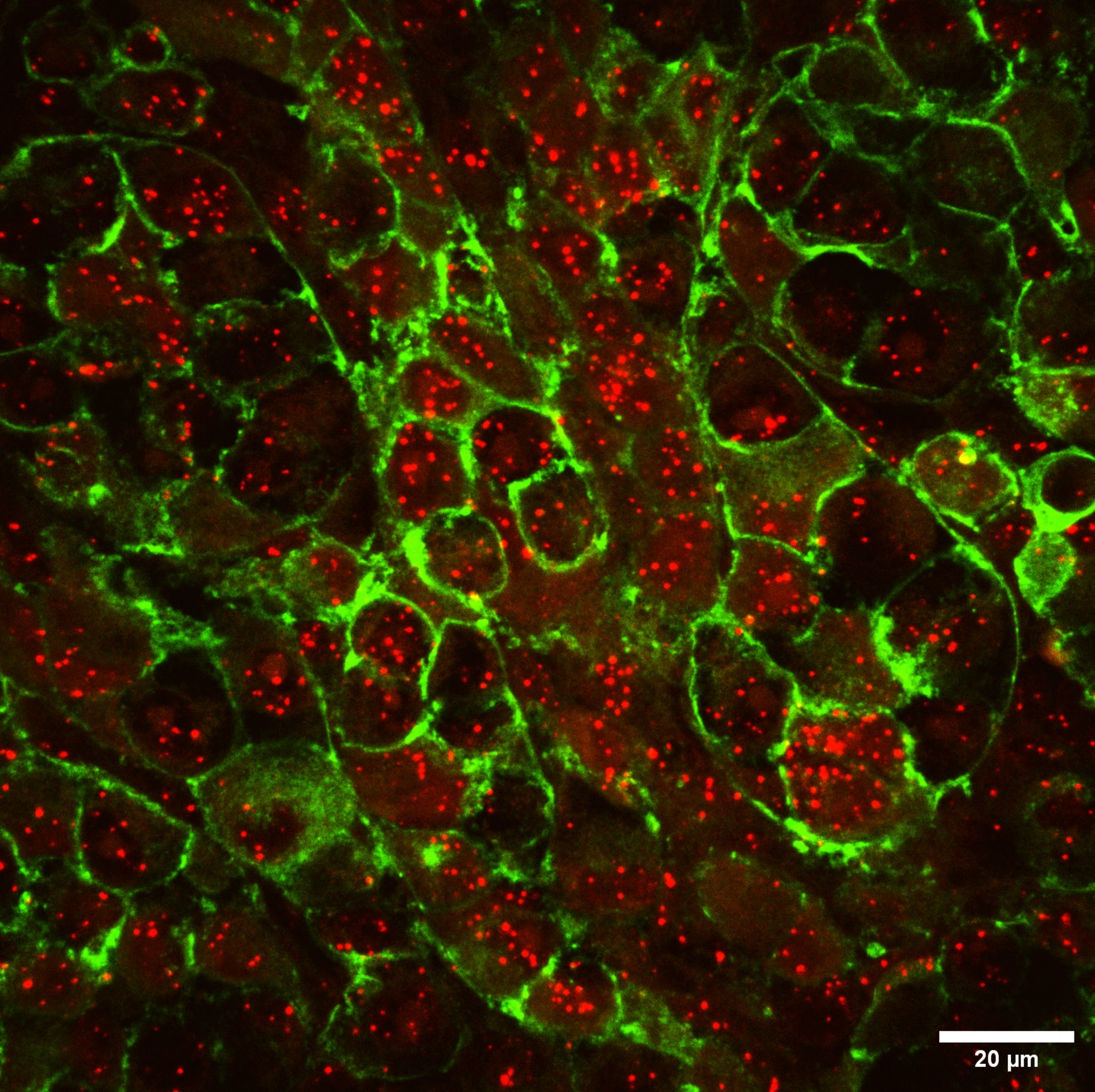

Supplement: Supplementary file 9 — Source Data for Figure 4 [file EMMM-15-e17719-s008.zip › Figure 4/4A/HUMSH17-11749 _TOP_LEFT.pdf]

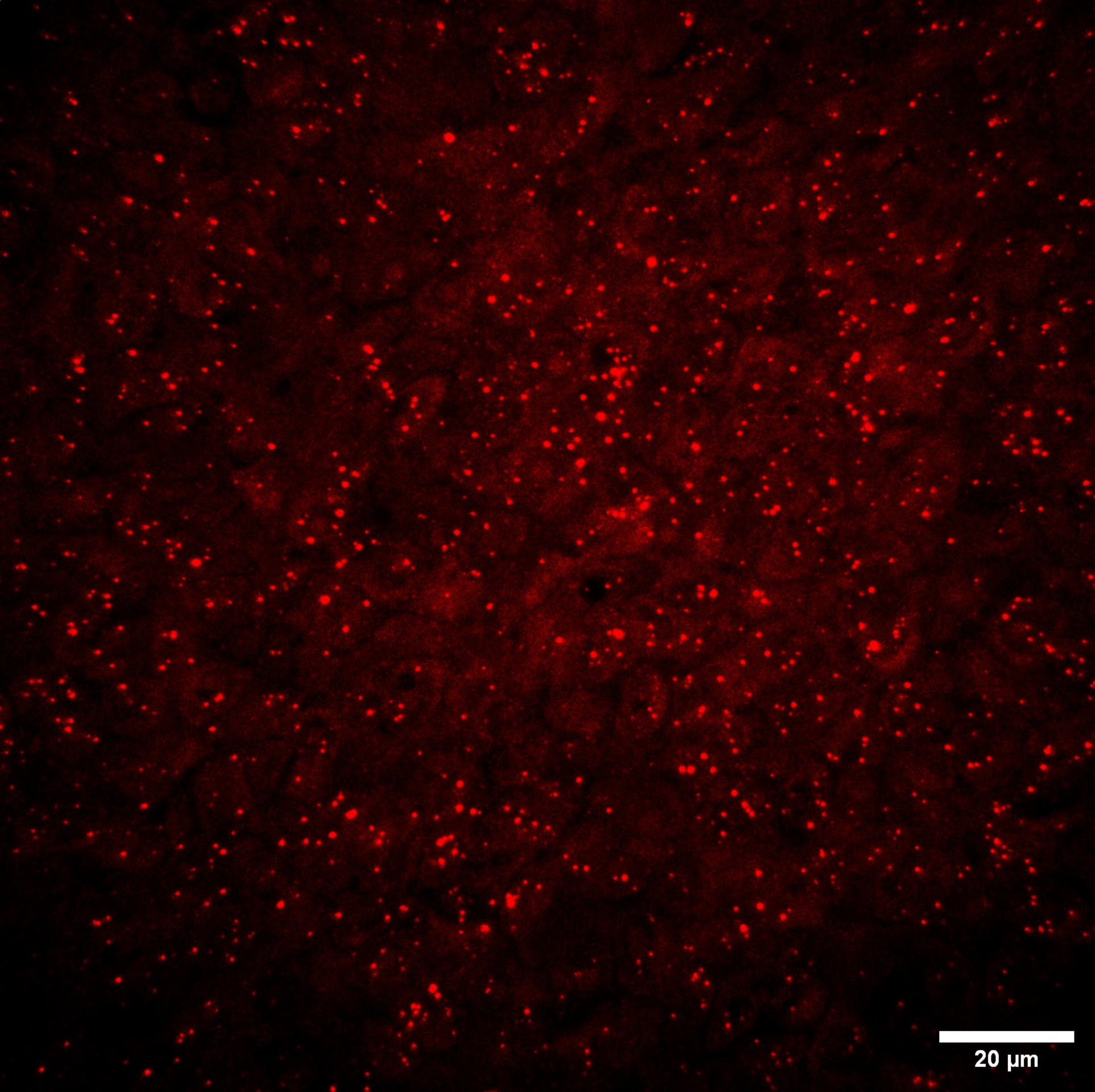

20 μm

Supplement: Supplementary file 9 — Source Data for Figure 4 [file EMMM-15-e17719-s008.zip › Figure 4/4A/HUMSM #H17-11747_BOTTOM_RIGHT.pdf]

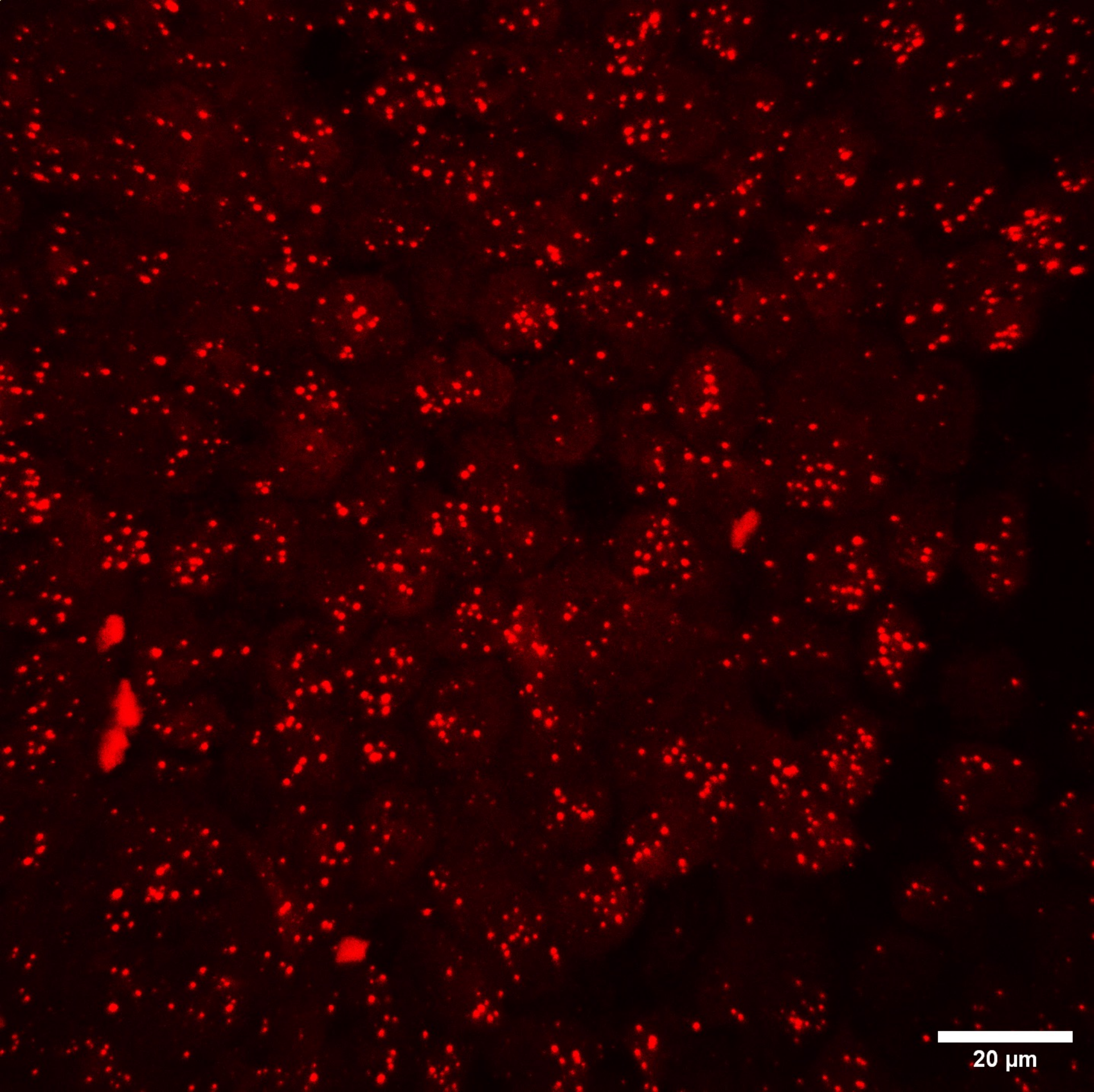

Supplement: Supplementary file 9 — Source Data for Figure 4 [file EMMM-15-e17719-s008.zip › Figure 4/4A/HUMSH17-11749 _BOTTOM_RIGHT.pdf]

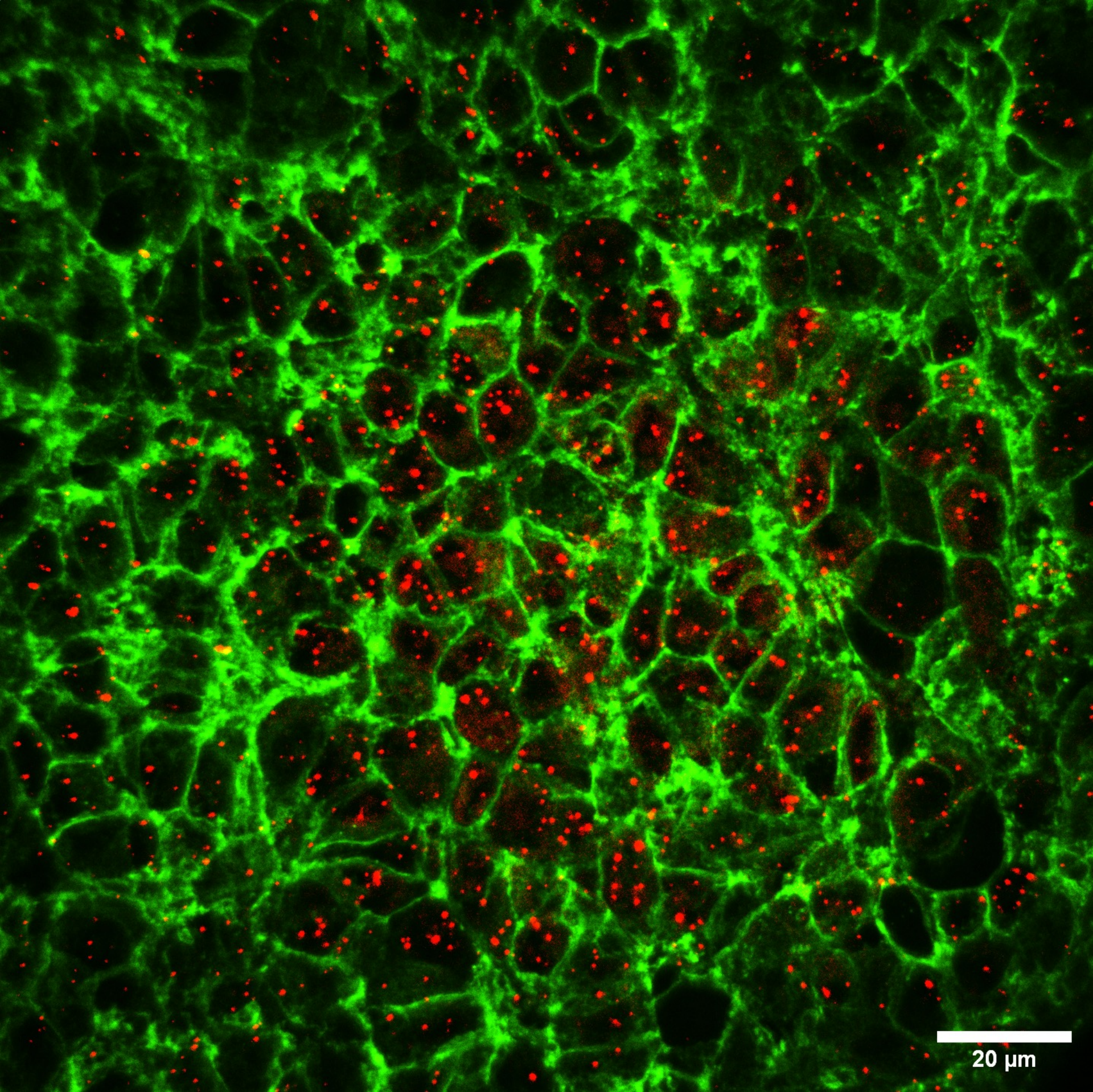

20  $\mu\text{m}$

Supplement: Supplementary file 9 — Source Data for Figure 4 [file EMMM-15-e17719-s008.zip › Figure 4/4A/HUMSM #H17-11747_TOP_LEFT.pdf]

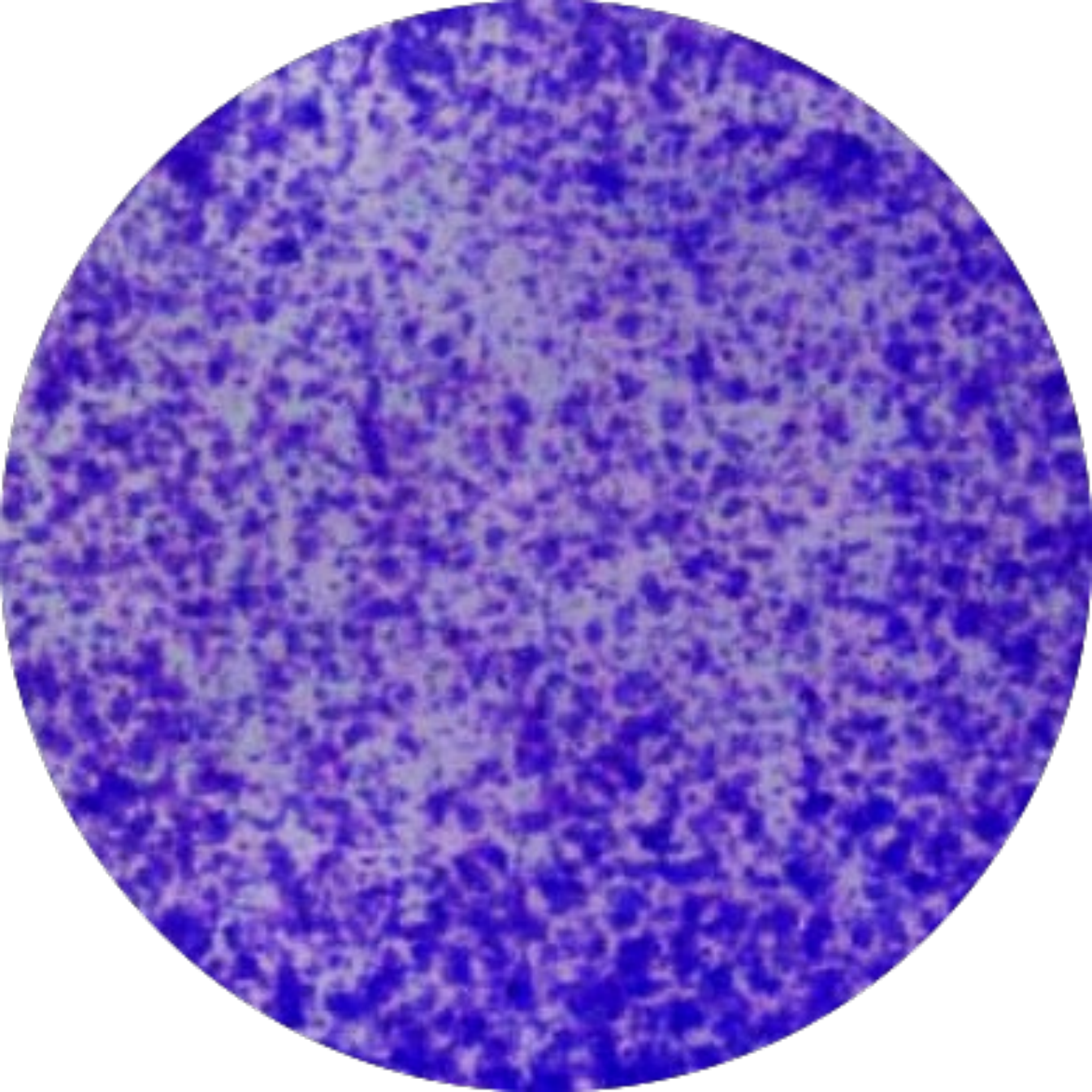

Supplement: Supplementary file 10 — Source Data for Figure 5 [file EMMM-15-e17719-s003.zip › Figure 5/5G/SIK2-KO1_siCtl.pdf]

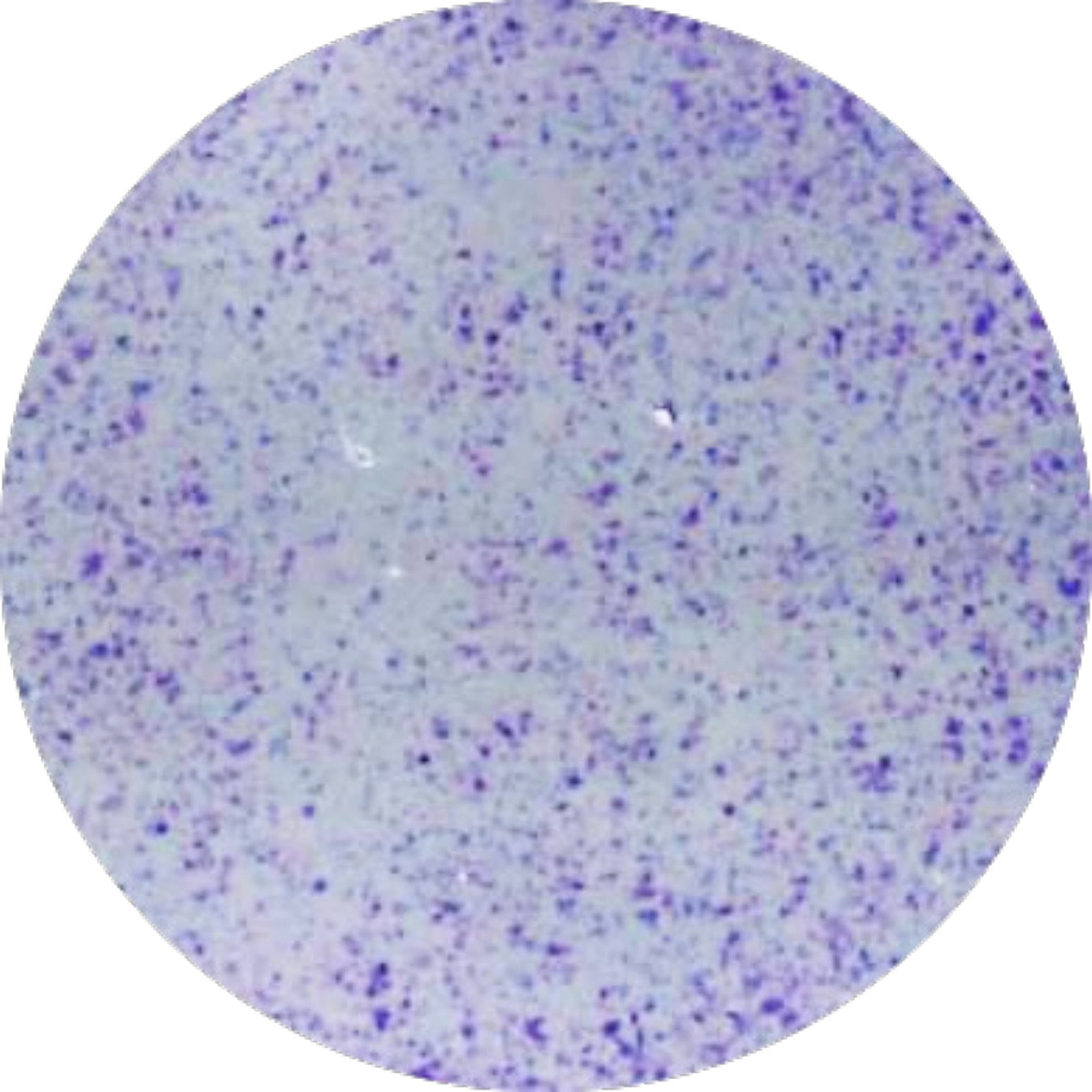

Supplement: Supplementary file 10 — Source Data for Figure 5 [file EMMM-15-e17719-s003.zip › Figure 5/5G/SIK2-KO1_siSLC8A1#1.pdf]

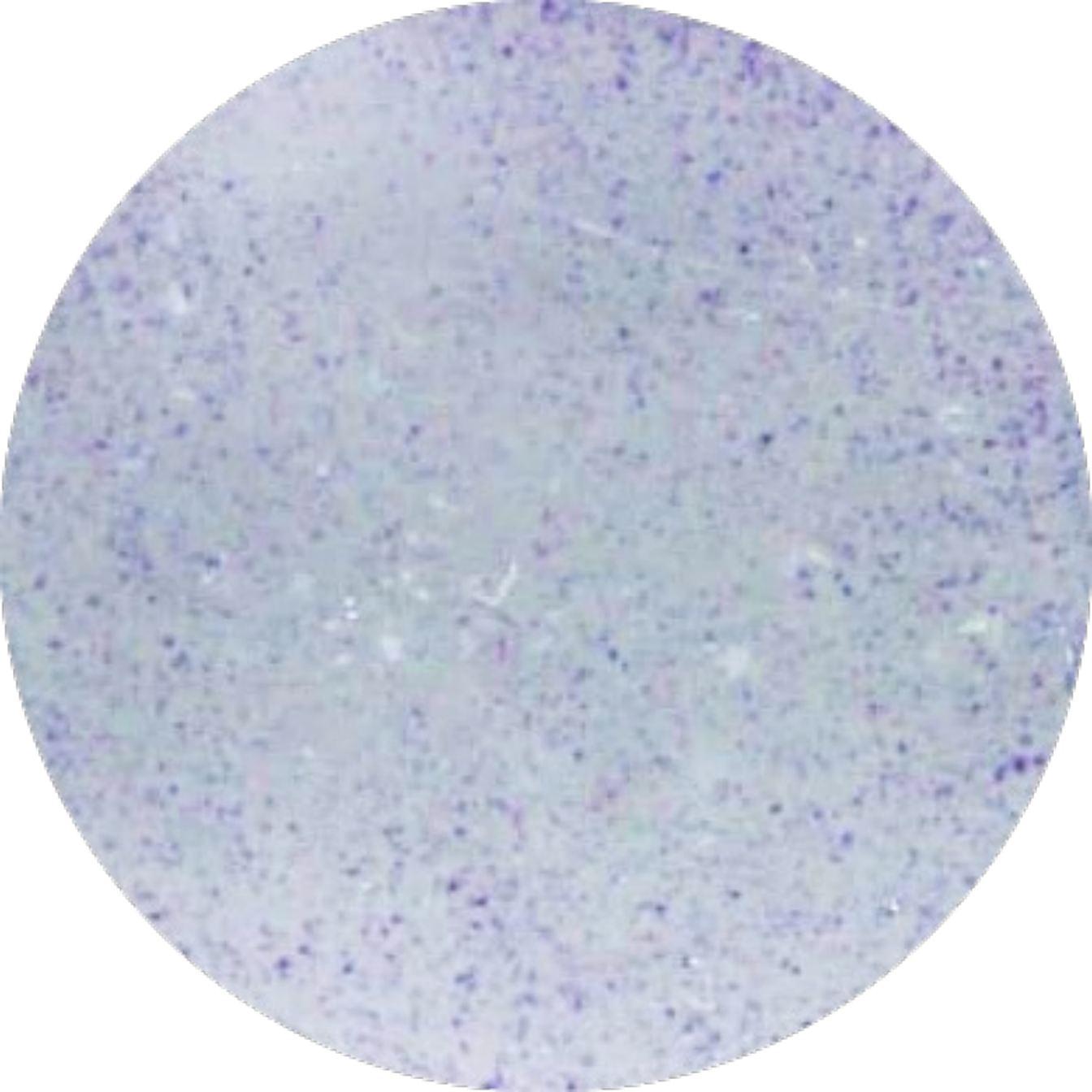

Supplement: Supplementary file 10 — Source Data for Figure 5 [file EMMM-15-e17719-s003.zip › Figure 5/5G/SIK2-KO1_siSLC8A1#2.pdf]

**Figure 5A**

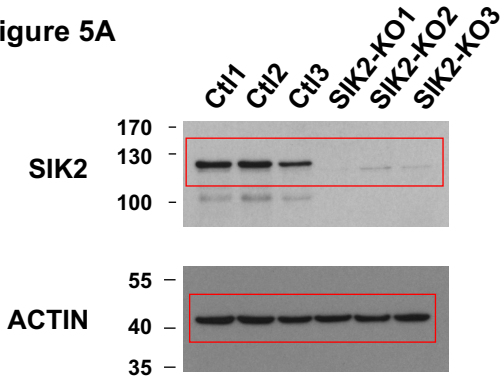

Supplement: Supplementary file 10 — Source Data for Figure 5 [file EMMM-15-e17719-s003.zip › Figure 5/5A/western SIK2_ACTIN.pdf]

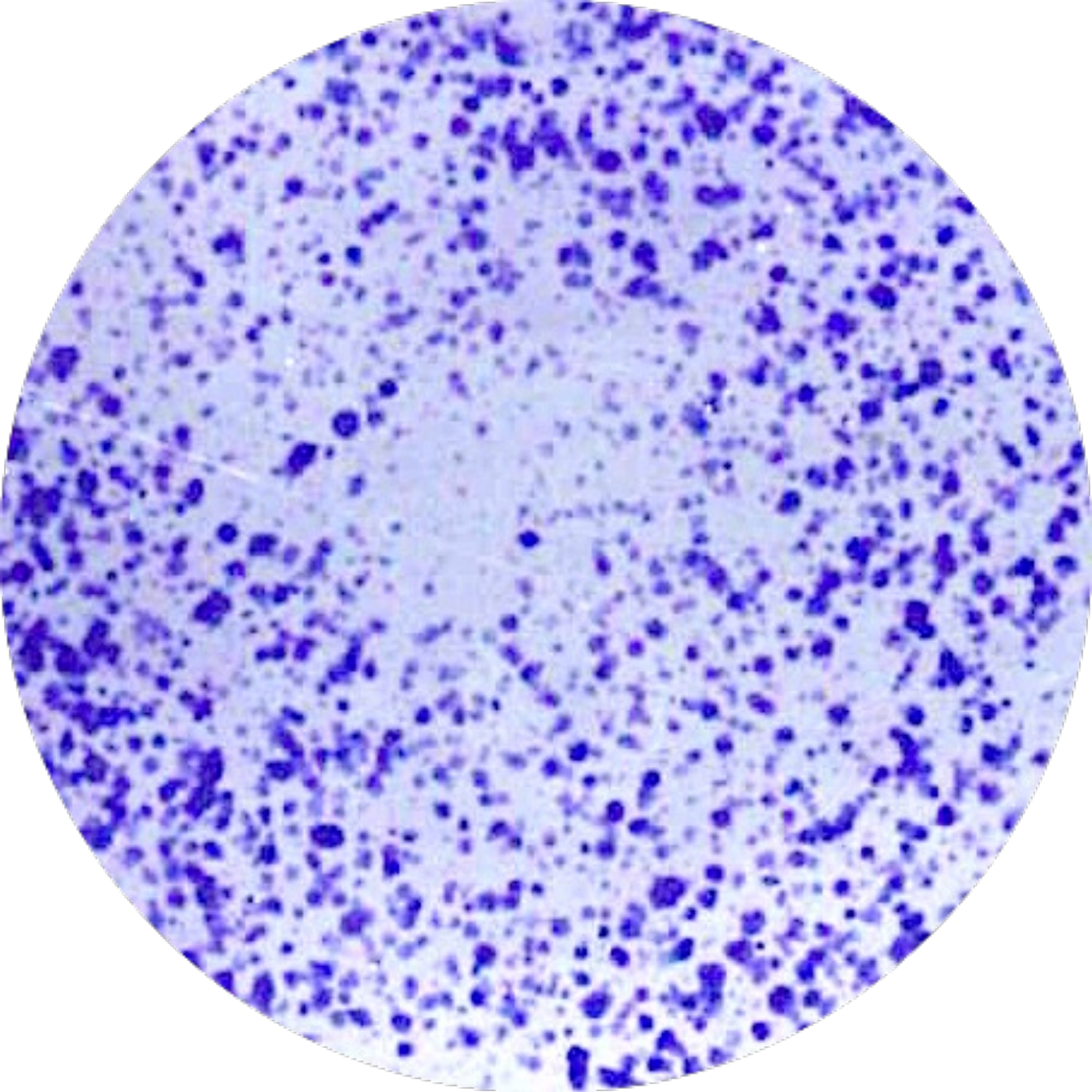

Supplement: Supplementary file 10 — Source Data for Figure 5 [file EMMM-15-e17719-s003.zip › Figure 5/5C/SIK2T175D.pdf]

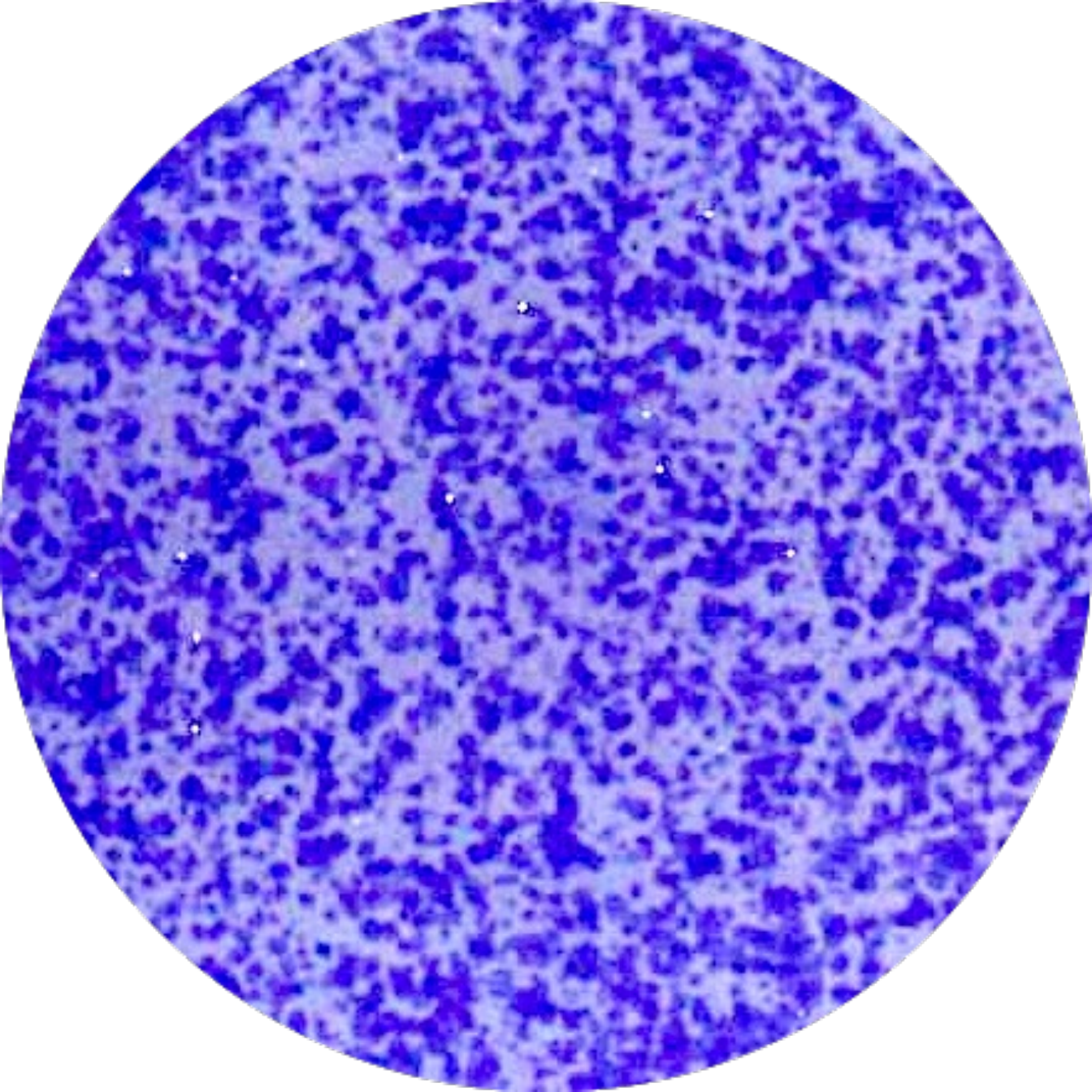

Supplement: Supplementary file 10 — Source Data for Figure 5 [file EMMM-15-e17719-s003.zip › Figure 5/5C/SIK2K49M.pdf]

**Figure 5C**

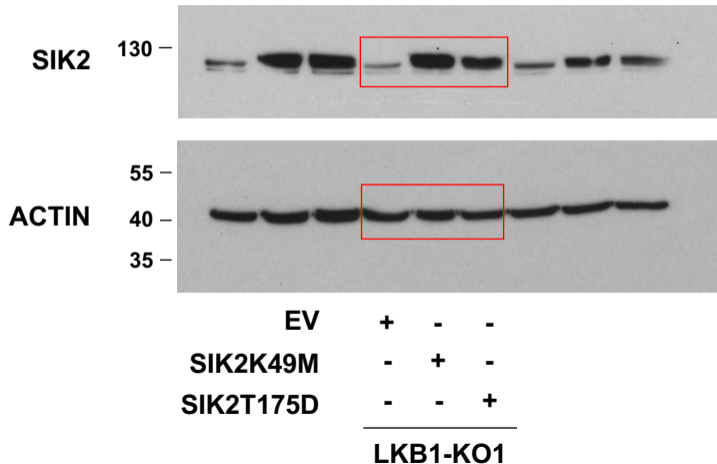

Supplement: Supplementary file 10 — Source Data for Figure 5 [file EMMM-15-e17719-s003.zip › Figure 5/5C/western SIK2_ACTIN.pdf]

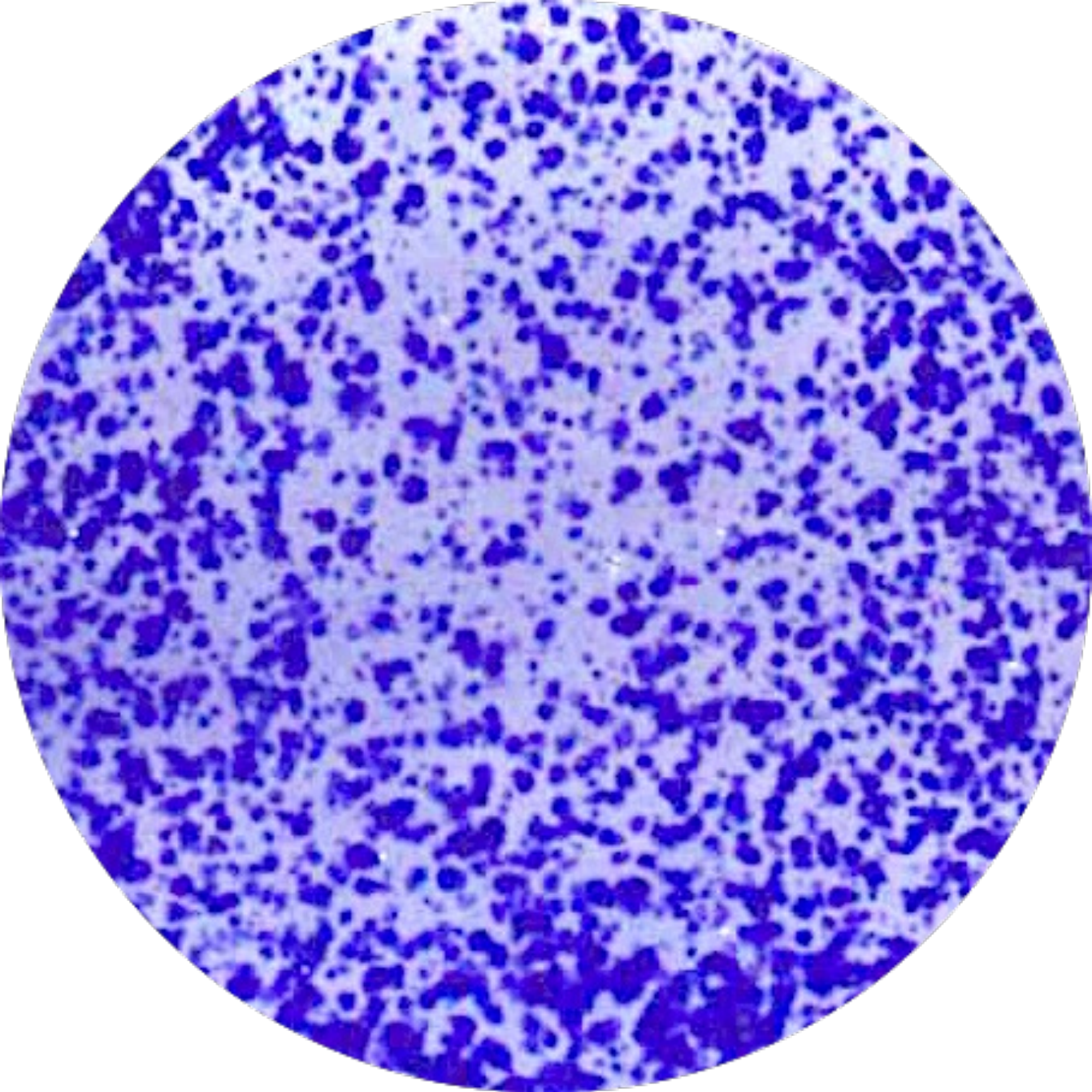

Supplement: Supplementary file 10 — Source Data for Figure 5 [file EMMM-15-e17719-s003.zip › Figure 5/5C/EV.pdf]

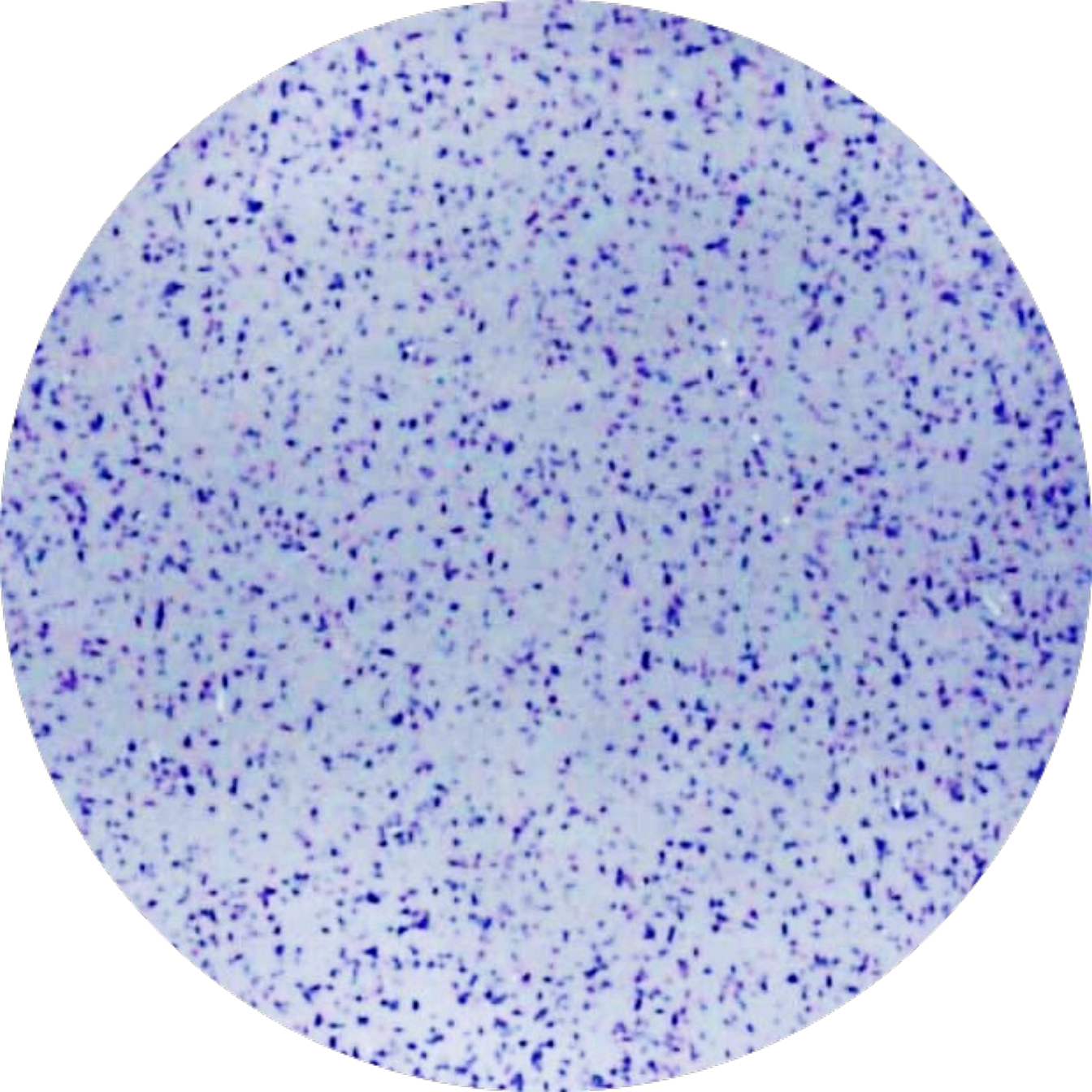

Supplement: Supplementary file 10 — Source Data for Figure 5 [file EMMM-15-e17719-s003.zip › Figure 5/5B/Ctl3.pdf]

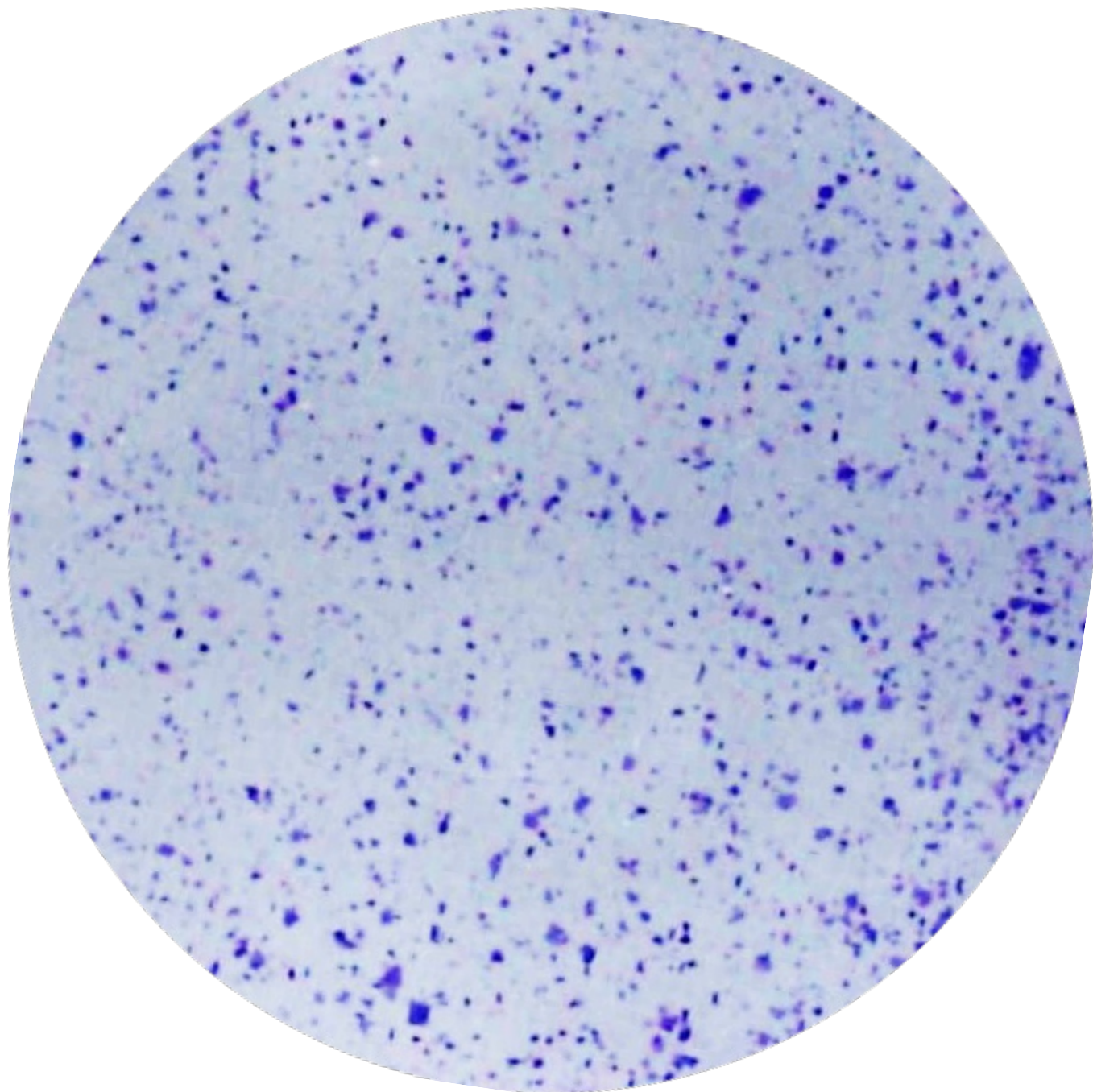

Supplement: Supplementary file 10 — Source Data for Figure 5 [file EMMM-15-e17719-s003.zip › Figure 5/5B/Ctl2.pdf]

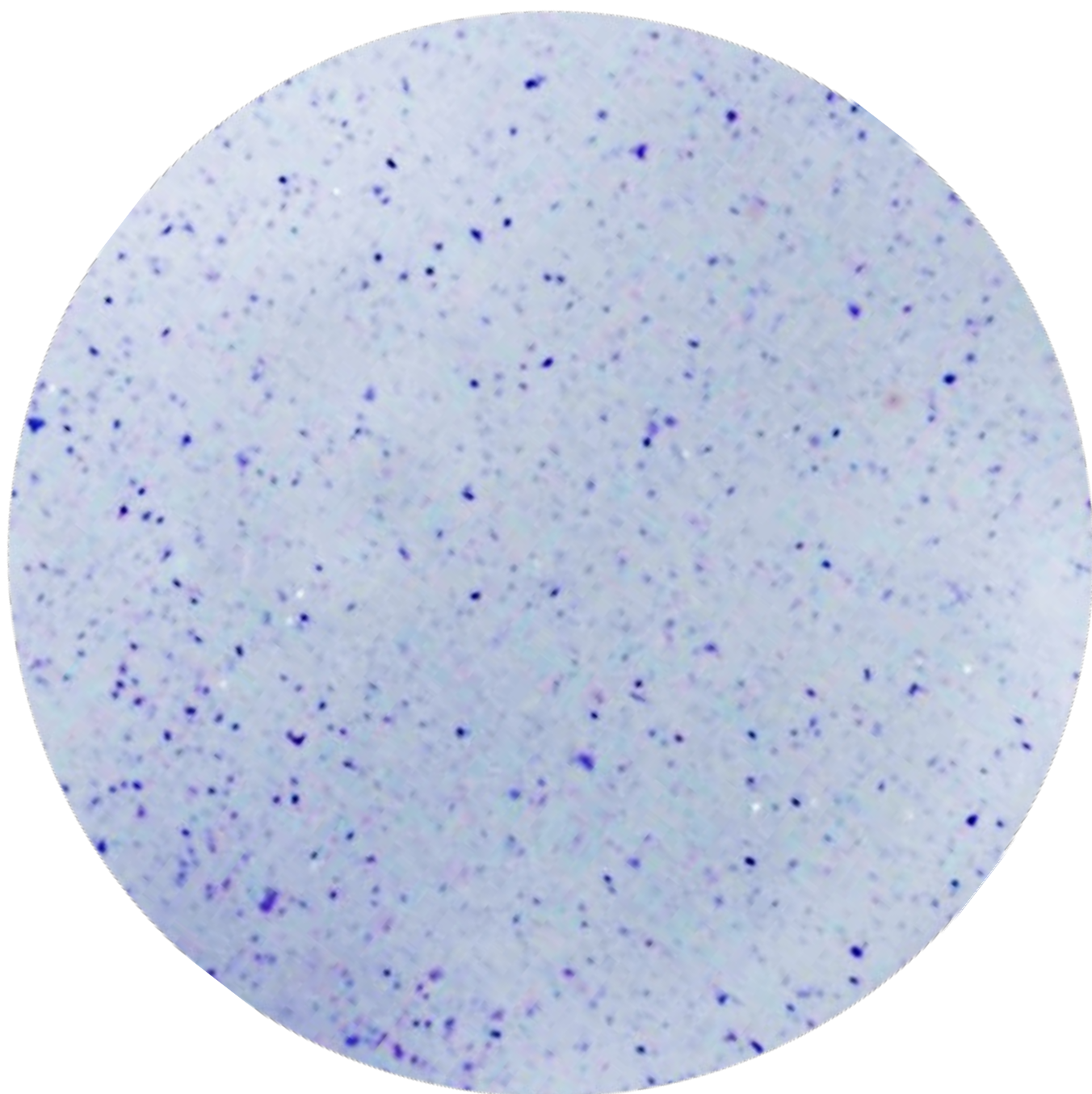

Supplement: Supplementary file 10 — Source Data for Figure 5 [file EMMM-15-e17719-s003.zip › Figure 5/5B/Ctl1.pdf]

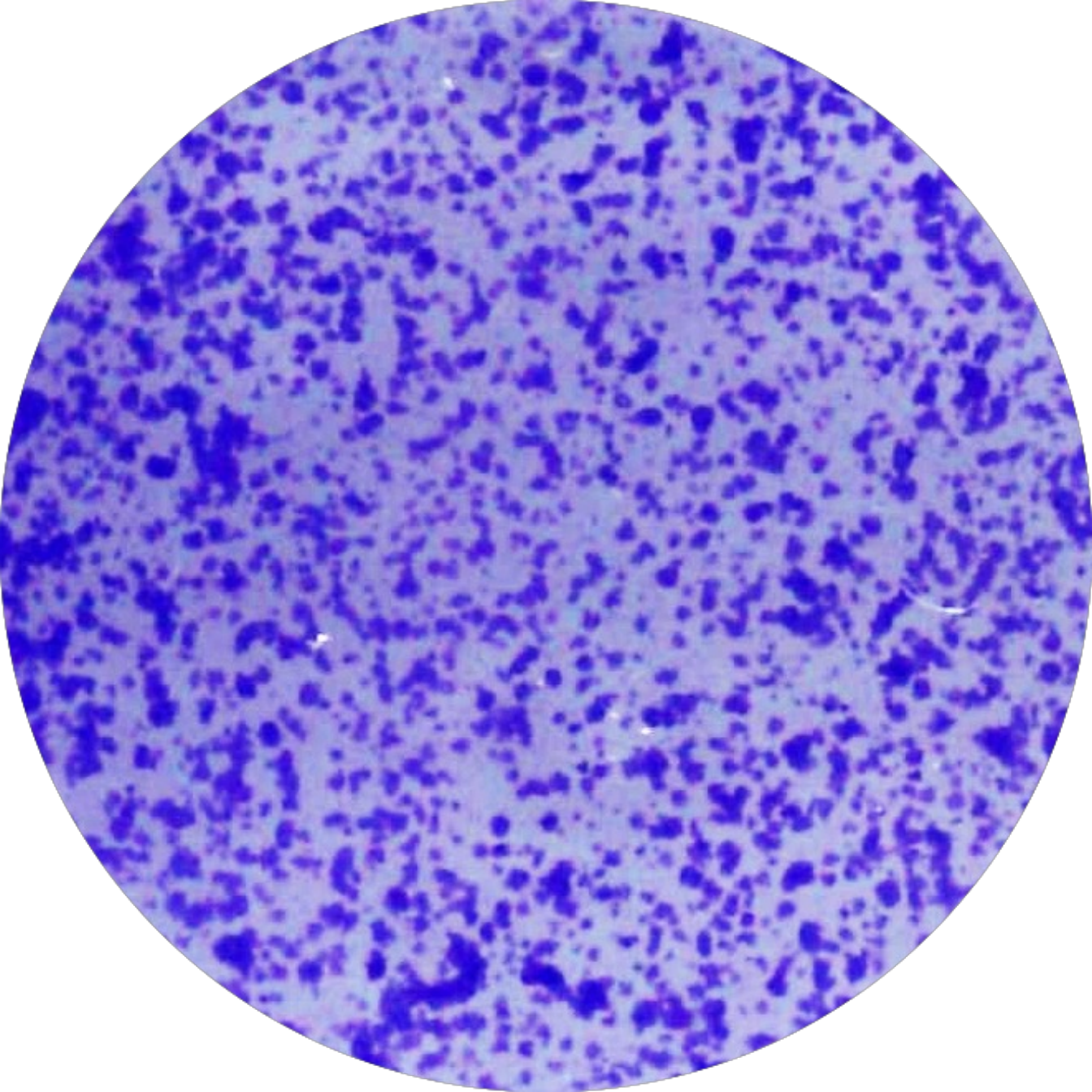

Supplement: Supplementary file 10 — Source Data for Figure 5 [file EMMM-15-e17719-s003.zip › Figure 5/5B/SIK2-KO2.pdf]

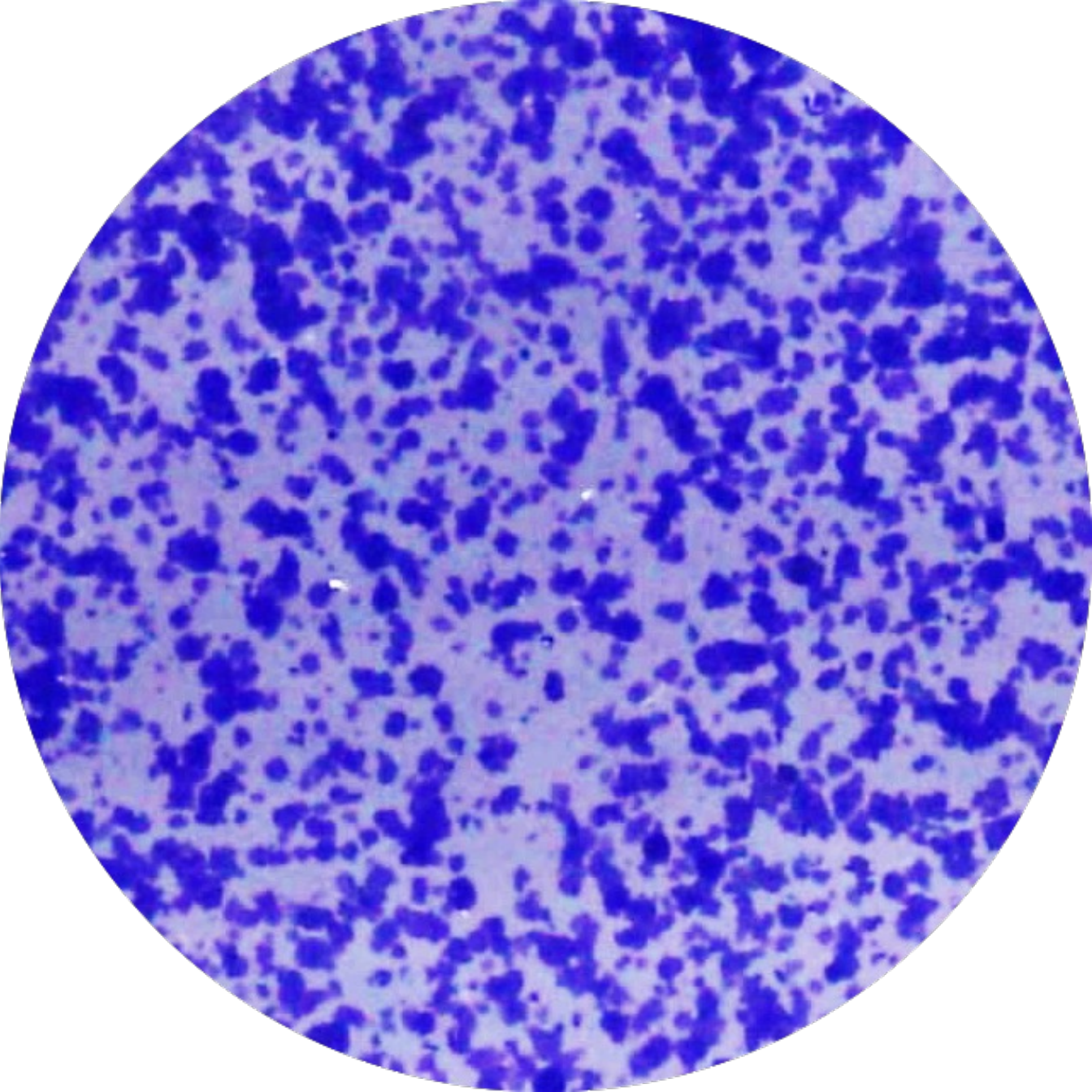

Supplement: Supplementary file 10 — Source Data for Figure 5 [file EMMM-15-e17719-s003.zip › Figure 5/5B/SIK2-KO3.pdf]

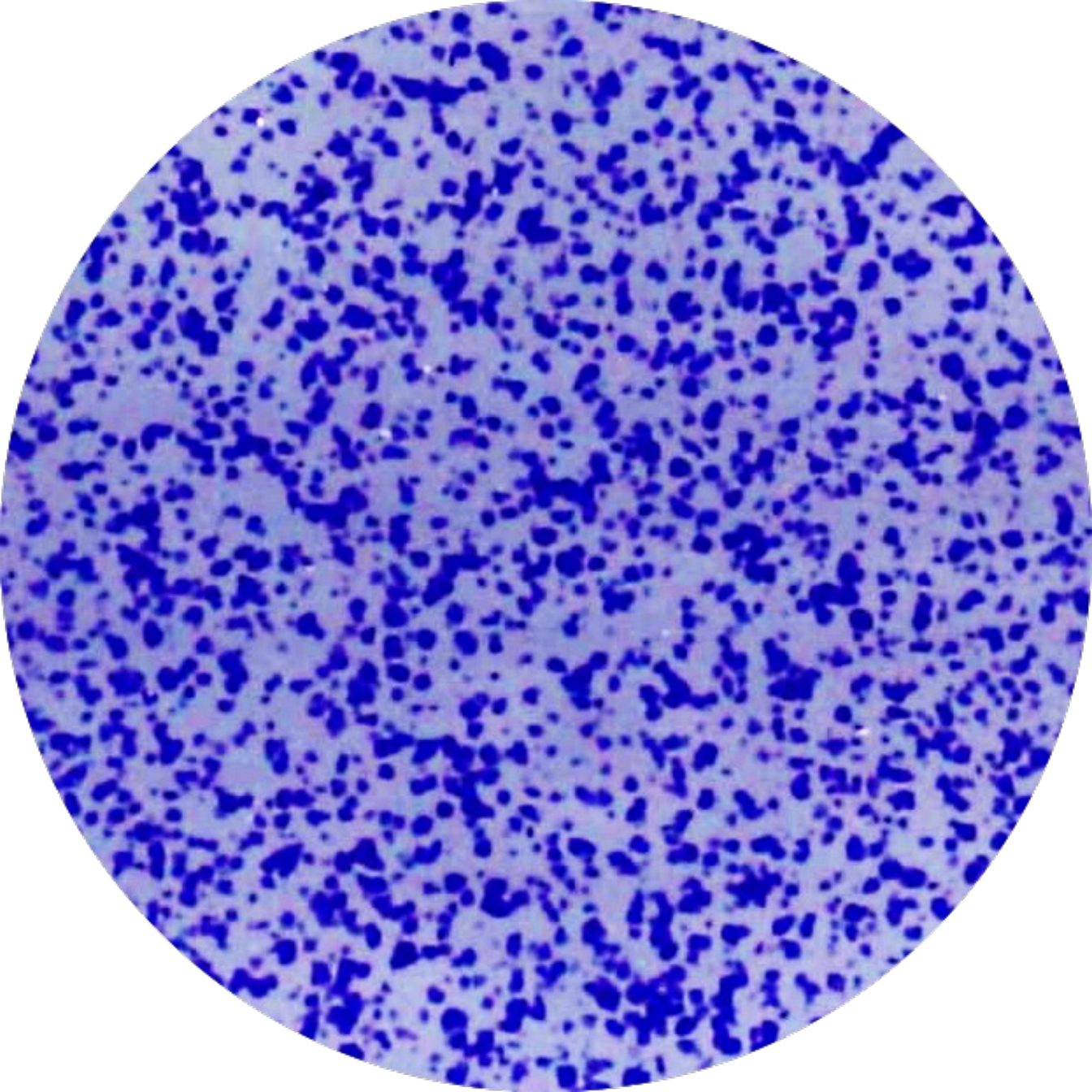

Supplement: Supplementary file 10 — Source Data for Figure 5 [file EMMM-15-e17719-s003.zip › Figure 5/5B/SIK2-KO1.pdf]

**Figure 7B**

**Vehicle**

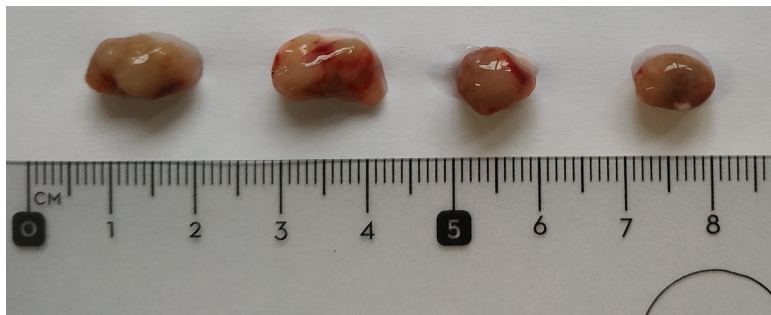

**KB-R7943**

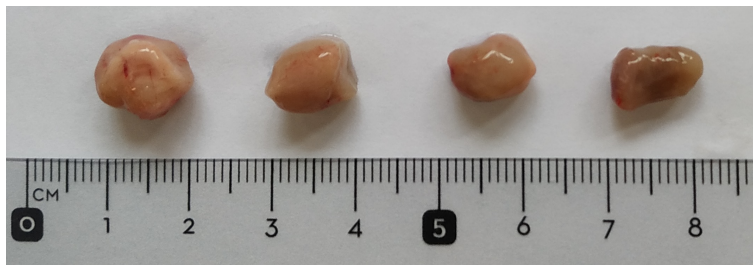

**MitoQ**

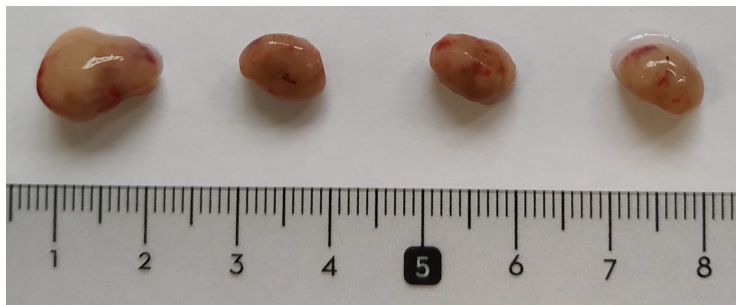

**MitoQ+KB-R7943**

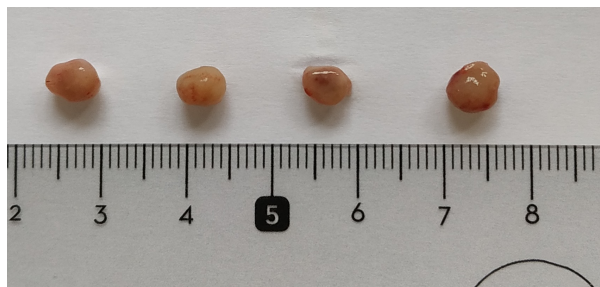

Supplement: Supplementary file 12 — Source Data for Figure 7 [file EMMM-15-e17719-s005.zip › Figure 7/7B/tumors picture.pdf]
