# Supplementary material for: LKB1‐SIK2 loss drives uveal melanoma proliferation and hypersensitivity to SLC8A1 and ROS inhibition
Source: EMBO Mol Med. 2023 Nov 15;15(12):e17719. doi: 10.15252/emmm.202317719 (PMC10701601; doi:10.15252/emmm.202317719)
Supplement: Supplementary file 13 [file EMMM-15-e17719-s001.pdf]

EMBO Molecular Medicine

LKB1-SIK2 loss drives uveal melanoma proliferation and hypersensitivity to SLC8A1 and ROS inhibition

Thomas Strub, Sarah Hireche-Proteau, Imène Krossa, Chrystel Husser, Maxime GUEGUINOU, Federica Sella, Karine Bille, Marie Irondelle, Mélanie Dalmasso, Thibault Barouillet, Yann Cheli, Céline Pisibon, Nicole Arrighi, Sacha Nahon-Estève, Arnaud Martel, Lauris Gastaud, Sandra Lassalle, Olivier Mignen, patrick brest, Nathalie Mazure, Frederic Bost, Stéphanie Baillif, Solange Landreville, Simon Turcotte, Dan Hasson, Saul Carcamo, Christophe Vandier, Emily Bernstein, Laurent Yvan-Charvet, Mitchell Levesque, Robert Ballotti, and Corine Bertolotto
DOI: 10.15252/emmm.202317719

Corresponding authors: Thomas Strub (Thomas.STRUB@univ-cotedazur.fr) , Corine Bertolotto (bertolot@unice.fr)

Review Timeline:

Submission Date:	17th Mar 23
Editorial Decision:	27th Apr 23
Revision Received:	13th Jul 23
Editorial Decision:	10th Aug 23
Authors' Correspondence:	3rd Sep 23
Editor's Correspondence	8th Sep 23
Revision Received:	19th Sep 23
Editorial Decision:	6th Oct 23
Revision Received:	16th Oct 23
Accepted:	23rd Oct 23

Editor: Lise Roth

Transaction Report:

(Note: With the exception of the correction of typographical or spelling errors that could be a source of ambiguity, letters and reports are not edited. Depending on transfer agreements, referee reports obtained elsewhere may or may not be included in this compilation. Referee reports are anonymous unless the Referee chooses to sign their reports.)

27th Apr 2023

Dear Dr. Strub,

Thank you for the submission of your manuscript to EMBO Molecular Medicine, and please accept my apologies for the delay in getting back to you as we were waiting for one more referee report. However, given that referee #2 has not yet gotten back to us despite several chasers, and that both referees #1 and #3 provide similar recommendations, we prefer to make a decision now in order to avoid further delay in the process. Should referee #2 provide a report, we will send it to you, with the understanding that we will not ask you extensive experiments in addition to the ones required in the enclosed reports from referees #1 and #3.

As you will see from the reports below, both referees acknowledge the novelty and interest of the study, however they raise the following major issues:

- unclear translational relevance
- inadequate choice of cell lines

Addressing these points and the other reviewers' concerns in full will be necessary for further considering the manuscript in our journal, and acceptance of the manuscript will entail a second round of review. EMBO Molecular Medicine encourages a single round of revision only and therefore, acceptance or rejection of the manuscript will depend on the completeness of your responses included in the next, final version of the manuscript. For this reason, and to save you from any frustrations in the end, I would strongly advise against returning an incomplete revision.

Please note in particular that demonstration of translational relevance will be required for further consideration in EMM.

If you would like to discuss further the points raised by the referees, I am available to do so via email or video. Let me know if you are interested in this option.

Revised manuscripts should be submitted within three months of a request for revision; they will otherwise be treated as new submissions, except under exceptional circumstances in which a short extension is obtained from the editor.

When submitting your revised manuscript, please carefully review the instructions that follow below. We perform an initial quality control of all revised manuscripts before re-review; failure to include requested items will delay the evaluation of your revision.

We require:

1) A .docx formatted version of the manuscript text (including legends for main figures, EV figures and tables). Please make sure that the changes are highlighted to be clearly visible.

2) Individual production quality figure files as .eps, .tif, .jpg (one file per figure). For guidance, download the 'Figure Guide PDF' (https://www.embopress.org/page/journal/17574684/authorguide#figureformat).

3) At EMBO Press we ask authors to provide source data for the main figures. Our source data coordinator will contact you to discuss which figure panels we would need source data for and will also provide you with helpful tips on how to upload and organize the files.

4) A .docx formatted letter INCLUDING the reviewers' reports and your detailed point-by-point responses to their comments. As part of the EMBO Press transparent editorial process, the point-by-point response is part of the Review Process File (RPF), which will be published alongside your paper.

5) A complete author checklist, which you can download from our author guidelines (https://www.embopress.org/page/journal/17574684/authorguide#submissionofrevisions). Please insert information in the checklist that is also reflected in the manuscript. The completed author checklist will also be part of the RPF.

6) Please note that all corresponding authors are required to supply an ORCID ID for their name upon submission of a revised manuscript.

7) It is mandatory to include a 'Data Availability' section after the Materials and Methods. Before submitting your revision, primary datasets produced in this study need to be deposited in an appropriate public database, and the accession numbers and database listed under 'Data Availability'. Please remember to provide a reviewer password if the datasets are not yet public (see https://www.embopress.org/page/journal/17574684/authorguide#dataavailability).
In case you have no data that requires deposition in a public database, please state so in this section. Note that the Data

Availability Section is restricted to new primary data that are part of this study.

8) For data quantification: please specify the name of the statistical test used to generate error bars and P values, the number (n) of independent experiments (specify technical or biological replicates) underlying each data point and the test used to calculate p-values in each figure legend. The figure legends should contain a basic description of n, P and the test applied. Graphs must include a description of the bars and the error bars (s.d., s.e.m.). Please provide exact p values.

9) Our journal encourages inclusion of *data citations in the reference list* to directly cite datasets that were re-used and obtained from public databases. Data citations in the article text are distinct from normal bibliographical citations and should directly link to the database records from which the data can be accessed. In the main text, data citations are formatted as follows: "Data ref: Smith et al, 2001" or "Data ref: NCBI Sequence Read Archive PRJNA342805, 2017". In the Reference list, data citations must be labeled with "[DATASET]". A data reference must provide the database name, accession number/identifiers and a resolvable link to the landing page from which the data can be accessed at the end of the reference. Further instructions are available at .

10) We replaced Supplementary Information with Expanded View (EV) Figures and Tables that are collapsible/expandable online. A maximum of 5 EV Figures can be typeset. EV Figures should be cited as 'Figure EV1, Figure EV2" etc... in the text and their respective legends should be included in the main text after the legends of regular figures.
- For the figures that you do NOT wish to display as Expanded View figures, they should be bundled together with their legends in a single PDF file called *Appendix*, which should start with a short Table of Content. Appendix figures should be referred to in the main text as: "Appendix Figure S1, Appendix Figure S2" etc.
- Additional Tables/Datasets should be labeled and referred to as Table EV1, Dataset EV1, etc. Legends have to be provided in a separate tab in case of .xls files. Alternatively, the legend can be supplied as a separate text file (README) and zipped together with the Table/Dataset file.
See detailed instructions here:

11) The paper explained: EMBO Molecular Medicine articles are accompanied by a summary of the articles to emphasize the major findings in the paper and their medical implications for the non-specialist reader. Please provide a draft summary of your article highlighting
- the medical issue you are addressing,
- the results obtained and
- their clinical impact.
This may be edited to ensure that readers understand the significance and context of the research. Please refer to any of our published articles for an example.

12) For more information: There is space at the end of each article to list relevant web links for further consultation by our readers. Could you identify some relevant ones and provide such information as well? Some examples are patient associations, relevant databases, OMIM/proteins/genes links, author's websites, etc...

13) Author contributions: CRediT has replaced the traditional author contributions section because it offers a systematic machine readable author contributions format that allows for more effective research assessment. Please remove the Authors Contributions from the manuscript and use the free text boxes beneath each contributing author's name in our system to add specific details on the author's contribution. More information is available in our guide to authors.

14) Disclosure statement and competing interests: We updated our journal's competing interests policy in January 2022 and request authors to consider both actual and perceived competing interests. Please review the policy https://www.embopress.org/competing-interests and update your competing interests if necessary.

15) Every published paper now includes a 'Synopsis' to further enhance discoverability. Synopses are displayed on the journal webpage and are freely accessible to all readers. They include a short stand first (maximum of 300 characters, including space) as well as 2-5 one-sentences bullet points that summarizes the paper. Please write the bullet points to summarize the key NEW findings. They should be designed to be complementary to the abstract - i.e. not repeat the same text. We encourage inclusion of key acronyms and quantitative information (maximum of 30 words / bullet point). Please use the passive voice. Please attach these in a separate file or send them by email, we will incorporate them accordingly.

Please also suggest a striking image or visual abstract to illustrate your article as a PNG file 550 px wide x 300-600 px high.

16) As part of the EMBO Publications transparent editorial process initiative (see our Editorial at http://embomolmed.embopress.org/content/2/9/329), EMBO Molecular Medicine will publish online a Review Process File (RPF) to accompany accepted manuscripts.
In the event of acceptance, this file will be published in conjunction with your paper and will include the anonymous referee reports, your point-by-point response and all pertinent correspondence relating to the manuscript. Let us know whether you agree with the publication of the RPF and as here, if you want to remove or not any figures from it prior to publication.

Please note that the Authors checklist will be published at the end of the RPF.

EMBO Molecular Medicine has a "scooping protection" policy, whereby similar findings that are published by others during review or revision are not a criterion for rejection. Should you decide to submit a revised version, I do ask that you get in touch after three months if you have not completed it, to update us on the status.

I look forward to receiving your revised manuscript.

Yours sincerely,

Lise Roth

Lise Roth, PhD
Senior Editor
EMBO Molecular Medicine

***** Reviewer's comments *****

Referee #1 (Comments on Novelty/Model System for Author):

This manuscript is a beautiful story with very well controlled experiments. However, unless the authors demonstrate that LKB1 activity is indeed low in uveal melanoma primary tumours, the model might be irrelevant for the disease.

Referee #1 (Remarks for Author):

The manuscript by Hireche-Proteau et al. reports that 'LKB1-SIK2 loss drives uveal melanoma proliferation and hypersensitivity to SLC8A1 and ROS inhibition'. The authors identified LKB1 as a potential synthetic viable gene in the uveal melanoma cell line OMM1.3 derived from a liver metastasis using a kinome-wide CRISPR/Cas9 knockout screen. The authors confirmed the synthetic viability of the loss of LKB1 in two additional uveal melanoma cell lines (one primary and one liver metastasis). Then, the authors showed very elegantly in a series of experiments the necessity and sufficiency of the LKB1-SIK2-SLC8A1 cascade in cell proliferation in vitro and tumour growth in vivo in mouse xenografts. In addition, the authors observed that the combination of a SLC8A1 inhibitor with a mitochondria-targeted antioxidant increased apoptosis in vitro and decreased tumour growth in mouse xenografts of LKB1-deficient cells in a synergistic manner. Finally, a gene signature for reduced LKB1 activity and prognosis in uveal melanoma tumours was proposed. Overall, the manuscript is well written and experiments were carefully controlled. Conclusions are mostly supported by the data presented. However, there are several concerns that need to be addressed before the manuscript can be accepted for publication, as follows:

- It is well known in the literature that LKB1 is a negative regulator of the mammalian target of rapamycin complex 1 (mTORC1). Thus, it is expected that the knockout of LKB1 would increase the activity of mTORC1 and result in higher cell proliferation and tumour growth. Therefore, similar results might be observed in these uveal melanoma cell lines by knocking out other negative regulators of mTORC1, such as TSC2 and PTEN. Since LKB1 mutations are very rare in uveal melanoma, the authors should demonstrate with solid evidence that LKB1 activity is actually diminished in uveal melanoma tumours, either by qPCR, RNA-seq, western blotting or reverse-phase protein arrays. Otherwise, although this study was very nicely controlled, its functional significance would be very limited and the LKB1-SIK2-SLC8A1 cascade might not be relevant for uveal melanoma pathogenesis. Indeed, the combination of a SLC8A1 inhibitor with a mitochondria-targeted antioxidant was only effective to decrease tumour growth in mouse xenografts of LKB1-deficient cells, but not in LKB1-competent cells. This raises serious concerns about the potential use of these drugs in uveal melanoma patients, unless they have LKB1 mutations, which are very rare in this tumour type.

- The rationale for selecting the 11 genes of the gene signature associated with LKB1 depletion and not others from the RNA-seq analysis should be provided. The scoring system should also be explained. For instance, the number of genes that need to be upregulated or downregulated in UM-TCGA in the same way as these genes in the LKB1-deficient RNA-seq for having a high or low signature score. In addition, further evidence that LKB1 is indeed inhibited in these UM-TCGA tumours is critical. Anyway, this gene signature requires to be validated in an independent cohort of uveal melanoma tumours to have significance in prognosis.

- The ROC analysis conducted might not be appropriate. A time-dependent ROC curve estimation from censored survival data,

such as the R package SurvivalROC, should be more suitable for predicting prognosis of the gene signatures.

- The 'Material and methods' section should be more detailed, such as providing identifiers for all Addgene plasmids used in this study and information on the SIK2 plasmids, as well as cell numbers used for the colony formation assays.

- The authors should provide the full list of genes and their Mageck scores of the kinome-wide CRISPR/Cas9 knockout screen as a Supplementary Excel file. The RNA-Seq data summarised in Fig. 3A should be deposited in a public repository, such as Gene Expression Omnibus (GEO) or ArrayExpress. The list of primers should be displayed as a Supplementary table.

- The treatment with KB-R7943 and/or MitoQ of mice with xenografts of control cells was indicated in the Results section, but the figures are missing. Only the figures of the LKB1-deficient xenografts were displayed. Please display these figures in the Supplementary section.

- The rationale for using 5 mg/kg of KB-R7943 and MitoQ and not other concentrations, as well as injecting intraperitoneally 3 times per week and not daily should be provided.

Referee #3 (Comments on Novelty/Model System for Author):

The manuscript is novel within the uveal melanoma field. Despite over 5 decades of active research, UM is still an unmet medical need. LKB1 expression is mutated in several cancers. Epigenetic regulation of this transcription factor is associated with several tumors. The loss of LKB1 makes the tumor cell more sensitive to oxidative stress and consequently to stress-inducing treatments, such as chemotherapy and radiotherapy. Therefore, it is an important molecule to characterize in UM.

The authors do an in depth study and demonstrate that SIK2 inhibition and upregulation of SLC8A1 is the mechanism of action to which LKB1 works in UM cells.

Technical quality of the manuscript is good. However, there are some technicalities that could have improved the manuscript and elevate its quality and impact. One is that the main cell line used is OMM1.3, also known as OMM2.3. To confirm some of the studies the authors tested OMM2.5, another metastatic cell line. Authentication studies have demonstrated that these two come from the same patient (B. Kasander's Lab). By doing this the authors pose additional questions, as it will be more interesting to test other metastatic cell lines to demonstrate the LKB1-SIK2 signaling node is critical in metastatses. Also, Mel270 is the primary tumor cell line from which OMM1.3/OMM2.5 was derived. How does this signaling expression is in these cells? These cells micrometastasize before the full blown liver metastasis. Can we determine this signaling node is different in those cells? Do primary and metastatic behaves differently? 92.1 cells were used in some studies, however, this bring additional set of questions, as they have high pigmentation which poses a challenge in RNA and DNA transcriptomic studies, and some studies suggest that although primary, 92.1 behaves as metastatic cells.

Referee #3 (Remarks for Author):

The manuscript is novel and has great implications in potential targeted treatments and biomarkers. Technical quality of the manuscript is good. However, there are some technicalities that could have improved the manuscript and elevate its quality and impact.

One is that the main cell line used is OMM1.3, also known as OMM2.3. To confirm some of the studies the authors tested OMM2.5, another metastatic cell line. Authentication studies have demonstrated that these two come from the same patient (B. Kasander's Lab). By doing this the authors pose additional questions, as it will be more interesting to test other metastatic cell lines to demonstrate the LKB1-SIK2 signaling node is critical in metastatses. Also, Mel270 is the primary tumor cell line from which OMM1.3/OMM2.5 was derived. How does this signaling expression is in these cells? These cells micrometastasize before the full blown liver metastasis. Can we determine this signaling node is different in those cells? Do primary and metastatic behaves differently? 92.1 cells were used in some studies, however, this bring additional set of questions, as they have high pigmentation which poses a challenge in RNA and DNA transcriptomic studies, and some studies suggest that although primary, 92.1 behaves as metastatic cells.

Additional comments:
Could the authors publish the transcriptomic data?
The authors suggest the results of SLC8A1 in liver mets is due to favors liver tropism. Did you see this in all cultures across the UM cell lines tested? How was it in primary ones?
Are there differences in mtROS in UM primary versus metastatic?

Reagents/methodology
Were these cells authenticated?
Please include cat numbers in the biochemicals, and the Addgene plasmids.

Did you test additional LKB1 Abs? The one used is anti mouse but it cross reacts with human. Have you tested monoclonal anti-human Abs?

Referee #1 (Comments on Novelty/Model System for Author):

This manuscript is a beautiful story with very well controlled experiments. However, unless the authors demonstrate that LKB1 activity is indeed low in uveal melanoma primary tumours, the model might be irrelevant for the disease.

Thank you for your positive evaluation of the manuscript.

Referee #1 (Remarks for Author):

The manuscript by Hireche-Proteau et al. reports that 'LKB1-SIK2 loss drives uveal melanoma proliferation and hypersensitivity to SLC8A1 and ROS inhibition'. The authors identified LKB1 as a potential synthetic viable gene in the uveal melanoma cell line OMM1.3 derived from a liver metastasis using a kinome-wide CRISPR/Cas9 knockout screen. The authors confirmed the synthetic viability of the loss of LKB1 in two additional uveal melanoma cell lines (one primary and one liver metastasis). Then, the authors showed very elegantly in a series of experiments the necessity and sufficiency of the LKB1-SIK2-SLC8A1 cascade in cell proliferation in vitro and tumour growth in vivo in mouse xenografts. In addition, the authors observed that the combination of a SLC8A1 inhibitor with a mitochondria-targeted antioxidant increased apoptosis in vitro and decreased tumour growth in mouse xenografts of LKB1-deficient cells in a synergistic manner. Finally, a gene signature for reduced LKB1 activity and prognosis in uveal melanoma tumours was proposed. Overall, the manuscript is well written and experiments were carefully controlled. Conclusions are mostly supported by the data presented. However, there are several concerns that need to be addressed before the manuscript can be accepted for publication, as follows:

- It is well known in the literature that LKB1 is a negative regulator of the mammalian target of rapamycin complex 1 (mTORC1). Thus, it is expected that the knockout of LKB1 would increase the activity of mTORC1 and result in higher cell proliferation and tumour growth. Therefore, similar results might be observed in these uveal melanoma cell lines by knocking out other negative regulators of mTORC1, such as TSC2 and PTEN.

We agree with the reviewer that LKB1 is reported in many cases as a negative regulator of the mammalian target of rapamycin complex 1 (mTORC1). However, there are also several examples in the literature showing that LKB1 can function independently of this pathway. As indicated by the reviewer, both AMPK that is regulated by LKB1 and PTEN, regulate the mTORC1/mTOR signaling pathway.
We performed western blot experiments (see below) showing that LKB1 depletion does not affect AMPK or mTOR phosphorylation, indicating that AMPK or mTOR activity is not mainly regulated by LKB1 in uveal melanoma cells. Reinforcing the idea of AMPK independent module, we and other showed that AMPK knockdown does not stimulate uveal melanoma cell growth (see discussion part of the manuscript, page 18).

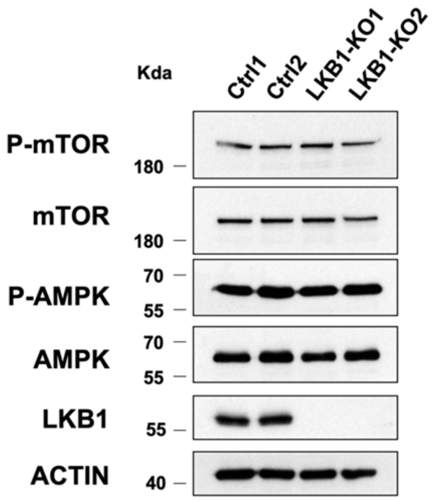

Since LKB1 mutations are very rare in uveal melanoma, the authors should demonstrate with solid evidence that LKB1 activity is actually diminished in uveal melanoma tumours, either by qPCR, RNA-seq, western blotting or reverse-phase protein arrays. Otherwise, although this study was very nicely controlled, its functional significance would be very limited and the LKB1-SIK2-SLC8A1 cascade might not be relevant for uveal melanoma pathogenesis. Indeed, the combination of a SLC8A1 inhibitor with a mitochondria-targeted antioxidant was only effective to decrease tumour growth in mouse xenografts of LKB1-deficient cells, but not in LKB1-competent cells. This raises serious concerns about the potential use of these drugs in uveal melanoma patients, unless they have LKB1 mutations, which are very rare in this tumour type.

We agree with the reviewer that LKB1 mutations are very rare in uveal melanoma as we mentioned in the result (page 9 in the revised version) and discussion sections (first paragraph, page 18 in the revised version) of the submitted version of our manuscript. As discussed in our manuscript, nongenetic inactivation or reduced expression could be also responsible for a decreased LKB1 activity.
We already showed in the submitted version of our manuscript using IHC that LKB1 expression can be very low/absent in metastatic uveal melanomas (Fig. 2B). No LKB1 expression will necessarily translate into inhibition of its activity. This is the first demonstration that LKB1 activity can be lost in metastatic uveal melanoma.

In the revised manuscript, we screened for the effect of hepatocyte growth factor (HGF), which can be produced in the eye, the skin and the liver, on LKB1 activity. Our data show that HGF increases the phosphorylation of LKB1 on serine 428 and reduces the phosphorylation of SIK2 on threonine 175. LKB1-ser428 phosphorylation has been associated with inhibition of LKB1 activity in cancers, including cutaneous melanoma (PMID: 35992082 and PMID: 19187764). In line with SIK2 functioning downstream LKB1, we observed reduced SIK2-thr175 phosphorylation, which is a reflet of LKB1 inactivation. In agreement with LKB1-KO effect, we found that HGF also stimulates colony formation capacity of metastatic

uveal melanoma cells. These results have been added in the revised version (Fig. 2C-D) and discussed in the discussion section (page 18 in the revised manuscript). Altogether, our data implicated inhibition of both LKB1 expression and activity in uveal melanomas.

Regards to the second point, we disagree with the reviewer. *In vivo*, LKB1-WT cells did not grow within the time frame of the experiments, thus, we didn't assess the effect of the drug combination on LKB1 wild-type cells. However, *in vitro* the drug combination (SLC8A1 inhibitor with a mitochondria-targeted antioxidant) triggers cell death in both LKB1-WT (about 40%) and LKB1-KO cells (about 80%), although LKB1-KO cells are more sensitive than the LKB1-WT cells (Fig. 6C). As per results described above, our results support the rationale of the potential use of SLC8A1 inhibitor in combination with a mitochondria-targeted antioxidant to treat uveal melanomas.

- The rationale for selecting the 11 genes of the gene signature associated with LKB1 depletion and not others from the RNA-seq analysis should be provided. The scoring system should also be explained. For instance, the number of genes that need to be upregulated or downregulated in UM-TCGA in the same way as these genes in the LKB1-deficient RNA-seq for having a high or low signature score. In addition, further evidence that LKB1 is indeed inhibited in these UM-TCGA tumours is critical. Anyway, this gene signature requires to be validated in an independent cohort of uveal melanoma tumours to have significance in prognosis.

To generate the gene signature associated with LKB1 depletion we first selected differentially expressed genes as follow, log2(foldchange) ≥ 1 and adjusted p-value ≤ 0.05. We sorted by adjusted p-value and kept the TOP 100 protein coding genes. Then, genes were filtered out based on their expression level within the TCGA-UM cohort. We further explored the retained genes by using Cox regression model to calculate the hazard ratio (HR) and kept the TOP genes based on their hazard ratio. This algorithm allowed us to identify an 11 gene signature, composed of genes that are upregulated when LKB1 is depleted. Therefore, the LKB1 loss score is obtained by addition of the expression of each individual gene. The more they are expressed, higher is the score and vice versa. (A paragraph has been added in the Material and methods section, RNA-sequencing and gene signature, page 23 in the revised version)

As requested by the reviewer, we have validated our signature in an independent cohort of uveal melanoma tumours (63 patients, GSE22138) to improve significance in prognosis (Fig. EV5C in the revised version). We used a time-dependent ROC curve estimation from censored survival data available with the R package SurvivalROC as requested by the reviewer in the next point.

- The ROC analysis conducted might not be appropriate. A time-dependent ROC curve estimation from censored survival data, such as the R package SurvivalROC, should be more suitable for predicting prognosis of the gene signatures.

As requested by the reviewer, we have performed a time-dependent ROC curve estimation from censored survival data using the R package SurvivalROC which is new Fig. 7D in the revised version.

- The 'Material and methods' section should be more detailed, such as providing identifiers for all Addgene plasmids used in this study and information on the SIK2 plasmids, as well as cell numbers used for the colony formation assays.

We agree with the reviewer. Identifiers for all Addgene plasmids used in our study, information on the SIK2 plasmids, as well as cell numbers used for the colony formation assays are provided in the material and methods section of the revised manuscript.

- The authors should provide the full list of genes and their Mageck scores of the kinome-wide CRISPR/Cas9 knockout screen as a Supplementary Excel file. The RNA-Seq data summarised in Fig. 3A should be deposited in a public repository, such as Gene Expression Omnibus (GEO) or ArrayExpress.
The list of primers should be displayed as a Supplementary table.

We agree with the reviewer.
The full list of genes and their Mageck scores are provided as a new Table 1 in the revised version.
RNA-Seq data have been deposited in the NCBI Gene Expression Omnibus (GEO) database (https://www.ncbi.nlm.nih.gov/geo/) under the SuperSerie GSE233458. In the revised manuscript, GSE number is indicated in the material and methods section, paragraph “Data availability“.

We agree with the reviewer. Since we used primers for only 2 genes, we provided their sequences directly in the material and methods section, paragraph “mRNA preparation and real-time/quantitative PCR » in the revised version.

- The treatment with KB-R7943 and/or MitoQ of mice with xenografts of control cells was indicated in the Results section, but the figures are missing. Only the figures of the LKB1-deficient xenografts were displayed. Please display these figures in the Supplementary section.

Thanks to the reviewer for spotting this out. As shown in figure 1E, OMM1.3 control cells hardly grow as xenograft, tumors start to be visible after 40 days. In figure 7A, xenografts formed with OMM1.3 control cells were not detectable in the time frame of the experiment. In the revised version, the paragraph has been modified by: “When the tumors, formed with LKB1-KO cells, reached 100 mm^3, they were treated with KB-R7943, MitoQ or their combination and vehicle. Of note, control cells did not grow as xenografts within the time frame of the experiment” (page 16 in the revised version).

- The rationale for using 5 mg/kg of KB-R7943 and MitoQ and not other concentrations, as well as injecting intraperitoneally 3 times per week and not daily should be provided.

Thanks for the question. We screened the literature and found different treatment regimens based on intraperitoneal injection of KB-R7943, such as daily (PMID: 27275542) or twice a week (PMID: 19661061) at 10 mg/kg. We also found different treatment regimens based on intraperitoneal injection of MitoQ such as 3

consecutive days (doi.org/10.3390/antiox12020453), or twice per week for 3 weeks (doi.org/10.1016/j.bbadis.2013.09.005) at 5 mg/kg or 10 mg/kg. We chose injection 3 times per week that allowed to cover the different protocol published for both drugs. Moreover, we did preliminary experiments using KB-R7943 5 mg/kg or 10 mg/kg. Intraperitoneal injection of 10 mg/kg KB-R7943 3 times per week had a small effect on mouse body weight. Given that our experiment, based on the in vitro data, aimed to study the combination of KB-R7943 and MitoQ, we chose to reduce KB-R7943 dosage to 5 mg/kg and to use similar dosage for MitoQ. This combination triggered inhibition of tumor growth without toxicity in the mice (Fig. 7A-B and Supplementary fig. 8A of the initial manuscript or Fig. 7A-B and Fig. EV5A of the revised version).

Referee #3 (Comments on Novelty/Model System for Author):

The manuscript is novel within the uveal melanoma field. Despite over 5 decades of active research, UM is still an unmet medical need. LKB1 expression is mutated in several cancers. Epigenetic regulation of this transcription factor is associated with several tumors. The loss of LKB1 makes the tumor cell more sensitive to oxidative stress and consequently to stress-inducing treatments, such as chemotherapy and radiotherapy.
Therefore, it is an important molecule to characterize in UM.

The authors do an in depth study and demonstrate that SIK2 inhibition and upregulation of SLC8A1 is the mechanism of action to which LKB1 works in UM cells.

Technical quality of the manuscript is good. However, there are some technicalities that could have improved the manuscript and elevate its quality and impact. One is that the main cell line used is OMM1.3, also known as OMM2.3. To confirm some of the studies the authors tested OMM2.5, another metastatic cell line. Authentication studies have demonstrated that these two come from the same patient (B. Kasander's Lab). By doing this the authors pose additional questions, as it will be more interesting to test other metastatic cell lines to demonstrate the LKB1-SIK2 signaling node is critical in metastatses. Also, Mel270 is the primary tumor cell line from which OMM1.3/OMM2.5 was derived. How does this signaling expression is in these cells? These cells micrometastasize before the full blown liver metastasis. Can we determine this signaling node is different in those cells? Do primary and metastatic behaves differently? 92.1 cells were used in some studies, however, this bring additional set of questions, as they have high pigmentation which poses a challenge in RNA and DNA transcriptomic studies, and some studies suggest that although primary, 92.1 behaves as metastatic cells.

Given that the different points raised in this section are taken up by the reviewer in the next section, we answer all these points after.

Referee #3 (Remarks for Author):

The manuscript is novel and has great implications in potential targeted treatments and biomarkers. Technical quality of the manuscript is good. However, there are some technicalities that could have improved the manuscript and elevate its quality and impact.

Thank you for your positive evaluation of the manuscript and for your suggestions for improvement.

One is that the main cell line used is OMM1.3, also known as OMM2.3. To confirm some of the studies the authors tested OMM2.5, another metastatic cell line. Authentication studies have demonstrated that these two come from the same patient (B. Kasander's Lab).
By doing this the authors pose additional questions, as it will be more interesting to test other metastatic cell lines to demonstrate the LKB1-SIK2 signaling node is critical in metastatses.

We agree with the reviewer that OMM1.3 cell line, also known as OMM2.3, and OMM2.5 cell line comes from the same patients. That said, these two tumors behave very differently.
As requested by the reviewer, we performed the deletion of LKB1 using the CRISPR/Cas9 approach in OMM1 cells which is a different human metastatic uveal melanoma cell line. In agreement with what we observed in OMM1.3 and OMM2.5, LKB1 knock out in OMM1 cells increased their colony formation capacity. This result is shown in the revised version in the new Fig. EV1D.

Also, Mel270 is the primary tumor cell line from which OMM1.3/OMM2.5 was derived. How does this signaling expression is in these cells? These cells micrometastasize before the full blown liver metastasis. Can we determine this signaling node is different in those cells? Do primary and metastatic behaves differently?

As suggested by the reviewer, we performed the deletion of LKB1 using the CRISPR/Cas9 approach in Mel270 cells. As found in the other cell lines, LKB1 knockout in Mel270 cells enhances their colony formation capacity. This result is shown in the revised manuscript in the new Fig. EV1F. In total, in the revised manuscript, we carried out LKB1 knockout in 5 different cell lines (Fig. EV1B-F), 3 metastatic (OMM1, OMM1.3, OMM2.5) and 2 primary (92.1, Mel270) and recurrently observed an increase in their colony formation capacity.
Moreover, in the new Fig. EV4C-D of the revised version, our data reveal that forced expression of SIK2 in OMM2.5, OMM1 and 92.1, Mel270 (Appendix 2) cells rescued the colony forming capacity phenotype mediated by LKB1 loss.
Therefore, these data indicate that primary and metastatic uveal melanoma cells similarly showed increased colony formation capacity upon LKB1 loss and that the LKB1-SIK2 module is functional in both primary and metastatic cells.

92.1 cells were used in some studies, however, this bring additional set of questions, as they have high pigmentation which poses a challenge in RNA and DNA transcriptomic studies, and some studies suggest that although primary, 92.1 behaves as metastatic cells.

We agree with the reviewer that pigmentation can affect the quality of RNA or DNA materials. However, in this study we didn’t prepare RNA or DNA materials from 92.1 for genomics studies nor RNA for qRT-PCR. Nevertheless, our team has a huge

expertise in the field of pigmented cells and we know how to cope with melanin in DNA/RNA preparation.
Moreover, in addition to 92.1, we performed experiments in Mel270, another primary cell line, and in different metastatic cells as described above.

Additional comments:
Could the authors publish the transcriptomic data?

Thanks for asking. RNA-Seq data have been deposited in the NCBI Gene Expression Omnibus (GEO) database (https://www.ncbi.nlm.nih.gov/geo/) under the SuperSerie GSE233458. This information has been added in the revised manuscript in the paragraph “Data availabilities” of the material and methods section.

The authors suggest the results of SLC8A1 in liver mets is due to favors liver tropism. Did you see this in all cultures across the UM cell lines tested? How was it in primary ones?

The information provided to reviewers was removed

Are there differences in mtROS in UM primary versus metastatic?

The information and the figure provided for reviewers was removed

Reagents/methodology
Were these cells authenticated?

Cells were authenticated through short tandem repeat (STR) profiling. This is indicated in the revised manuscript in the paragraph "Cell cultures" of the material and methods section.

Please include cat numbers in the biochemicals, and the Addgene plasmids.

Thanks for the suggestion. Cat numbers in the biochemicals, and the Addgene plasmids have been updated in the material and methods section of the revised manuscript.

Did you test additional LKB1 Abs? The one used is anti mouse but it cross reacts with human. Have you tested monoclonal anti-human Abs?

In this study, we used 2 different LKB1 Antibody.
The LKB1 (D60C5) Antibody from Cell Signaling Technology was used for western blot experiments and the antigenic peptide comes from the human species (UniProt ID: Q15831). It is a monoclonal antibody developed in rabbit that recognizes LKB1 of human origin.
The LKB1 antibody used for immunohistochemistry (sc-374334, Santa Cruz Biotechnology) is a monoclonal antibody developed in mouse that recognizes LKB1 of human origin.

10th Aug 2023

Dear Dr. Strub,

Thank you for the submission of your revised manuscript to EMBO Molecular Medicine. We have now received the enclosed reports from the two initial referees. As you will see below, referee #3 is satisfied with the revisions. However, referee #1, while acknowledging the quality of the work that was done to answer the initial issues, also points out that the translational relevance remains unclear as no convincing evidence of decreased LKB1 activity in uveal melanoma was presented.
Upon further cross-commenting, this referee added:
"Important experiments to show evidence of decreased LKB1 activity in uveal melanoma could be analysing the immunohistochemistry, RNA-Seq, qPCR, western blotting or reverse-phase protein arrays of primary uveal melanoma tumours compared to melanocytes or between high/low risk uveal melanoma or among other types of cancer. If the authors do not have access to these tumour samples in a timely manner, at least they could analyse The Cancer Genome Atlas (TCGA) and/or other public databases. Otherwise, their model might not be valid for uveal melanoma."

Given this essential concern (that was raised in the initial review), and because at EMBO Press we encourage a single round of major revisions, I am afraid that we cannot offer to consider the manuscript further.

However, should you be able to convincingly show LKB1 decreased activity in primary uveal melanoma and thus demonstrate the clinical relevance of your findings, we would be happy to reconsider our decision.

I am very sorry that I could not bring better news this time and understand your disappointment. Still, I hope that the referees' comments will be helpful in your continued work in this area.

Yours sincerely,

Lise Roth

Lise Roth, PhD
Senior Editor
EMBO Molecular Medicine

***** Reviewer's comments *****

Referee #1 (Comments on Novelty/Model System for Author):

This manuscript shows a beautiful story with very well controlled experiments. However, the model system might not be relevant for uveal melanoma tumorigenesis unless the authors demonstrate that LKB1 activity is decreased in uveal melanoma primary tumours.

Referee #1 (Remarks for Author):

The authors provided a revised version of their manuscript and fulfilled some of the comments suggested. However, the most important concern of this study is the biological/functional significance of LKB1 in uveal melanoma, which still remains uncertain. Solid evidence that LKB1 activity is decreased in uveal melanoma tumours was requested. Since LKB1 mutations in uveal melanoma are very rare, the authors claim that "nongenetic inactivation or reduced expression could be also responsible for a decreased LKB1 activity". The main evidences provided by the authors are:

- IHC of LKB1 (Fig. 2B). There are several concerns about this source of evidence. Firstly, there is heterogeneity in LKB1 expression in one of the tumours, with some areas of high expression and others of no expression, which might be necrotic. Secondly, only the IHC of three tumours were displayed and all of them showed LKB1 expression (partly in one and completely in two of them). Thus, large numbers of IHC staining would be necessary to have statistical power to be representative. Thirdly, the tumours were skin metastases of uveal melanoma, which might differ from the original tumour. Hence, the IHC should be done in a large cohort of primary uveal melanoma tumours to discern the incidence of low LKB1 expression.

- HGF increases LKB1 phosphorylation, decreases SIK2 phosphorylation and induces colony formation (Fig. 2C-D). These

experiments are interesting, but interventional. It can be concluded that LKB1 is inhibited in uveal melanoma cell lines upon exposure to HGF. However, whether LKB1 is inhibited or not in uveal melanoma patients remains elusive.

- Western blot of phospho-mTOR. The authors claim that "LKB1 depletion does not affect AMPK or mTOR phosphorylation, indicating that AMPK or mTOR activity is not mainly regulated by LKB1 in uveal melanoma cells". This western blot is not conclusive, because phospho-mTOR is a bad readout of the mTOR activity. More efficient and specific readouts of mTORC1 activity are the phosphorylation of ribosomal protein S6, ribosomal protein S6 kinase and eukaryotic translation initiation factor 4E binding protein 1 (EIF4EBP1). Thus, the western blot should be done to evaluate whether the phosphorylation of any of these downstream mTORC1 targets is increased upon LKB1 knockout, indicating an activation of the mTORC1 pathway. If that is the case, the authors should knockout/knockdown other negative regulators of mTORC1, such as TSC1, TSC2 and PTEN to test whether similar effects are observed and mediated by activation of mTORC1.

Overall, the pieces of evidence provided in this study are insufficient to support the idea that LKB1 is inhibited/decreased in uveal melanoma. To get further insight about this topic, this reviewer did a quick Pan-Cancer analysis of TCGA expression data for LKB1/STK11. It showed that uveal melanoma tumours are the second cancer type from TCGA with the highest LKB1/STK11 RNAseq gene expression of the 33 cancer types evaluated. This might explain the large effects observed by LKB1 knockout. However, it raises a serious concern about the hypothesis of this study about LKB1 inhibition in uveal melanoma, being the opposite, where the decrease of LKB1 expression in uveal melanoma patients seems to be marginal. The authors should therefore provide conclusive evidence about this hypothesis in primary uveal melanoma tumours. Otherwise, this nice study with very well controlled experiments would represent a model system that might be irrelevant for the tumorigenesis of uveal melanoma. Moreover, testing a SLC8A1 inhibitor with a mitochondria-targeted antioxidant in clinical trials for treating uveal melanoma patients might represent serious ethical concerns if the model hypothesis of this study is not true.

Regarding the gene signature associated with LKB1 depletion, it would be essential to show a correlation of this gene signature with a decrease of LKB1 expression. Otherwise, this gene signature might be misleading. According to the methodology used, the top-100 protein-coding genes differentially expressed by LKB1 knockout were further "filtered out based on their expression level within the TCGA-UM cohort". This step needs to be clarified about how many genes were filtered out and in what way. Anyway, the 11 genes of the gene signature seem to be selected ad hoc by their association with uveal melanoma patient survival. Then, it is not surprising that the expression of this gene signature is associated with patient survival. However, this gene signature might not be reflective of LKB1 depletion unless this is shown. It should be straightforward to compare the LKB1 expression levels of those TCGA patients with positive and negative gene signature to validate the association with LKB1 depletion.

Regarding the rationale for drug dosage in the mouse experiments, this information should be incorporated in the manuscript.

Referee #3 (Comments on Novelty/Model System for Author):

The identification of LKB1 as a synthetic viable gene in UM is very important. This could id in the prognosis of the UM tumors. I am impressed the authors took each of the reviewers points and addressed them experimentally through the CRISPR/Cas9 KO screen.

Referee #3 (Remarks for Author):

The authors addressed properly all the comments in a very thorough manner.

As a service to authors, EMBO provides authors with the possibility to transfer a manuscript that one journal cannot offer to publish to another EMBO publication. The full manuscript and if applicable, reviewers reports are automatically sent to the receiving journal to allow for fast handling and a prompt decision on your manuscript. For more details of this service, and to transfer your manuscript to another EMBO title please click on Link Not Available

Please do not share this URL as it will give anyone who clicks it access to your account.

Centre Méditerranéen de Médecine Moléculaire
U1065

Biologie et Pathologie des cellules mélanocytaires
(équipe 1)
C3M, Bâtiment Archimed, 151 Av Saint Antoine de Ginestière, 06204 Nice Cedex 2
Tel. 04 89 15 37 81. Email. Corine.Bertolotto@univ-cotedazur.fr

Nice, 09/03/2023

First of all, we would like to thank the referee who pushed us to the limit and enabled us to greatly improve our work, that now shows a correlation between our signature and the level of expression of LKB1, meeting now the main referee's concern.

Please find below a point-by-point response to referee #1's comments.

IHC of LKB1 (Fig. 2B). There are several concerns about this source of evidence. Firstly, there is heterogeneity in LKB1 expression in one of the tumours, with some areas of high expression and others of no expression, which might be necrotic. Secondly, only the IHC of three tumours were displayed and all of them showed LKB1 expression (partly in one and completely in two of them). Thus, large numbers of IHC staining would be necessary to have statistical power to be representative. Thirdly, the tumours were skin metastases of uveal melanoma, which might differ from the original tumour. Hence, the IHC should be done in a large cohort of primary uveal melanoma tumours to discern the incidence of low LKB1 expression.
We provide 8 more immunohistochemistry (IHC) images from 8 different patients **(Figure 1)**. Based on the expression and localization, once again we can observe that some tumors don't express LKB1, and others express LKB1 either in the cytoplasm or at the membrane. It is worth noting that (i) IHC has been conducted by specialists in the field of melanoma (Michell Levesque and collaborators) and (ii) that pathologists (2 co-authors on this publication) confirmed that there is no necrosis in these IHC. The new IHC stainings can be added to the supplementary data of our manuscript.
In conclusion these data confirm the heterogeneous expression and activity of LKB1 in metastatic uveal melanomas.

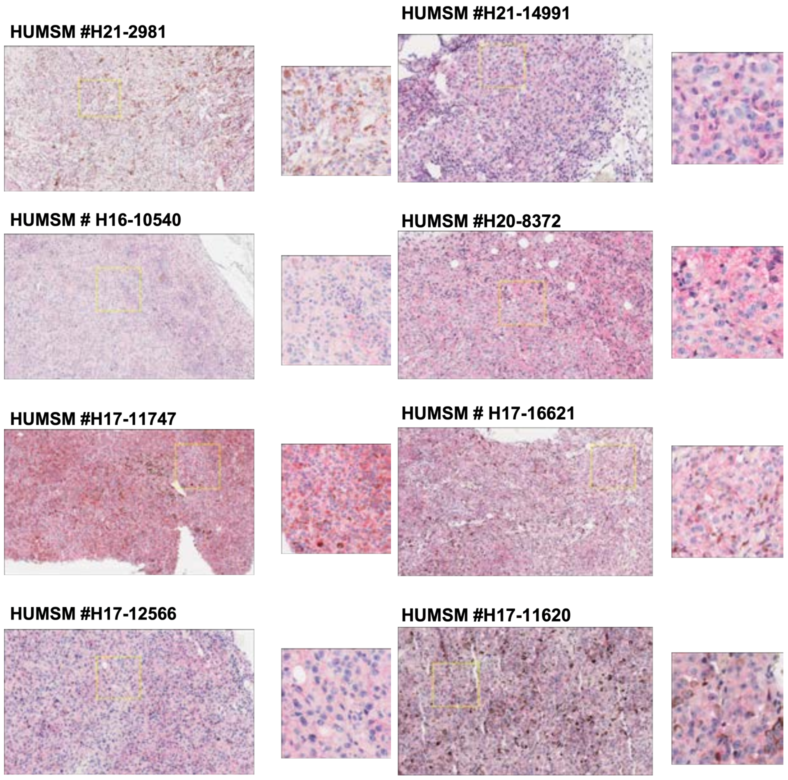

Figure 1: Immunohistochemical stainings for LKB1 were performed on skin metastasis of 8 different human uveal melanoma biopsies.

- HGF increases LKB1 phosphorylation, decreases SIK2 phosphorylation and induces colony formation (Fig. 2C-D). These experiments are interesting, but interventional. It can be concluded that LKB1 is inhibited in uveal melanoma cell lines upon exposure to HGF. However, whether LKB1 is inhibited or not in uveal melanoma patients remains elusive.

We agree with referee #1, these experiments are interventional, but as HGF is expressed in skin and in liver, where uveal melanomas form metastases, the inhibition of LKB1 by HGF has a clear clinical relevance.

- Western blot of phospho-mTOR. The authors claim that "LKB1 depletion does not affect AMPK or mTOR phosphorylation, indicating that AMPK or mTOR activity is not mainly regulated by LKB1 in uveal melanoma cells". This western blot is not conclusive, because phospho-mTOR is a bad readout of the mTOR activity. More efficient and specific readouts of mTORC1 activity are the phosphorylation of ribosomal protein S6, ribosomal protein S6 kinase and eukaryotic translation initiation factor 4E binding protein 1 (EIF4EBP1). Thus, the western blot should be done to evaluate whether the phosphorylation of any of these downstream mTORC1 targets is increased upon LKB1 knockout, indicating an activation of the mTORC1 pathway. If that is the case, the authors should Knockout/knockdown other negative regulators of mTORC1, such as TSC1,TSC2 and PTEN to test whether similar effects are observed and mediated by activation of mTORC1.

Phosphorylation at serine 2448 in mTOR shown in the revise manuscript might be a poor readout of the mTOR activity, but phosphorylation on this motif is done by p70S6K and is therefore a good readout of the p70S6K activity, as requested by referee #1. We do not really get the point of studying mTORC1 regulator as we show that the mTOR pathway is not activated after LKB1 loss in uveal melanoma.

Overall, the pieces of evidence provided in this study are insufficient to support the idea that LKB1 is inhibited/decreased in uveal melanoma. To get further insight about this topic, this reviewer did a quick Pan-Cancer analysis of TCGA expression data for LKB1/STK11. It showed that uveal melanoma tumours are the second cancer type from TCGA with the highest LKB1/STK11 RNAseq gene expression of the 33 cancer types evaluated. This might explain the large effects observed by LKB1 knockout. However, it raises a serious concern about the hypothesis of this study about LKB1 inhibition in uveal melanoma, being the opposite, where the decrease of LKB1 expression in uveal melanoma patients seems to be marginal. The authors should therefore provide conclusive evidence about this hypothesis in primary uveal melanoma tumours.
We agree with referee #1 that uveal melanomas are the second cancer type from TCGA with the highest STK11/LKB1 gene expression. However, again, this observation says nothing about LKB1 activity, which to our knowledge can only be evaluated by our signature. **Using our signature, we can show a greater variability of LKB1 activity among the primary uveal melanomas.**

Contrary to what referee #1 thinks, we are not going to initiate a clinical trial in the coming weeks, we have a long preclinical validation work to do first. **But, it is an avenue that must be explored given the lack of effective therapies for most metastatic uveal melanoma patients. To our knowledge, our signature is the first one with a predictive value of treatment regimen.**

Regarding the gene signature associated with LKB1 depletion, it would be essential to show a correlation of this gene signature with a decrease of LKB1 expression. Otherwise, this gene signature might be misleading.
We have compared LKB1 expression levels with our gene signature using the TCGA cohort. You can appreciate that low LKB1 expression is correlated with high level of our gene signature and that high LKB1 expression is correlated with low level of our gene signature with statistical significance **(Figure 2).**
These data address the major referee #1 concerns and must definitively convince him/her of the clinical interest of our data.

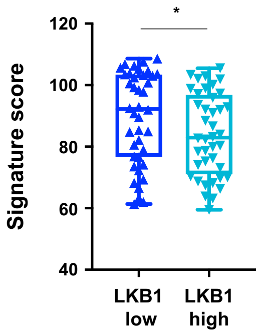

Figure 2: Representative box and whiskers plots of the LKB1 loss signature score based on LKB1 mRNA expression level (low and high) from the UM-TCGA dataset. Low LKB1 expression is correlated with high level of our gene signature score and high LKB1 expression is correlated with low level of our gene signature score. Mann-Whitney test was performed for comparison between groups. *p<0.05. All points are represented.

Nevertheless, we believe that our signature, in addition to identifying low/high LKB1 tumors, also provide an evaluation of the LKB1 activity. We can't use any other previous studies to compare with our data, because our study is first detailed study on LKB1 signaling in uveal melanoma. **Thus, our signature is so far the best way to evaluate LKB1 activity and has a much higher value than LKB1 expression alone.**

These data will be added and discussed in the revised manuscript.

According to the methodology used, the top-100 protein-coding genes differentially expressed by LKB1 knockout were further "filtered out based on their expression level within the TCGA-UM cohort". This step needs to be clarified about how many genes were filtered out and in what way. Anyway, the 11 genes of the gene signature seem to be selected ad hoc by their association with uveal melanoma patient survival. Then, it is not surprising that the expression of this gene signature is associated with patient survival. However, this gene signature might not be reflective of LKB1 depletion unless this is shown. It should be straightforward to compare the LKB1 expression levels of those TCGA patients with positive and negative gene signature to validate the association with LKB1 depletion.
We fully disagree with the reviewer. Of course, we tried to obtain the most relevant signature. What we did is to select 11 genes among the 27 genes remaining after the filtering operations. We can't be blamed for trying to find the best possible signature among the 27 genes resulting from our unsupervised analyses.
Nevertheless, a non-supervised analysis using the top 10 genes up-regulated after LKB1 loss from our transcriptomic data ranked by adjusted p-value also shows a high prognosis value **(Figure 3)** (ROC AUC=0.85, p<0.0001).

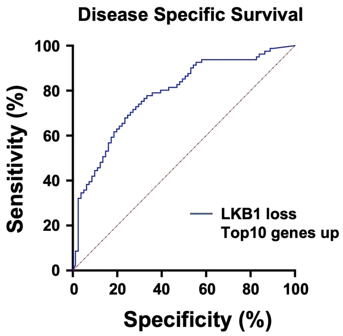

	LKB1 loss Top10 genes up
AUC	0.85
p-value	<0.0001

Figure 3: Time-dependent Receiver operating characteristic (ROC) curve show the sensitivity and specificity for the score of the Top10 upregulated genes upon LKB1 loss (RNA-seq data) ranked by adjusted p-value, for predicting the patient disease specific survival (UM-TCGA cohort).

Instituts thématiques Inserm
Institut national de la santé et de la recherche médicale
UNIVERSITÉ CÔTE D'AZUR

Gene name	Log2 FC (LKB1 KO vs control)	Adjusted p-value (LKB1 KO vs control)
ADCY1	5,844413545	1,26006E-77
KIAA1598	4,136844266	4,10776E-29
GRIN2A	3,810906698	2,20717E-18
SPOCK1	3,650336	3,05163E-18
SLC8A1	2,852637522	1,03953E-14
ECM1	2,455083114	3,80959E-14
IL4R	3,046195462	1,30172E-12
ATP8B4	3,12839952	1,43999E-11
RIPK4	2,963546731	5,425E-11
FAM107B	2,168552137	3,84692E-10

Table 1: List of the top 10 genes up-regulated after LKB1 loss from our transcriptomic data, ranked by adjusted p-value. Note that non-protein-coding genes and genes not expressed (https://xena.ucsc.edu/, UM-TCGA dataset) were removed from the list.

Regarding the rationale for drug dosage in the mouse experiments, this information should be incorporated in the manuscript.
We will introduce this information in the materials and methods section of the revised manuscript.

Instituts thématiques Inserm
Institut national de la santé et de la recherche médicale
UNIVERSITÉ CÔTE D'AZUR

Dear Dr. Bertolotto, dear Dr. Strub,

Thank you again for sending a rebuttal letter regarding referee #1's concerns.
I have now received the feedback from this referee, who makes the following comments:

- "It is great that the authors increased the number of immunohistochemistries up to 8 and excluded necrosis. A more detailed explanation of the differences in LKB1 protein expression would be recommended.
- The authors claim that LKB1 activity can only be evaluated by their signature. However, correlation not always implies causation. LKB1 activity should be able to be measured by its direct downstream targets, such as AMPKα and AMPKα-like proteins (NUAK kinases, microtubule affinity regulating kinases [MARKs] and salt inducible kinases [SIK]).
- The rationale for selecting the 11 genes of the signature out of 27 genes still remains unclear.
- It is claimed in this study that mTOR pathway is not activated after LKB1 loss in uveal melanoma. This is based on an unreliable marker of mTORC1 activity (mTOR phosphorylation). However, there is an overwhelming amount of evidence in the literature implying that mTORC1 signalling is hyperactivated after loss of LKB1 through multiple characterized mechanisms and is considered the major mechanism of how LKB1 loss contributes to oncogenesis (PMID: 29403037). At a minimum, the authors should show western blots with reliable markers of mTORC1 activity, such as pT389-S6K1 or pS6 (Ser235/236). Thereafter, it could be concluded whether LKB1 loss activates mTORC1 (or not) in uveal melanoma."

I hope you will view these suggestions addressable, and I look forward to receiving your revised manuscript.

Please let me know if you have further concerns.

With kind regards,

Lise Roth

Centre Méditerranéen de Médecine Moléculaire
U1065

1) “It is great that the authors increased the number of immunohistochemistries up to 8 and excluded necrosis. A more detailed explanation of the differences in LKB1 protein expression would be recommended.

The new 8 IHC, as the former ones, simply show that LKB1 expression is heterogeneous in metastatic uveal melanomas with at the two extreme, uveal melanoma cells displaying membranous LKB1 expression which has been associated with active LKB1 and uveal melanoma cells negative for LKB1. This is in agreement to what we indicated in our initial manuscript.

2) The authors claim that LKB1 activity can only be evaluated by their signature. However, correlation not always implies causation. LKB1 activity should be able to be measured by its direct downstream targets, such as AMPKα and AMPKα-like proteins (NUAK kinases, microtubule affinity regulating kinases [MARKs] and salt inducible kinases [SIK]).

As suggested by referee #1 in point 4, we have assessed the effect of LKB1 loss on P-mTOR, P-AMPK and P-S6 in metastatic uveal melanoma cells.

As referee #1 can appreciate, LKB1 loss in metastatic uveal melanoma cells does not affect their phosphorylation indicating that LKB1 loss in metastatic uveal melanoma does not function through the classical pathway **(see figure 2** and Appendix S3 in the revised manuscript**)**.

It is worth noting, that nor AMPK nor AMPK-like proteins were detected in our CRISPR/Cas9 screen.

Moreover, in this manuscript, we have shown in several cell lines that adding back SIK2 in LKB1-KO cells rescued the overproliferative phenotype mediated by LKB1 loss, thereby firmly demonstrating that LKB1 proliferative effect is mediated by SIK2 and not AMPK or an other AMPK-related member.

3) The rationale for selecting the 11 genes of the signature out of 27 genes still remains unclear.

The rationale for selecting the 11 genes is clearly explained. We first observed that a non-supervised analysis using the top 10 genes up-regulated after LKB1 loss from our transcriptomic data ranked by adjusted p-value shows a high prognosis value **(Figure 1)** (ROC AUC=0.85, $p<0.0001$).

Then, in view to improve the significance of this signature, the retained genes were then explored by using Cox regression model to calculate the hazard ratio (HR) leading to the identification of an 11-gene signature, with an even higher prognosis value.

We can’t be blamed for trying to find the best possible signature and to have the best clinical relevance.

In any cases, the important point is that in vitro LKB1 transcriptomic signature (top 10 unsupervised genes, or our 11 genes signature) has a high prognostic value in 2 different cohorts of primary uveal melanomas.

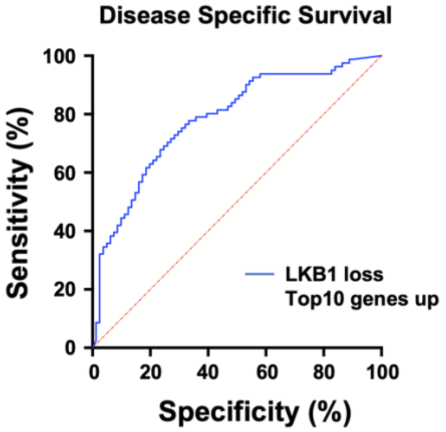

	LKB1 loss Top10 genes up
**AUC**	0.85
**p-value**	<0.0001

Figure 1: Time-dependent Receiver operating characteristic (ROC) curve show the sensitivity and specificity for the score of the Top10 upregulated genes upon LKB1 loss (RNA-seq data) ranked by adjusted p-value, for predicting the patient disease specific survival (UM-TCGA cohort).

4) It is claimed in this study that mTOR pathway is not activated after LKB1 loss in uveal melanoma. This is based on an unreliable marker of mTORC1 activity (mTOR phosphorylation). However, there is an overwhelming amount of evidence in the literature implying that mTORC1 signalling is hyperactivated after loss of LKB1 through multiple characterized mechanisms and is considered the major mechanism of how LKB1 loss contributes to oncogenesis (PMID: 29403037). At a minimum, the authors should show western blots with reliable markers of mTORC1 activity, such as pT389-S6K1 or pS6 (Ser235/236). Thereafter, it could be concluded whether LKB1 loss activates mTORC1 (or not) in uveal melanoma."
We agree with referee #1 that there is an overwhelming amount of evidence in the literature implying that mTORC1 signalling is hyperactivated after loss of LKB1. However, referee #1 might be aware that several publications also reported AMPK-independent LKB1 effect that can be explained by context and tissue-specific function of LKB1 (see also point 2).

A few examples of several publications showed that LKB1 effect is not mediated by the classical "AMPK" effector.
In cutaneous melanomas, while effect of LKB1 on proliferation is mediated by AMPK, LKB1 effect on haptotaxis is mediated by MARK (microtubule affinity-regulating kinase) (PMID: 25349262).
In lung cancer, the SIK family of kinases act redundantly, but collectively mediate LKB1-induced chromatin changes to drive metastatic progression (PMID: 34341533).

As suggested by referee #1 in point 4, we have assessed the effect of LKB1 loss on P-mTOR, P-AMPK and P-S6 in metastatic uveal melanoma cells.

Instituts thématiques Inserm
Institut national de la santé et de la recherche médicale
UNIVERSITÉ CÔTE D'AZUR

As referee #1 can appreciate, LKB1 loss does not affect their phosphorylation in metastatic uveal melanoma cells **(see figure 2** and Appendix S3 in the revised manuscript**)**.

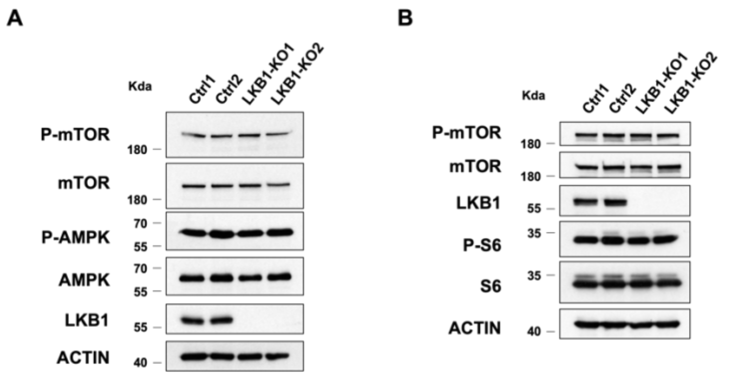

Figure 2. Signaling pathways in LKB1 KO metastatic uveal melanoma cells. (A) Immunoblot to LKB1, P-mTOR S2448, total mTOR, P-AMPK and total AMPK in the indicated whole-cell lysates of two different control (Ctl) OMM1.3 or LKB1-KO cells. **(B)** Immunoblot to LKB1, P-mTOR S2448, total mTOR, P-S6 S235/236 and total S6. B-Actin was used as a loading control.

6th Oct 2023

Dear Dr. Strub, dear Dr. Bertolotto,

Thank you for the submission of your manuscript to EMBO Molecular Medicine. We have now received the feedback from referee #1 on your revised manuscript. As you will see below, this referee remains skeptical that LKB1 activity is lost in uveal melanoma. However, after discussion with the team, we agreed that you had brought forward a number of arguments to address this point. We will therefore be able to accept your manuscript once the following will be addressed:

1/ Referee's comments:
- Please address the concern on LKB1 inactivation in primary uveal melanoma experimentally if possible, or in writing by an adequate discussion on the potential limitations of this work.
- Please provide details on serum concentration and address the referee's comment experimentally or in writing.
- Please follow this referee's instructions on gels presentation.

2/ Manuscript text:
- Please address the queries from our data editors in the Data edited file, remove the red text, and only keep in track changes mode any new modification.
- Please suggest up to 5 keywords.
- Please remove "data not shown" (p.9 and p.18): As per our guidelines on "Unpublished Data", the journal does not permit citation of "Data not shown". All data referred to in the paper should be displayed in the main or Expanded View figures.
- Material and methods:
o Cell cultures: please indicate the origin of the cells.
o Patient samples: Include a statement that the experiments conformed to the principles set out in the WMA Declaration of Helsinki and the Department of Health and Human Services Belmont Report
o Statistics: please include a sentence about randomization and correct the checklist accordingly.
- Acknowledgements: All funding sources should be added to the submission system, and match the information provided in the manuscript.

3/ Figures and Appendix:
- Please provide exact p values (including for ns, non-significant) in the figures or their legends.
- Appendix figures should be uploaded as a single appendix file containing a Table of Content.
- Tables 1-2 should be uploaded as Datasets and renamed Dataset EV1-2. Please correct the callouts in manuscript text accordingly.
- Table 3 can be made Table 1 (black and white), or if you want to keep it as is (with colors), it should become Table EV1 with its legend added to the file.
- Please indicate in the figure legends any figure reuse (i.e. Figures EV1C and EV3C, as well as EV1E and EV3D).

4/ In your point-by-point rebuttal letter, please include the referees' comments from the V2 version as well as you answer to these comments.

5/ I slightly edited your Paper Explained, let me know if you agree with the changes or amend as you see fit. Please add the TPE to your manuscript file.

The paper explained
Problem
Uveal melanoma is the most frequent primary malignant tumour of the eye in adults. Up to 50% of the patients develop distant metastases, with the liver as the most frequent site. Liver metastases are highly resistant to existing treatments, and patients die within a year from diagnosis. These clinical data highlight the importance of finding relevant therapeutic targets to improve patient outcome.
Results
A genetic screen in metastatic uveal melanoma cells led to the identification of the kinases LKB1 and SIK2, whose deficiency promoted a massive increase in their proliferation. Downstream LKB1 or SIK2 loss, increased expression of the sodium/calcium exchanger SLC8A1 was a key mediator of their pro-proliferative activity. Moreover, LKB1 or SIK2 deficient cells exhibited enhanced intracellular calcium and mitochondrial ROS, and were highly sensitive to a combination of SLC8A1 inhibitor and mitochondrial target anti-oxydant. Finally, we identified a LBK1 loss signature predictive of patient survival and of response to this combination therapy.
Impact
Our study shows for the first time that the module involving loss of LKB1 and SIK2 and increase in SLC8A1 strongly stimulates the proliferative activity of metastatic uveal melanoma cells. LKB1 or SIK2 loss triggered enhanced intracellular calcium and mitochondrial ROS, which could be targeted to induce metastatic uveal melanoma cell apoptosis. This combination therefore

represents a promising therapeutic strategy to treat metastatic uveal melanoma.

6/ Synopsis:
- Please upload your synopsis image as a jpeg, TIFF or png format and sized 550 pixels wide x 200-400 pixels high
- Let me know if you agree with the following minor changes in your synopsis text, or amend as you see fit:

Most patients with metastatic uveal melanoma die within a year from diagnosis, highlighting an unmet clinical need. A genetic screen was launched to identify actionable vulnerabilities and uncovered the critical role of the kinases LKB1 and SIK2 in constraining metastatic uveal melanoma cell growth.
-The sodium/calcium exchanger SLC8A1 mediated the effect of the LKB1-SIK2 module.
-Loss of the LKB1-SIK2 module and increased SLC8A1 expression were associated with enhanced mitochondrial level of calcium and ROS and increased metastatic uveal melanoma cell growth.
-SLC8A1 and mitochondrial ROS were validated as relevant therapeutic target as their combined inhibition induced cell death with a higher sensitivity in LKB1 or SIK2-depleted metastatic uveal melanoma cells.
-A molecular signature related to LKB1 loss was identified as a prognosis biomarker and a predictor of the response to SLC8A1 and mitochondrial ROS inhibition.

7/ As part of the EMBO Publications transparent editorial process initiative (see our Editorial at http://embomolmed.embopress.org/content/2/9/329), EMBO Molecular Medicine will publish online a Review Process File (RPF) to accompany accepted manuscripts.
This file will be published in conjunction with your paper and will include the anonymous referee reports, your point-by-point response and all pertinent correspondence relating to the manuscript. Let us know whether you agree with the publication of the RPF and as here, if you want to remove or not any figures from it prior to publication.
Please note that the Authors checklist will be published at the end of the RPF.

I look forward to receiving your revised manuscript.

Yours sincerely,

Lise Roth

Lise Roth, PhD
Senior Editor
EMBO Molecular Medicine

***** Reviewer's comments *****

Referee #1 (Comments on Novelty/Model System for Author):

This is a very nice study. However, this reviewer still has concerns about the suitability of its model system. Tumour suppressor LKB1 was found in a kinome CRISPR screen, but the evidence provided so far to support that LKB1 is inhibited/lost in uveal melanoma is not completely conclusive. Indeed, without inactivation of LKB1, xenografts implanted subcutaneously in mice grow very poorly.

Referee #1 (Remarks for Author):

Since LKB1 mutations are rare in uveal melanoma, it is essential to identify LKB1 inactivation in primary uveal melanoma tumours to validate the model system. IHC is a good approach, although the small numbers might be insufficient to draw conclusions. An alternative is RPPA for LKB1 from TCGA. In addition, the LKB1 activity should be quantified by measuring its direct downstream targets, such as AMPKα and AMPKα-like proteins (NUAK kinases, microtubule affinity regulating kinases [MARKs] and salt inducible kinases [SIK]), in RNAseq data from this study and TCGA (not in the CRISPR screen).

This study claims that the effects of LKB1 depletion are completely independent of mTORC1. Most likely, the cells were grown in high serum conditions, which already activate mTORC1 and might not be different that constitutively mTORC1 activation by

LKB1 loss. Thus, experiments should be done in low serum conditions, such as 0.5% or 1% FBS. To be even more certain, the cells could be treated with the mTORC1 inhibitor rapamycin and rule out if their growth is dependent on mTORC1 or not. If these experiments are not conducted, at least these limitations should be mentioned in the manuscript.

It is not appropriate to combine two gels from the same samples (Fig. 2 from the point-by-point response) into one panel without indicating that they are from different gels (Appendix S3). Please keep the gels separated for Appendix S3 or run another gel with all antibodies.

***** Reviewer's comments *****

Referee #1 (Comments on Novelty/Model System for Author):

This is a very nice study. However, this reviewer still has concerns about the suitability of its model system. Tumour suppressor LKB1 was found in a kinome CRISPR screen, but the evidence provided so far to support that LKB1 is inhibited/lost in uveal melanoma is not completely conclusive. Indeed, without inactivation of LKB1, xenografts implanted subcutaneously in mice grow very poorly. A short answer to this comment, indeed this observation shows that LKB1 inactivation is required for xenograft development, reinforcing our hypothesis that the loss of LKB1 favored uveal melanoma development.

Referee #1 (Remarks for Author):

Since LKB1 mutations are rare in uveal melanoma, it is essential to identify LKB1 inactivation in primary uveal melanoma tumours to validate the model system. IHC is a good approach, although the small numbers might be insufficient to draw conclusions. An alternative is RPPA for LKB1 from TCGA. In addition, the LKB1 activity should be quantified by measuring its direct downstream targets, such as AMPKα and AMPKα-like proteins (NUAK kinases, microtubule affinity regulating kinases [MARKs] and salt inducible kinases [SIK]), in RNAseq data from this study and TCGA (not in the CRISPR screen).
We agree with the reviewer that LKB1 activity can be monitored by its downstream targets in RPPA. However, in the publicly available RPPA datasets for uveal melanomas there are only 12 tumors and limited or no information about LKB1 downstream targets. So, it is not possible to get useful information. Related to the TCGA RNAseq dataset, this brings limited information about LKB1 activity as the downstream known direct targets of LKB1 are not regulated at the transcriptional level. Again, our signature identified a list of genes regulated by LKB1 loss. As suggested by the referee, we used this gene list to analyse the TCGA RNA-seq and found that it is valuable for the prognosis of patient survival.
Whatever, SIK2 reexpression in LKB1 KO cells, fully rescues the LKB1 loss hyperproliferative phenotype, demonstrating the critical role of SIK2, compared to other LKB1 target, downstream LKB1.

This study claims that the effects of LKB1 depletion are completely independent of mTORC1. Most likely, the cells were grown in high serum conditions, which already activate mTORC1 and might not be different that constitutively mTORC1 activation by LKB1 loss. Thus, experiments should be done in low serum conditions, such as 0.5% or 1% FBS. To be even more certain, the cells could be treated with the mTORC1 inhibitor rapamycin and rule out if their growth is dependent on mTORC1 or not. If these experiments are not conducted, at least these limitations should be mentioned in the manuscript.
As requested by the referee we discussed this point in the manuscript as follow:
“One can argue that in culture conditions with high serum concentration the effects of LKB1 KO on mTOR phosphorylation is masked. However, in these same conditions LKB1 KO is able to promote uveal melanoma cell proliferation indicating that the pro-proliferative effect of LKB1 KO is not mediated through mTOR activation.”

This paragraph has been included in the discussion section p20 of the revised manuscript.

Moreover, in this condition, LKB1 KO completely abolished SIK2 phosphorylation, thereby indicating that even in presence of high serum concentration the LKB1-SIK2 module is not constitutively regulated. Finally, as previously indicated, SIK2 reintroduction in LKB1 KO cells, rescued the LKB1 KO overproliferative phenotype.

Collectively these observations indicate that LKB1 effect is mediated by SIK2 in the context of uveal melanoma and not by mTORC1.

Serum concentration is indicated in the materials and methods, section paragraph cell cultures.

It is not appropriate to combine two gels from the same samples (Fig. 2 from the point-by-point response) into one panel without indicating that they are from different gels (Appendix S3). Please keep the gels separated for Appendix S3 or run another gel with all antibodies.

In the revised version (new Appendix S3), we show the gels separated.

23rd Oct 2023

Dear Dr. Strub,

Thank you for bearing with the last editorial considerations. I am pleased to inform you that your manuscript is accepted for publication and is now being sent to our publisher to be included in the next available issue of EMBO Molecular Medicine!

Please read below for additional IMPORTANT information regarding your article, its publication and the production process.

Congratulations on your interesting work,

With kind regards,

Lise Roth

Lise Roth, Ph.D
Senior Editor
EMBO Molecular Medicine

Follow us on Twitter @EmboMolMed
Sign up for eTOCs at embopress.org/alertsfeeds

*** *** *** IMPORTANT INFORMATION *** *** ***

SPEED OF PUBLICATION
The journal aims for rapid publication of papers, using using the advance online publication "Early View" to expedite the process: A properly copy-edited and formatted version will be published as "Early View" after the proofs have been corrected. Please help the Editors and publisher avoid delays by providing e-mail address(es), telephone and fax numbers at which author(s) can be contacted.

Should you be planning a Press Release on your article, please get in contact with embomolmed@wiley.com as early as possible, in order to coordinate publication and release dates.

LICENSE AND PAYMENT:

All articles published in EMBO Molecular Medicine are fully open access: immediately and freely available to read, download and share.

EMBO Molecular Medicine charges an article processing charge (APC) to cover the publication costs. You, as the corresponding author for this manuscript, should have already received a quote with the article processing fee separately. Please let us know in case this quote has not been received.

Once your article is at Wiley for editorial production you will receive an email from Wiley's Author Services system, which will ask you to log in and will present you with the publication license form for completion. Within the same system the publication fee can be paid by credit card, an invoice, pro forma invoice or purchase order can be requested.

Payment of the publication charge and the signed Open Access Agreement form must be received before the article can be published online.

PROOFS

You will receive the proofs by e-mail approximately 2 weeks after all relevant files have been sent o our Production Office. Please return them within 48 hours and if there should be any problems, please contact the production office at embopressproduction@wiley.com.

Please inform us if there is likely to be any difficulty in reaching you at the above address at that time. Failure to meet our deadlines may result in a delay of publication.

All further communications concerning your paper proofs should quote reference number EMM-2023-17719-V4 and be directed to the production office at embopressproduction@wiley.com.

Thank you,

Lise Roth, Ph.D
Senior Editor
EMBO Molecular Medicine

EMBO Press Author Checklist

Corresponding Author Name: Corine BERTOLOTTO
Journal Submitted to: EMBO Mol Med
Manuscript Number: EMM-2023-17719

USEFUL LINKS FOR COMPLETING THIS FORM
The EMBO Journal - Author Guidelines
EMBO Reports - Author Guidelines
Molecular Systems Biology - Author Guidelines
EMBO Molecular Medicine - Author Guidelines

Reporting Checklist for Life Science Articles (updated January
This checklist is adapted from Materials Design Analysis Reporting (MDAR) Checklist for Authors. MDAR establishes a minimum set of requirements in transparent reporting in the life sciences (see Statement of Task: 10.31222/osf.io/9sm4x). Please follow the journal's guidelines in preparing your
Please note that a copy of this checklist will be published alongside your article.

Abridged guidelines for figures
1. Data
The data shown in figures should satisfy the following conditions:
- the data were obtained and processed according to the field's best practice and are presented to reflect the results of the experiments in an accurate and unbiased manner.
- ideally, figure panels should include only measurements that are directly comparable to each other and obtained with the same assay.
- plots include clearly labeled error bars for independent experiments and sample sizes. Unless justified, error bars should not be shown for technical
- if n<5, the individual data points from each experiment should be plotted. Any statistical test employed should be justified.
- Source Data should be included to report the data underlying figures according to the guidelines set out in the authorship guidelines on Data

2. Captions
Each figure caption should contain the following information, for each panel where they are relevant:
- a specification of the experimental system investigated (eg cell line, species name).
- the assay(s) and method(s) used to carry out the reported observations and measurements.
- an explicit mention of the biological and chemical entity(ies) that are being measured.
- an explicit mention of the biological and chemical entity(ies) that are altered/varied/perturbed in a controlled manner.
- the exact sample size (n) for each experimental group/condition, given as a number, not a range;
- a description of the sample collection allowing the reader to understand whether the samples represent technical or biological replicates (including how many animals, litters, cultures, etc.).
- a statement of how many times the experiment shown was independently replicated in the laboratory.
- definitions of statistical methods and measures:
  - common tests, such as t-test (please specify whether paired vs. unpaired), simple χ2 tests, Wilcoxon and Mann-Whitney tests, can be unambiguously identified by name only, but more complex techniques should be described in the methods section;
  - are tests one-sided or two-sided?
  - are there adjustments for multiple comparisons?
  - exact statistical test results, e.g., P values = x but not P values < x;
  - definition of 'center values' as median or average;
  - definition of error bars as s.d. or s.e.m.

Please complete ALL of the questions below.
Select "Not Applicable" only when the requested information is not relevant for your study.

Materials

Newly Created Materials	Information included in the manuscript?	In which section is the information available? (Reagents and Tools Table, Materials and Methods, Figures, Data Availability Section)
New materials and reagents need to be available; do any restrictions apply?	Yes	Materials and Methods; no restriction applied

Antibodies	Information included in the manuscript?	In which section is the information available? (Reagents and Tools Table, Materials and Methods, Figures, Data Availability Section)
For **antibodies** provide the following information:<br>- Commercial antibodies: RRID (if possible) or supplier name, catalogue number and or/clone number<br>- Non-commercial: RRID or citation	Yes	Materials and Methods

DNA and RNA sequences	Information included in the manuscript?	In which section is the information available? (Reagents and Tools Table, Materials and Methods, Figures, Data Availability Section)
**Short novel DNA or RNA including primers, probes**: provide the sequences.	Yes	Materials and Methods

Cell materials	Information included in the manuscript?	In which section is the information available? (Reagents and Tools Table, Materials and Methods, Figures, Data Availability Section)
**Cell lines:** Provide species information, strain. Provide accession number in repository **OR** supplier name, catalog number, clone number, and/**OR** RRID.	Yes	Materials and Methods
**Primary cultures:** Provide species, strain, sex of origin, genetic modification status.	Not Applicable	
Report if the cell lines were recently **authenticated** (e.g., by STR profiling) and tested for mycoplasma contamination.	Yes	Materials and Methods

Experimental animals	Information included in the manuscript?	In which section is the information available? (Reagents and Tools Table, Materials and Methods, Figures, Data Availability Section)
**Laboratory animals or Model organisms:** Provide species, strain, sex, age, genetic modification status. Provide accession number in repository **OR** supplier name, catalog number, clone number, **OR** RRID.	Yes	Materials and Methods
**Animal observed in or captured from the field:** Provide species, sex, and age where possible**.**	Not Applicable	
Please detail **housing and husbandry conditions**.	Yes	Materials and Methods

Plants and microbes	Information included in the manuscript?	In which section is the information available? (Reagents and Tools Table, Materials and Methods, Figures, Data Availability Section)
**Plants:** provide species and strain, ecotype and cultivar where relevant, unique accession number if available, and source (including location for collected wild specimens).	Not Applicable	
**Microbes:** provide species and strain, unique accession number if available, and source.	Not Applicable	

Human research participants	Information included in the manuscript?	In which section is the information available? (Reagents and Tools Table, Materials and Methods, Figures, Data Availability Section)
If collected and within the bounds of privacy constraints report on age, sex and gender or ethnicity for all study participants.	Not Applicable	

Core facilities	Information included in the manuscript?	In which section is the information available? (Reagents and Tools Table, Materials and Methods, Figures, Data Availability Section)
If your work benefited from core facilities, was their service mentioned in the acknowledgments section?	Yes	The authors thank the C3M imaging facility (INSERM U1065).

Design

Study protocol	Information included in the manuscript?	In which section is the information available? (Reagents and Tools Table, Materials and Methods, Figures, Data Availability Section)
If study protocol has been **pre-registered, provide DOI in the manuscript**. For clinical trials, provide the trial registration number **OR** cite DOI.	Not Applicable	
Report the **clinical trial registration number** (at ClinicalTrials.gov or equivalent), where applicable.	Not Applicable	

Laboratory protocol	Information included in the manuscript?	In which section is the information available? (Reagents and Tools Table, Materials and Methods, Figures, Data Availability Section)
Provide DOI OR other citation details if **external detailed step-by-step protocols** are available.	Not Applicable	

Experimental study design and statistics	Information included in the manuscript?	In which section is the information available? (Reagents and Tools Table, Materials and Methods, Figures, Data Availability Section)
Include a statement about **sample size** estimate even if no statistical methods were used.	Yes	Materials and Methods
Were any steps taken to minimize the effects of subjective bias when allocating animals/samples to treatment (e.g. **randomization procedure**)? If yes, have they been described?	Yes	Materials and Methods
Include a statement about **blinding** even if no blinding was done.	Yes	Materials and Methods
Describe **inclusion/exclusion criteria** if samples or animals were excluded from the analysis. Were the criteria pre-established? If sample or data points were omitted from analysis, report if this was due to attrition or intentional exclusion and provide justification.	Yes	Materials and Methods
For every figure, are **statistical tests** justified as appropriate? Do the data meet the assumptions of the tests (e.g., normal distribution)? Describe any methods used to assess it. Is there an estimate of variation within each group of data? Is the variance similar between the groups that are being statistically compared?	Yes	Materials and Methods; Figures

Sample definition and in-laboratory replication	Information included in the manuscript?	In which section is the information available? (Reagents and Tools Table, Materials and Methods, Figures, Data Availability Section)
In the figure legends: state number of times the experiment was **replicated** in laboratory.	Yes	Figures
In the figure legends: define whether data describe **technical or biological replicates**.	Yes	Materials and Methods; Figures

Ethics

Ethics	Information included in the manuscript?	In which section is the information available? (Reagents and Tools Table, Materials and Methods, Figures, Data Availability Section)
Studies involving **human participants**: State details of **authority granting ethics approval** (IRB or equivalent committee(s), provide reference number for approval.	Yes	Materials and Methods
Studies involving **human participants**: Include a statement confirming that **informed consent** was obtained from all subjects and that the experiments conformed to the principles set out in the WMA Declaration of Helsinki and the Department of Health and Human Services Belmont Report.	Yes	Materials and Methods
Studies involving **human participants:** For publication of **patient photos**, include a statement confirming that consent to publish was obtained.	Not Applicable	
Studies involving experimental **animals**: State details of **authority granting ethics approva**l (IRB or equivalent committee(s), provide reference number for approval. Include a statement of compliance with ethical regulations.	Yes	Materials and Methods
Studies involving **specimen and field samples:** State if relevant **permits** obtained, provide details of authority approving study; if none were required, explain why.	Not Applicable	

Dual Use Research of Concern (DURC)	Information included in the manuscript?	In which section is the information available? (Reagents and Tools Table, Materials and Methods, Figures, Data Availability Section)
Could your study fall under dual use research restrictions? Please check biosecurity documents and list of **select agents and toxins** (CDC): https://www.selectagents.gov/sat/list.htm	Not Applicable	
If you used a select agent, is the security level of the lab appropriate and reported in the manuscript?	Not Applicable	
If a study is subject to dual use research of concern regulations, is the name of the **authority granting approval and reference number** for the regulatory approval provided in the manuscript?	Not Applicable	

Reporting

The MDAR framework recommends adoption of discipline-specific guidelines, established and endorsed through community initiatives. Journals have their own policy about requiring specific guidelines and recommendations to complement MDAR.

Adherence to community standards	Information included in the manuscript?	In which section is the information available? (Reagents and Tools Table, Materials and Methods, Figures, Data Availability Section)
State if relevant guidelines or checklists (e.g., **ICMJE, MIBBI, ARRIVE, PRISMA**) have been followed or provided.	Not Applicable	
For **tumor marker prognostic studies,** we recommend that you follow the **REMARK** reporting guidelines (see link list at top right). See author guidelines, under 'Reporting Guidelines'. Please confirm you have followed these guidelines.	Not Applicable	
For **phase II and III randomized controlled trials**, please refer to the **CONSORT** flow diagram (see link list at top right) and submit the CONSORT checklist (see link list at top right) with your submission. See author guidelines, under 'Reporting Guidelines'. Please confirm you have submitted this list.	Not Applicable	

Data Availability

Data availability	Information included in the manuscript?	In which section is the information available? (Reagents and Tools Table, Materials and Methods, Figures, Data Availability Section)
Have **primary datasets** been deposited according to the journal's guidelines (see 'Data Deposition' section) and the respective accession numbers provided in the Data Availability Section?	Yes	The RNA-sequencing data generated and/or analyzed during this study have been deposited in the NCBI Gene Expression Omnibus (GEO) database (https://www.ncbi.nlm.nih.gov/geo/)
Were **human clinical and genomic datasets** deposited in a public access-controlled repository in accordance to ethical obligations to the patients and to the applicable consent agreement?	Not Applicable	
Are **computational models** that are central and integral to a study available without restrictions in a machine-readable form? Were the relevant accession numbers or links provided?	Not Applicable	
If publicly available data were reused, provide the respective **data citations in the reference list**.	Yes	Figures
